# A specific, non-immune system-related isoform of the human inducible nitric oxide synthase is expressed during differentiation of human stem cells into various cell types

**DOI:** 10.1186/s12964-022-00855-x

**Published:** 2022-04-07

**Authors:** Fabian Gather, Irmgard Ihrig-Biedert, Paul Kohlhas, Tamara Krutenko, Michael Peitz, Oliver Brüstle, Andrea Pautz, Hartmut Kleinert

**Affiliations:** 1 Department of Pharmacology, University Medical Center of the Johannes Gutenberg University Mainz, Langenbeckstr. 1, 55131 Mainz, Germany; 2 Cell Programming Core Facility, Institute of Reconstructive Neurobiology, University of Bonn Medical Faculty & University Hospital Bonn, Bonn, Germany; 3 Institute of Reconstructive Neurobiology, University of Bonn Medical Faculty & University Hospital Bonn, Bonn, Germany; 4 Department of Molecular Embryology, Institute for Anatomy and Cell Biology, Freiburg, Germany

**Keywords:** iNOS, NOS2, hPSCs

## Abstract

**Background:**

NOS2 expression is mostly found in bacteria-exposed or cytokine-treated tissues and is mostly connected to innate immune reactions. There are three isoforms of NOS2 (NOS2-1 to -3). In RNA-seq data sets, analyzing inflammatory gene expression, only expression of the NOS2-1 mRNA isoform is detected. However, the expression of NOS2 in differentiating human pluripotent stems (hPSCs) has not been analyzed yet.

**Methods:**

Public available RNA-seq databases were screened for data of hPSCs during differentiation to different target cells. An isoform specific algorithm was used to analyze NOS2 mRNA isoform expression. In addition, we differentiated four different human iPSC cell lines toward cortical neurons and analyzed NOS2 mRNA expression by qRT-PCR and 5′-RACE. The functionality of the NOS2-2 protein was analyzed by transient transfection of expression clones in human DLD1 cells and nitrate measurement in the supernatant of these cells.

**Results:**

In RNA-seq databases we detected a transient expression of the NOS2 mRNA during the differentiation of hPSCs to cardiomyocytes, chondrocytes, mesenchymal stromal cells, neurons, syncytiotrophoblast cells, and trophoblasts. NOS2 mRNA isoform specific analyses showed, that the transiently expressed NOS2 mRNA in differentiating hPSC (NOS2-2; “diff-iNOS”) differ remarkably from the already described NOS2 transcript found in colon or induced islets (NOS2-1; “immuno-iNOS”). Also, analysis of the NOS2 mRNA- and protein expression during the differentiation of four different hiPSC lines towards cortical neurons showed a transient expression of the NOS2 mRNA and NOS2 protein on day 18 of the differentiation course. 5′-RACE experiments and isoform specific qRT-PCR analyses revealed that only the NOS2-2 mRNA isoform was expressed in these experiments. To analyze the functionality of the NOS2-2 protein, we transfected human DLD-1 cells with tetracycline inducible expression clones encoding the NOS2-1- or -2 coding sequence. After induction of the NOS2-1 or -2 mRNA expression by tetracycline a similar nitrate production was measured proofing the functionality of the NOS2-2 protein isoform.

**Conclusions:**

Our data show that a differentiation specific NOS2 isoform (NOS2-2) is transiently expressed during differentiation of hPSC.

**Video Abstract**

**Supplementary Information:**

The online version contains supplementary material available at 10.1186/s12964-022-00855-x.

## Background

NO is synthesized by many organisms ranging from bacteria [1], yeast [2] and invertebrates [3] to mammals. Although chemically simple, this molecule can act in a fairly specific manner controlling vital functions such as vascular tone, platelet aggregation, leucocyte adhesion, smooth muscle cell contraction and the contraction of gastrointestinal organs, neurotransmission, as well as regulation of stem cell renewal and differentiation, mainly via activation of soluble guanylyl cyclase (sGC) [4, 5]. Further, by activation or deactivation of transcription factors NO can affect gene transcription [6, 7] and mRNA translation (e.g., via iron-responsive elements) [8].

Higher concentrations of the radical are capable of destroying bacteria, parasites and certain tumor cells by inhibiting iron-containing enzymes [9], directly interacting with the DNA of these cells [10, 11], or producing post-translational modifications of proteins via for example S-Nitrosothiol adduct formation [12] or ADP-ribosylation [13].

From the three isozymes of nitric oxide synthase (NOS) identified in mammals, NOS2 originally discovered in cytokine-induced macrophages [14, 15], is a high output enzyme, which produces high (sometimes toxic) amounts of NO that represent an important component of the antimicrobial, antiparasitic and antineoplastic activity of innate immune cells. Depending on the species, NOS2 activity is largely (human) or completely (mouse and rat) Ca^2+^-independent.

Human pluripotent stem cells (hPSCs) are able to give rise of all cell types in the human body and can be either isolated from human embryos (from human blastocysts; human embryonic stem cells; hESCs, [16]) or derived from human somatic cells [17] (named human induced pluripotent stem cells; hiPSCs) by forced expression of transcription factors, which typically comprise octamer binding transcription factor 4—OCT4, sex-determining region Y-box 2—SOX2, Kruppel Like Factor 4—KLF4, and the oncogene c-MYC—often referred as OSKM as firstly published by Takahashi et al. for the murine system [18]. Beside their usefulness in regenerative medicine, hiPSCs derived from patients are important tools to analyze the molecular mechanisms of diseases and to test new pharmaceutical compounds for treatment of these diseases in a human system [19].

The mechanism and factors (growth factors, signaling molecules etc.) for the targeted differentiation of hiPSCs into the cell type of interest are currently investigated [20]. This research provides evidence that gaseous signaling molecules especially nitric oxide (NO) centrally modulate stem cell behavior, including survival, migration, differentiation, and paracrine secretion of proregenerative factors [5, 21]. In the murine system low concentrations of NO seem to favor SC renewal [22] whereas higher concentrations induce differentiation by repression of the transcription factor Nanog [23].

But NO has not only been described to have impact on stem cell properties, it has also an important impact on neuronal differentiation [24]. Haghighat et al. showed that enhanced NO concentrations in bone marrow (BM) derived mesenchymal SC (BM-MSCs) of rats resulted in enhanced expression of marker genes (nestin and DCX) of neuronal differentiation and morphogenic changes to neurons [25]. On the other hand, there are reports of teratogenic effects of too high or too low NO levels in the course of embryonic development [26–28]. Also, overexpression of different NOS isoforms [29] resulted in the perturbation of the proliferation of neuronal stem cells (NSC) and neuronal progenitor cells (NPC). For example, the *leukemia inhibitory factor* (LIF) induced expression of nitric oxide synthase 2 (NOS2, iNOS) in in olfactory epithelial cells, olfactory NPCs and neurosphere cultures resulted in enhanced proliferation. Addition of a LIF- or NOS2 inhibitor reduced proliferation, an effect which could be reverted by incubation with a NO donor [30]. Also, a relation between the expression of the stem cell marker sex determining region Y-box 2 (SOX2) and NOS2 expression was described. Both proteins are expressed only transiently in specific cells at specific times [31]. This time dependence could be also observed in the development of olfactory [32] and vestibuliocculary receptor cells of mice [33, 34]. However, the exact effects of NOS expression or activity in the neuronal differentiation are not clear.

In vascular repair processes, endothelial progenitor cells (EPCs) can differentiate to functional endothelial cells (ECs) and replace damaged cells [35]. NO and NO-mediated pathways have been shown to upregulate the numbers of circulating EPC (mobilization and migration).

Mesenchymal stem cells (MSCs) are adult stem cells able to differentiate into chondrocytes, osteoblasts, and adipocytes. NO has been shown to positively or negatively regulate the ability of MSC to migrate and to promote the homing of BM MSC by enhancement of the expression of the chemoattractant stromal cell-derived factor-1 alpha (SDF-1) [21]).

Mammalian hearts contain resident tissue-specific cardiac stem/progenitor cells (CSC) [36]. Pretreatment of CSC with NO donors has reported to enhance cell survival [21]. Cytoglobin (CYGB), a mammalian globin expressed in hCSCs, has been described to regulate NO metabolism and cell death [37]. CYGB is expressed in hCSCs and upregulates the expression of NF-κB regulated genes like NOS2. CYGB expression was related to hCSC survival and this cytoprotective effect was lost after downregulation of NOS2 expression or activity [37].

Embryo implantation into the endometrium depends on enhanced vascular permeability, edema, altered membrane fluidity, and programmed epithelial cell death in response to blastocyst adhesion [38]. NO prepares the endometrium for this process by inducing vasodilation, immune function, and inflammation. NO has been shown to be important for human and mouse trophoblast differentiation and survival. Along with this data, analysis of human NOS2 expression (https://www.proteinatlas.org/) shows a significant expression of NOS2 in the placenta (trophoblast cells; see Additional file 1: Fig. S1).

All these data imply that NO plays an important role in SC differentiation processes, but only very limited information about the nature of NOS2 involved in these processes exist so far.

Expression of NOS2 has been described to be mainly regulated at the expressional level and can be induced in many cell types with suitable agents such as LPS, cytokines, and other compounds mostly secreted by the innate immune system [39]. A “constitutive” expression of NOS2 has been described for epithelial cells of the colon and lungs, which is likely “induced” by the microbiota in these organs, and spinal tissue of the brain and for different human cancer cells [40]. The expressional regulation of NOS2 is mediated by different mechanisms and pathways resulting in the induction of the NOS2 promoter and seem to vary in different cells. These include changes in chromatin packaging [41, 42], mediated by histone methylation/acetylation [43], effects of long non-coding RNAs (ncRNAs), and activation/inhibition of transcription factors (in most cells NF-κB and STAT-1α). In addition, post-transcriptional regulation is important for the modulation of human NOS2 expression. The post-transcriptional mechanisms involved, include modulation of mRNA-splicing [44], -localization [45], and RNA binding protein- and/or micro RNA-regulated mRNA-stability [46], as well as RNA-translatability [46–48]. In the end also NOS2 protein stability and activity is regulated by different factors [46].

Analyzing endogenous NOS2 expression using the human protein atlas (https://www.proteinatlas.org/ENSG00000007171-NOS2/celltype) shows high expression of NOS2 mRNA in placenta extravillous trophoblast and colon (small intestine, rectum) enterocytes (see Additional file 1: Fig. S1). Also, Fagerberg et al. described high NOS2 expression in human small intestine, appendix, duodenum, urinary bladder, colon, and lung [49].

In the current study we analyzed the expression of human NOS2 using public available RNA-Seq data from different cell types and tissues. As expected, we observed high NOS2 mRNA expression in human small intestine and sigmoid colon tissues. Also, in human isolated pancreatic islets high NOS2 mRNA expression was induced by cytokine treatment. In addition, our bioinformatic analyses showed a temporary expression of human NOS2 mRNA in the differentiation of hESC or hiPSC to cardiomyocytes, chondrocytes mesenchymal stromal cells, neurons, syncytiotrophoblast cells, and trophoblasts. The structure of the temporarily expressed NOS2 mRNA in hESC or hiPSC (NOS2-2) is different from the NOS2 mRNA (NOS2-1) commonly expressed in immune, colon or islets cells (Table 1).

**Table 1:** NOS2 transcripts in the ENSEMBL database

Name (ENS-EMBL)	Name (CLC)	Transcript ID	nt	Protein aa	Translation ID	Uni-Prot match
NOS2-201	NOS2-1	ENST00000313735.11	4206	1153	ENSP00000327251.6	P35228-1
NOS2-203	NOS2-2	ENST00000646938.1	3995	1152	ENSP00000494870.1	A0A2R8YDS4
NOS2-202	NOS2-3	ENST00000621962.1	3345	1114	ENSP00000482291.1	P35228-2

Shown are the Names (ENSEMBL and CLC genomic workbench), transcript ID, mRNA length in nucleotides (nt), the protein length in number of amino acids (aa), the translation ID, and the UniProt Match as depicted by the ENSEMBL database (https://www.ensembl.org/Homo_sapiens/Gene/Summary?db=core;g=ENSG00000007171;r=17:27756766-27800529)

## Methods

### Materials

Trypsin-, glutamine-, and pyruvate-solutions, FGF-2, Cytosine β-d-arabinofuranoside hydrochloride and agarose were purchased from Sigma, Deisenhofen, Germany. The monoclonal anti-iNOS antibody (human, mouse, rat; MaB9502) was obtained from R&D Systems, Inc., Minneapolis, U.S.A. The monoclonal anti-GAPDH antibody (human, mouse, rat, rabbit, xenopus; Sc 32233) was obtained from Santa Cruz Biotechnology, Inc., Heidelberg Germany. Calf intestine alkaline phosphatase were obtained from Roche Diagnostics, Mannheim, Germany. The GeneJuice transfection reagent was from Merck, Darmstadt, Germany. Restriction enzymes, Taq polymerase, Q5^®^ High-Fidelity DNA Polymerase, Klenow DNA polymerase and dNTPs were purchased from New England Biolabs, Frankfurt, Germany. All oligonucleotides and dual labeled probes were from Sigma, Deisenhofen, Germany. Human interferon-γ (IFN-γ), interleukin-1β (IL-1β), tumor necrosis factor-α (TNF-α), and StemMACS^™^ were obtained from Miltenyi Biotec, Bergisch Gladbach, Germany. The High-Capacity cDNA Reverse Transcription Kit was purchased from Applied Biosystems, Darmstadt, Germany. The PrecisionPLUS 2x qPCR MasterMix with SYBR green was obtained from Primer Design, Chandler's Ford, United Kingdom. The QuikChange II Site-Directed Mutagenesis Kit was from Agilent Technologies, Waldbronn, Germany. FCS and DMEM and DMEMF12 were purchased from PAN-Systems, Nürnberg, Germany. Zeocin and blasticidin were purchased from Invivogen, San Diego, USA. pcDNA4/TO and pcDNA6/TR were purchased from Invitrogen, Groningen, The Netherlands. The Dual-Luciferase Reporter Assay System, Passive Lysis Buffer, pGL3control, and Griess reagent system were purchased from Promega, Heidelberg, Germany. mTeSR and accutase were obtained from Stem Cell Technologies, Köln, Germany. Y-27632, dorsomorphin, LDN-193189, PD0325901, DAPT, and SB431542 was obtained from Tocris, Wiesbaden, Germany. Neurobasal medium, N2 and B27 supplement was obtained from Thermo Fisher Scientific, Waltham, U.S.A. The pRL-EF1α vector [50] was a kind gift of Dr. M. Bros (Department of Dermatology, University Medical Center of the Johannes Gutenberg University Mainz, Mainz, Germany).

### 5′-RACE

5′-RACE was performed using the 5′/3′ RACE Kit, 2nd Generation (Sigma Aldrich, Munic Germany) following the recommendations of the manufacturer. For the first step (mRNA-specific cDNA reaction) the human iNOS mRNA specific primer hNOS2-rev (5′-GGTAGC CAGCATAGCGGATG-3′) was used. For the PCR reactions the *AllTaq Master Mix Kit* provided by Qiagen (Hilden, Germany) was used. The resulting PCR fragments were purified, cloned into pCR-Script (Agilent Technologies, Corston, U.K.) and sequenced (Starseq, Mainz, Germany).

### Analysis of public RNA-Seq data

All analyses of public RNA-Seq data (see Table 2) were performed with CLC genomic workbench 21.0.5 from Qiagen (Hilden, Germany; see manual: https://resources.qiagenbioinformatics.com/manuals/clcmainworkbench/current/index.php?manual=Introduction_CLC_Main_Workbench.html) using the parameters provided by the manufacturer.

**Table 2:** RNA-Seq data used in the bio-informatic analyses

Accession-Nr.	Description	Lit. or submitter	Database
CNP0000771	hu-iPSC (from human dermal fibroblasts GM01450) and H9 into iPSC-iMSC and H9ES-iMSC	[63]	CNGBdb
PRJDB1099	hiPSC from normal and trisomy 21 donors (FANTOM) into neurons (motoneurons, dopaminergic neurons) (CAGE seq)	[64]	NCBI BioProject
PRJNA244622	H9 (WA-09, WiCell) into prefrontal cortex neurons	[73]	NCBI BioProject
PRJNA404971	H9 into neurons	[74]	NCBI BioProject
PRJNA414247	H9 (WA09) into trophectoderm cells	[75]	NCBI BioProject
PRJNA433877	H9 into rostrocaudal neurons	[76]	NCBI BioProject
PRJNA484413	H9 into endothelial cells	[77]	NCBI BioProject
PRJNA544617	H9 to EC (two protocols)	[78]	NCBI BioProject
PRJNA596331	hiPSC into neurons	Lieber Institute	NCBI BioProject
PRJNA645819	hiPSC (CD34-iPSC) into cardiomyocytes	[60]	NCBI BioProject
PRJNA674506	hiPSC (ATCC, BJFF, STAN) into chondrocytes	[62]	NCBI BioProject

Shown are the database accession numbers, a short description of the study, the paper published and the database name

The data (fastq.gz) were downloaded from the public servers (maintaining the quality scores and read names) and imported in the CLC data format (.clc). Then the reads were trimmed using the parameter provided by the manufacturer (quality score: 0.05; maximum number of ambiguities: 2). These trimmed RNA-Seq data were mapped to the human genome (Homo_sapiens_hg38-2020-12-10-08-41, ENSEMBL) using the parameters provided by the manufacturer (Mismatch cost: 2; Insertion cost: 3; Deletion cost: 3; Length fraction: 0.8; Similarity fraction: 0.8; Maximum number of hits for a read: 10). Also, the parameters used for the calculation of transcript expression were used as provided by the program (Strand setting: Both; Library type setting: Bulk) and rpkm data [51] were calculated. The rpkm data were used for calculation of the fold enhancement of the mRNA expression.

For clc genomic workbench blast analyses the following parameters were used: Number of threads: 16; Mask low complexity regions: yes; Expect: 0.0001; Word size: 48; Match:2; Mismatch: 3; Gap costs: Existence: 5, Extension 2; Max number of hit sequences: 500,000; Filter out redundant results: no.

### Plasmid constructs (see Additional file 1: Fig. S2 for maps)

To reduce mutations in the sequence of PCR fragments the Q5^®^ High-Fidelity DNA Polymerase from NEB was used in all PCR reactions described.

To generate pcDNA4/TO construct containing the cds and the 3′-UTR of the human NOS2-1 RNA from cytokine-induced DLD-1 cells were isolated. This RNA was reverse transcribed to cDNA. Then PCR reactions were performed using the oligonucleotides NOS2-1_5P (5′-GATCTCGAGGAGATGGCCTGTCCTTG-3′ and NOS2-2_3P (5′-CCTCTAGAGCTTTGATTAAAGTAAAATGC-3′) as primers and the cDNA as template of the reactions. The resulting PCR fragments were restricted with Xba I and Xho I and cloned into pcDNA4/TO (restricted with Xba I and Xho I) generating the plasmid pcDNA4/TO-NOS2-1_cds_3UTR.

To obtain a pcDNA4/TO construct containing the exon 1-diff instead of exon 1 and 2 PCR reactions were performed using the oligonucleotides Ex1-diff_5P (5′-GTACCGAGCTCGGATCTCGAGAGGCGCGTGGAGCCAGCGG-3′) and Ex1-diff_3P (5′-GGTCATCCTGTGTCACTGGACTGGCTCTGCGCGGGCAGC-3′) as primers and human chromosomal DNA isolated from DLD-1 cells as template. The resulting PCR-fragment (Ex1-diff_5P_3P) was used as the primer in QuikChange reactions with pcDNA4/TO-NOS2-1_cds_3UTR as template. This generated the plasmid pcDNA4/TO_NOS2-2_cds_3UTR.

The relevant DNA sequences of all plasmids were determined using the dideoxy chain termination method (Starseq, Mainz, Germany).

### Cell lines used

**Table:** 

Name	Description	Media	Source
DLD1	Human epithelial coloncarcinoma cells	DMEM with 10% inactivated fetal bovine serum, 2 mM l-glutamine, penicillin and streptomycin	ATCC, #CCL-221
DLD-1_TR7	Human epithelial coloncarcinoma cells stably transfected with pcDNA6/TR. The cells express a tetracycline repressor	DMEM with 10% inactivated fetal bovine serum, 2 mM l-glutamine, penicillin, streptomycin, and blasticillin	Generated in this study
iLB-C16bm-s6 UKBi015-A in hSPCreg	hiPSC line generated from PBMC of a male donor (C16bm)	StemMACS iPS-Brew	Generated in the Peitz laboratory [52]
iLB-C89bf	hiPSC line generated from PBMC of a female donor (C89bf)		
iLB-C133bm UKBi013-A in hSPCreg	hiPSC line generated from PBMC of a male donor (C133bm)		
iLB-C16bm-2	hiPSC line generated from PBMC of a male donor (C16bm)		

### Differentiation of hiPSCs into glutamatergic cortical neurons

The cortical differentiation was performed as described before [53]. Specifically, iPSCs were cultured in mTeSR (StemCell Technologies) or StemMACS^™^ iPS-Brew (Miltenyi Biotec) and split with EDTA during maintenance culture. Undifferentiated iPSCs were dissociated with accutase and seeded as single cells at a density of 1 × 10^6^ cells per cm2 in iPSC medium with 10 μM ROCK inhibitor (RI) Y-27632 (Tocris). The next day, the medium was switched to GLUT neural induction medium (1:1 DMEMF12/N2:Neurobasal/B27, 1 μM Dorsomorphin/200 nM LDN-193189, 10 μM SB431542). On day 10, the neural induction medium was supplemented with 20 ng/ml FGF2 to accelerate neural rosette growth. On day 11, the cultures were split by incubating accutase for 15 min. Obtained cell clumps were seeded on Matrigel (MG)-coated 6-well plates in N2/B27 medium (1:1 DMEMF12/N2:Neurobasal/B27) with 20 ng/ml FGF2 and 10 μM RI using a split ratio of 1:3. On days 12 and 13, the medium was replaced with N2/B27 medium. From day 14 onward, cells were cultured in N2/B27 medium supplemented with 10 ng/ml FGF2 and 100 ng/ml heparin. On day 17 and day 22, the cultures were dissociated with accutase and seeded 1:2 on MG-coated plates for further propagation. On day 31, cortical neural precursor cultures were frozen down as one batch in ice-cold freezing medium (90% KOSR, 10% DMSO). Cortical neural precursors were thawed for further maturation and seeded in N2/B27 medium supplemented with 10 μM RI on MG-coated plates (0.5 Mio cells per cm^2^). On day 44, cultures were dissociated one more time and seeded for maturation. On the following day, the medium was replaced by N2/B27 medium with 10 μM PD0325901 and 10 μM DAPT to accelerate differentiation of persisting precursors. The medium was renewed on day 47. The cultures were mitotically inactivated with 5 μM AraC (Cytosine β-d-arabinofuranoside hydrochloride) on day 49. Up to the analysis cells were cultured further in N2/B27 medium with medium changes every other day, without aspirating the medium completely. After different time periods cells were processed for RNA isolation by guanidinium thiocyanate/phenol/chloroform extraction or protein isolation using the RIPA buffer as described [54, 55].

### DLD-1 cell culture, cytokine treatment and RNA and protein isolation

Human DLD-1 (ATCC, #CCL-221) cells were grown in DMEM with 10% inactivated fetal bovine serum, 2 mM l-glutamine, penicillin, and streptomycin. Eighteen hours before cytokine induction, the cells were washed with PBS and incubated with DMEM containing 2 mM l-glutamine in the absence of serum and phenol red. NOS2 expression in cells was induced with a cytokine mixture (CM) containing IFN-γ (100 U/ml), IL-1β (50 U/ml) and TNF-α (10 ng/ml) for the corresponding time periods depending on the experiment. Afterwards cells were processed for RNA isolation by guanidinium thiocyanate/phenol/chloroform extraction or protein isolation using the RIPA buffer as described [54, 55].

### Western blot experiments

To study the expression of NOS2- and GAPDH protein in DLD-1 or ILB-C89bf cells, total cellular protein (50–100 µg protein) was separated on SDS polyacrylamide gel and transferred to nitrocellulose membrane by semi-dry electroblotting. All further steps were performed as described previously [56]. For detection of NOS2 and GAPDH the antibodies listed in “Materials” section were used. The immunoreactive proteins on the blot were visualized by the enhanced chemiluminescence detection system (ECL, PerkinElmer, Rodgau, Germany) and processed using the ChemiDoc XRS+ system as described in the user manual (BioRad, Munich, Germany).

### Generation of DLD-1 cells with stable expression of a tetracycline-repressor (DLD-1_TR7)

DLD-1 cells were plated in normal medium onto 6 well plates and transfection with pcDNA6/TR by lipofection was performed with GeneJuice according to the manufacturer’s recommendations. 24 h after transfection the medium was changed and medium containing 10 µg/ml blasticidin was used for selection of cells. The cells were controlled microscopically each second day. After 14 days most cells had died, and only resistant cells survive. Then cells were transferred to small cell culture flasks and further incubated with cell medium containing blasticidin. After confluency the cells were frozen in liquid N_2_ and used for experiments.

### Transient transfection of DLD-1_TR7 cells, Griess assay, and Renilla-Luciferase reporter gene assay

DLD-1_TR7 cells were plated onto 24 well plates and transient transfection by lipofection was performed with GeneJuice according to the manufacturer’s recommendations. 0.3 µg of the plasmids containing the NOS2-1/2_cds_3UTR (pcDNA4/TO-NOS2-1_cds_3UTR and pcDNA4/TO-NOS2-2_cds_3UTR.) were combined with 0.1 µg of the renilla reporter gene plasmid pRL-EF-1α [50]. After 24 h incubation, cells were incubated 18 h in DMEM containing 2 mM l-glutamine in the absence of serum and phenol red. Afterwards cells were stimulated with 500 ng/ml tetracycline for 24 h. Then, the supernatants of the cells were removed for nitrate determination by the Griess assay, cells were lyzed in 1x Passive Lysis Buffer provided by the Dual-Luciferase-Reporter-Assay-System (Promega), and renilla luciferase activities were determined in 10 µl extracts. To analyze the nitrate levels in the supernatant a Griess assay was performed using the Griess reagent system from Promega as described by the manufacturer. The determined nitrate concentrations were normalized by the renilla luciferase light units after subtraction of extract background.

### Real-time reverse transcription polymerase chain reaction analysis

mRNA expression in DLD-1 cells or differentiating iPSC was quantified in a two-step real-time RT-PCR using either Taqman probes or SYBR Green as described before [57] with the oligonucleotides listed below.

**Table:** 

NOS2	
Sense	TGCAGACACGTGCGTTACTCC
Antisense	GGTAGCCAGCATAGCGGATG
Probe	TGGCAAGCACGACTTCCGGGTG
NOS2-1	
Sense	AGTCGAAAACTGAGGCTCCG
Antisense	TGCATCCAGCTTGACCAGAG
Probe	ACCCCGGGGAGGCAGTGCAGCCAGC
NOS2-2	
Sense	GCTCTGCAGGATCCTCCG
Antisense	GGGGACTCATTCTGCTGCTT
Probe	GCCGAAGCCTGACTGCTGCCCGCGC
18S rRNA	
Sense	CGGCTACCACATCCAAGGAA
Antisense	GCTGGAATTACCGCGGCT
GAPDH	
Sense	CCCATGTTCGTCATGGGTGT
Antisense	TGGTCATGAGTCCTTCCACGATA
Probe	CTGCACCACCAACTGCTTAGCACCC

Taqman hybridization probes were double labeled with 6-carboxyfluorescein (FAM) as reporter fluorophore and carboxytetramethyl rhodamine (TAMRA) as quencher. Fluorescence was monitored at each 60 °C step.

To calculate the relative expression of NOS2 mRNA in DLD-1 cells or iPSC the 2^−(ΔΔC(T))^ method [58] was used. According to this method the C(T) values for NOS2 mRNA expression in each sample were normalized to the C(T) values of 18S rRNA or GAPDH mRNA in the same sample. Then the values of untreated cell samples were set 100% and the percentage of NOS2-expression was calculated.

In case of the NOS2-isoform specific qRT-PCR analyses the RT reaction was performed with the gene-specific primer RT-rev (5′-TTGATCCTCACATGCCGTGG-3′).

### Statistics

Data represent means ± SEM. Statistical differences were determined by factorial analysis of variance followed by "Dunnett's" or “Bonferroni's” multiple comparison test. In the case of two means classical t-test analyses were used. All statistical analyses were performed using Graphpad Prism 9.

## Results

### Structure of the human NOS2 gene and the encoded mRNA isoforms

The human NOS2 gene is located on chromosome 17 position 27,756,766 to 27,800,529 on the complementary strand (GRCh38.p13; NC_000017.11). As shown in Additional file 1: Figs. S3 and S4, this gene contains 27 exons and 26 introns. Several of the exons (E1, E2, E1-diff, E8, E9) are alternatively used in the three different NOS2 transcripts (NOS2-1 to -3) described. The NOS2-1 mRNA (Additional file 1: Fig. S4) contains a 5′-untranslated region (5′-UTR) encoded by exon 1 and the 5′-part of exon 2, a coding sequence (cds) encoded by the 3′-part of exon 2 up to the 5′-part of exon 27, and a 3′-untranslated region (3′-UTR) encoded by the 3′-part of exon 27. In the 5′-UTR of the NOS2-1 mRNA an upstream open reading frame (µORF) is found, known to regulate NOS2-1 expression by the nonsense-mediated mRNA decay (NMD) mechanism [59]. The NOS2-1 protein (Additional file 1: Fig. S4) contains (amino- to carboxy-terminal) the DINNN motif important for the proteasomal degradation [60], an oxygenase domain, a zinc binding site, a calmodulin binding site, a reductase domain, a FMN binding site, two FAD binding sites, and two NADP binding sites. The NOS2-2 mRNA (Additional file 1: Fig. S4) lacks exon 1 and 2 and contains an alternative exon1-diff. The translation of this mRNA results in an NOS2-2 protein with a different amino-terminal sequence without a DINNN motif.

Analysis of the data in the human protein atlas (https://www.proteinatlas.org/) shows a significant expression of NOS2 in colon, rectum, small intestine, and placenta (trophoblast cells; see Additional file 1: Fig. S1). To analyze NOS2 mRNA expression in more detail we searched the SRA database (https://www.ncbi.nlm.nih.gov/sra) and Bioproject database (https://www.ncbi.nlm.nih.gov/bioproject/) for RNA-Seq data of human colon tissue samples. Using the CLC genomic workbench program (21.04) we analyzed the imported NGS data for NOS2 mRNA isoform expression.

### Expression of the NOS2-1 mRNA isoform in colon and cytokine-induced islets

**Fig. 1:**
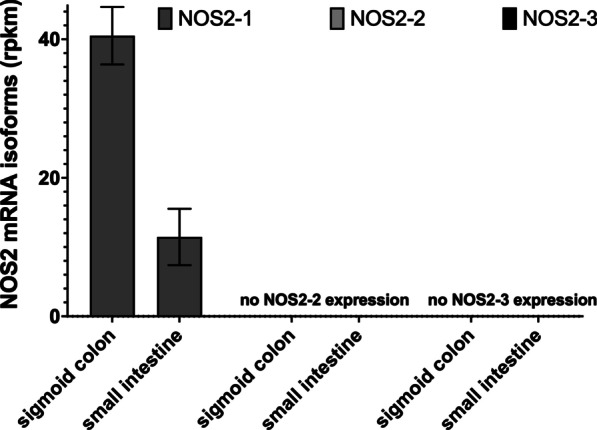
NOS2 mRNA isoform expression in the human sigmoid colon and small intestine. RNA-Seq data (gene expression omnibus GSM1010942, GSM1010940) were analyzed for human NOS2 mRNA isoform expression. Shown are the mean ± SEM of the rpkm values of the different NOS2 mRNA isoforms (NOS2-1, -2 and -3). There was no expression of NOS2-2 and -3

As shown in Fig. 1, only the mRNA isoform NOS2-1 (“immuno-NOS2”) was expressed in the human sigmoid colon and small intestine tissues (GSM1010942 and GSM1010940).

**Fig. 2:**
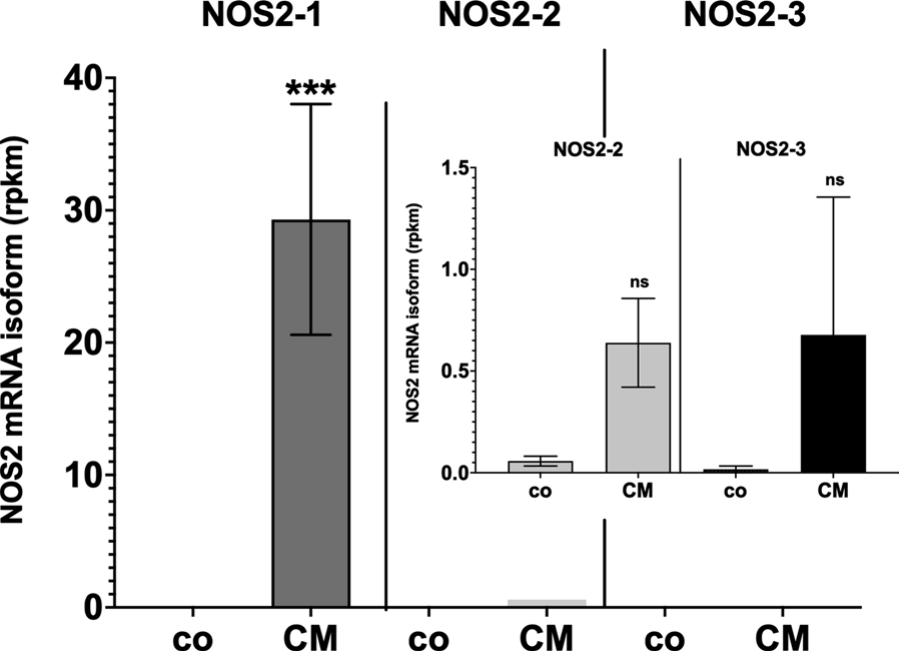
NOS2 mRNA isoform expression in isolated human islets treated with or without a cytokine mixture. RNA-Seq data (Bioproject PRJNA151601 [61]) were analyzed for human NOS2 mRNA isoform expression. Shown are the mean ± SEM of the rpkm values of the different NOS2 mRNA isoforms (NOS2-1, -2 and -3) expressed in isolated human islets incubated with (CM) or without a cytokine mixture (co) containing IL-1β and IFN-γ. ***FDR *p* value < 0.001, ns FDR *p* value > 0.05 versus co

To analyze the cytokine-induced NOS2 mRNA isoform expression in human tissues we analyzed the RNA-Seq data of Bioproject PRJNA151601 [61] for NOS2 expression. These data are obtained from isolated human islets incubated with or without a cytokine mixture (IL-1β and IFN-γ). As shown in Fig. 2, NOS2-1 showed the highest expression after cytokine induction. Although not significant compared to untreated islets, also a minor induction of NOS2-2 and NOS2-3 mRNA expression was detectable.

### The NOS2-2 mRNA isoform is expressed during the differentiation of hESC or hiPSC to different target cells

**Fig. 3:**
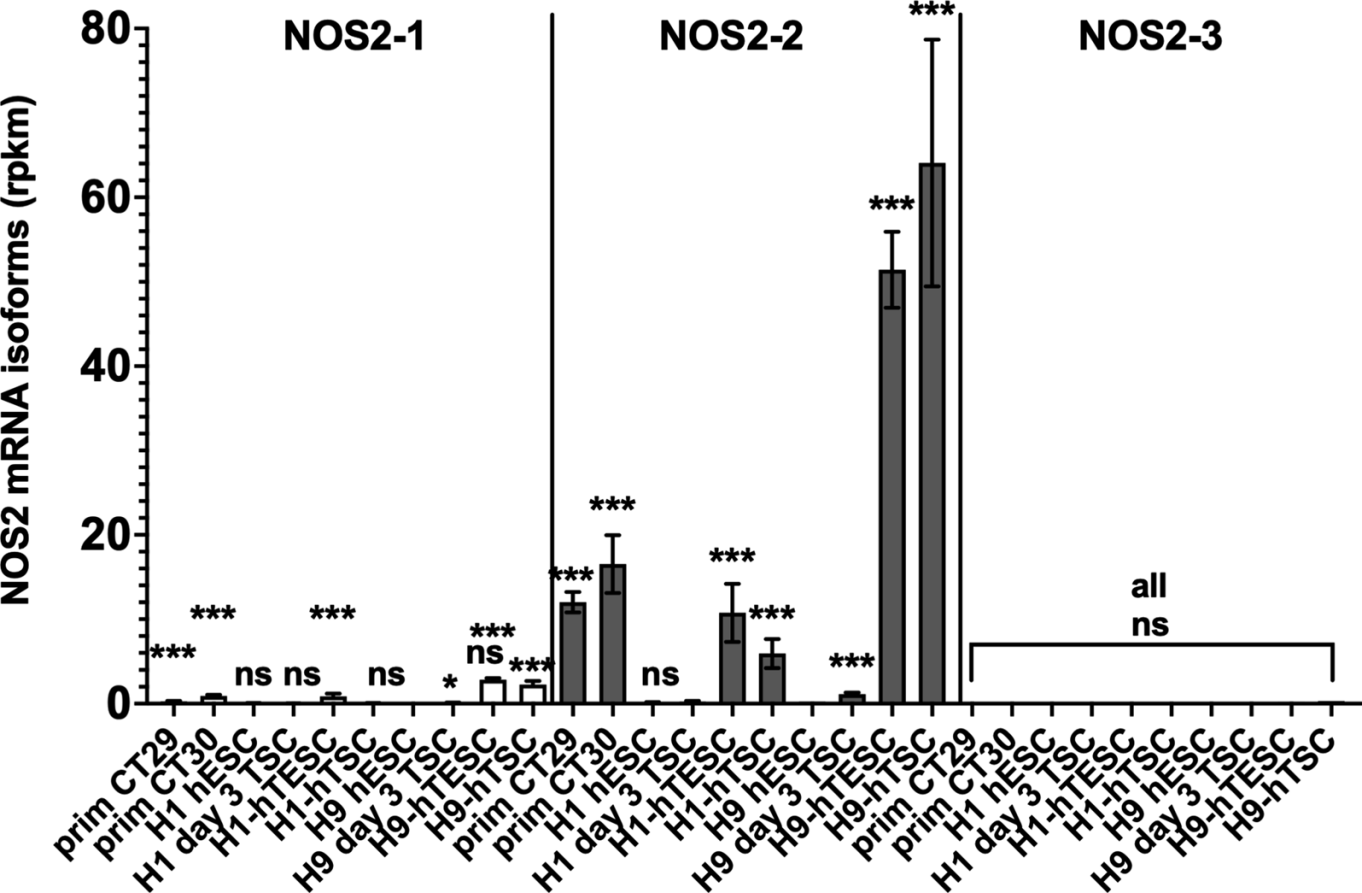
NOS2 mRNA isoform expression in different placenta-derived trophoblast stem cells (CT29, CT30) and H1- or H9-ESC induced to differentiate to trophoblast stem cells. RNA-Seq data (Bioproject PRJNA565033 [62] were analyzed for human NOS2 mRNA isoform expression. Shown are the mean ± SEM of the rpkm values of NOS2 mRNA isoforms at different differentiation stages. ***FDR *p* value < 0.001, ns FDR-value > 0.05 versus untreated H9-ESC

As stated above, analysis of NOS2 expression in the human protein atlas (https://www.proteinatlas.org/) shows a significant expression of NOS2 in trophoblast cells isolated from human placenta (Additional file 1: Fig. S1). To analyze NOS2 mRNA isoform expression, we searched for SRA datasets from human trophoblasts cells. The RNA-seq data published by Mischler et al. [62] compare the transcriptomes of trophoblast stem cells (TSC) CT29 and CT30 isolated from human placenta with TSC differentiated from hESC (H1- and H9-ESC). As shown in Fig. 3, mainly the expression of the NOS2 isoform NOS2-2 was detected by our analyses. Highest NOS2-2 mRNA expression was seen in H9-derived TSC without (H9-hTSC) or with CD20 expression (H9-hTESC). Also, our bioinformatic analysis of the RNA-Seq data published by Yabe et al. [63] revealed marked NOS2-2 mRNA expression in syncytiotrophoblast cells derived from H1-ESC (see Additional file 1: Fig. S5).

To further characterize NOS2 mRNA isoform expression in stem cell differentiation, we searched SRA databases for RNA-Seq experiments analyzing the transcriptomes of human ESC or iPSC during differentiation (longitudinal or time course analyses) to different cell types.

The RNA-Seq data of Bioproject PRNJA338181 [64] describe the transcriptomic analysis of human H1- and H9-ESC and C15- or C20-iPSC induced to differentiate to cardiomyocytes. As shown in Additional file 1: Fig. S6, in H9-ESC and C15-iPSC a significant enhanced marked expression of the NOS2-2 mRNA was seen transiently at day 4 of the differentiation process compared to the untreated cells. NOS2-1 and -3 mRNA expression was not significantly different. Interestingly, no such expression was seen in H1-ESC or C20-iPSC. Similar results were observed in our bioinformatic analysis of the RNA-Seq data published by Zhang et al. [65] (Bioproject PRJNA674506, see Additional file 1: Fig. S7) describing the time course of transcriptomes of human CD34-iPSC [66] induced to differentiate to cardiomyocytes.

Wu et al. [67] (Bioproject PRJNA674506) analyzed the transcriptomes of three different human iPSC lines (ATCC, BJFF and STAN) induced to develop to chondrocytes at different time points during the differentiation process. Our bioinformatic analyses (see Additional file 1: Fig. S8) showed a significant induction of the NOS2-2 mRNA isoform expression (compared to the hiPSC at day 0) only in ATCC cells in chondroprogenitor cells (CP) at day 7.

Luo et al. [68] (CNP0000771) published data analyzing the transcriptome profiles of hiPSC (developed from patient fibroblasts) and H9-ESC induced to differentiate to mesenchymal stromal cell (MSC) at different time points of the differentiation process. Our analyses (see Additional file 1: Fig. S9) regarding the NOS2 mRNA isoform expression showed a transient marked induction of the NOS2-2 mRNA expression in the H9-ESC (maximum at day 7). In the iPSC cells also an induction at day 7 was determined, but with a very low expression level.

**Fig. 4:**
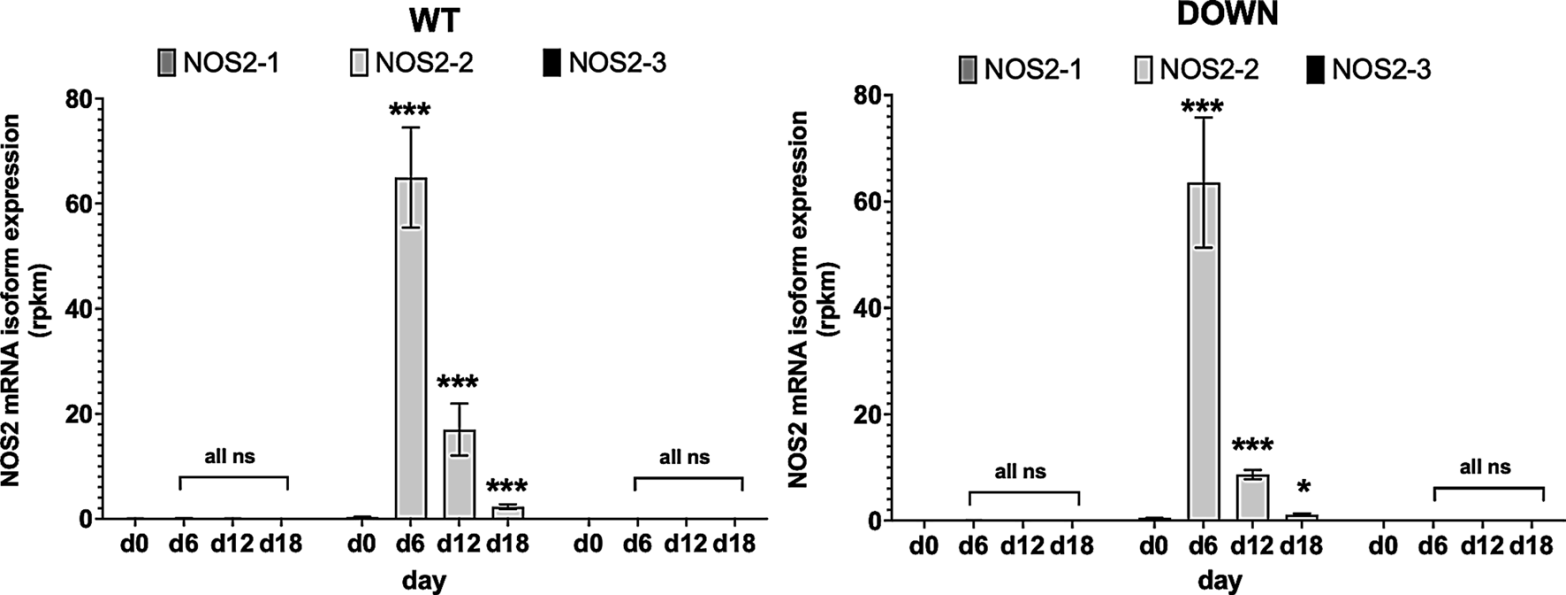
NOS2 mRNA isoform expression in human iPSC from normal (WT) and down syndrome (DOWN) donors induced to differentiate to neurons*.* RNA-Seq data (Bioproject PRJDB1099 [69]; FANTOM5) were analyzed for human NOS2 mRNA expression. Shown are the mean ± SEM of the rpkm values of the different NOS2 mRNA isoforms (NOS2-1, -2 and -3) at different time points (day 0 to day 18). ***FDR *p* value < 0.001, *FDR *p* value < 0.05, ns FDR *p* value > 0.05 versus day 0

Several RNA-Seq data found in the databases are related to analyses of transcriptomes of hESC or hiPSC induced to differentiate to different types of neurons. In Bioproject PRJDB1099 [69] (FANTOM5) a longitudinal analysis of transcriptomes in iPSC developed from normal probands (WT) and patient with trisomy 21 (Down-Syndrome, DOWN) is presented. These WT- or DOWN-iPSC were induced to differentiate to neurons. The RNA-Seq data were generated with a cap analysis of gene expression (CAGE) method. Therefore, only CAP-containing mRNAs were detected, and therefore the transcriptional start sites (TSS) could be mapped. As shown in Fig. 4, regardless of whether WT- or DOWN iPSC were analyzed only the expression of the NOS2-2 mRNA isoform was transiently induced (maximum at day 6). To support the data resulting from the CLC genomic workbench transcript mapping algorithm, we used the blast tool to map the RNA-Seq sequences of these CAGE-Seq-analyses to the whole human NOS2 gene (see Additional file 1: Fig. S10). This resulted in maximal hit numbers at the begin of the exon 1-diff genomic sequence supporting the result provided by the CLC genomic workbench transcript mapping algorithms.

Analyzing several other Bioproject dataset (Bioproject PRJNA244622, PRJNA404971, PRJNA433877, PRJNA59633, see Additional file 1: Figs. S11 to S14) using different protocols to H9-ESC or iPSC to differentiate to neurons for NOS2 mRNA isoform expression always resulted in similar results as presented above. In each case a significant transient induction only of the NOS2-2 mRNA at different time points in the differentiation process was seen.

**Fig. 5:**
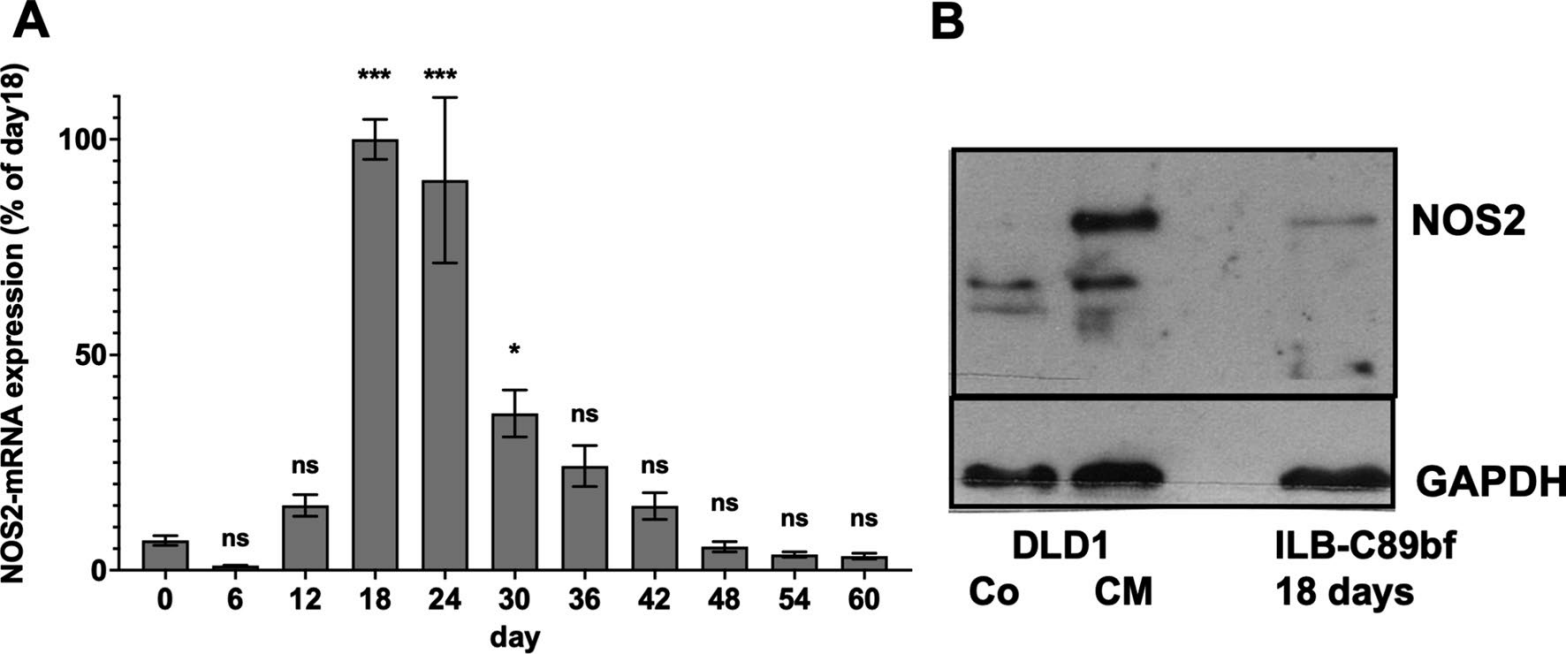
NOS2 mRNA- and protein expression in four different human iPSC lines induced to differentiate to neurons. Four different human iPSCs lines were generated from PBMC of three different donors [52]. RNA and protein were isolated from these 4 different human iPSC lines induced to differentiate to neurons [53] at different time points (day 0 to 60). **A** NOS2-mRNA and 18S rRNA expression were analyzed using the qRT-PCR method. NOS2 mRNA expression was normalized to the 18S rRNA expression. The relative NOS2 mRNA expression in the cells treated for 18 days was set to 100%. Shown is the summary of the analysis of the four different iPSC cell lines. The values represent the mean ± SEM of n = 12 different isolated RNAs at each time point. (***p < 0.001, *p < 0.05, ns not significant vs. iPSC treated for zero days; 1-way Anova with Dunnett's multiple comparisons test). **B** NOS2- and GAPDH protein expression in DLD1 cells (DLD1) incubated with (CM) or without (Co) a cytokine mixture to induce the human iNOS expression and ILB-C89bf (ILB-C89bf 18 days) cells stimulated to differentiate to neurons for 18 days were analyzed using the western blot method. Shown are the detected bands of NOS2- and GAPDH protein (analyzed using either the anti-NOS2- or the anti-GAPDH antibody)

**Fig. 6:**
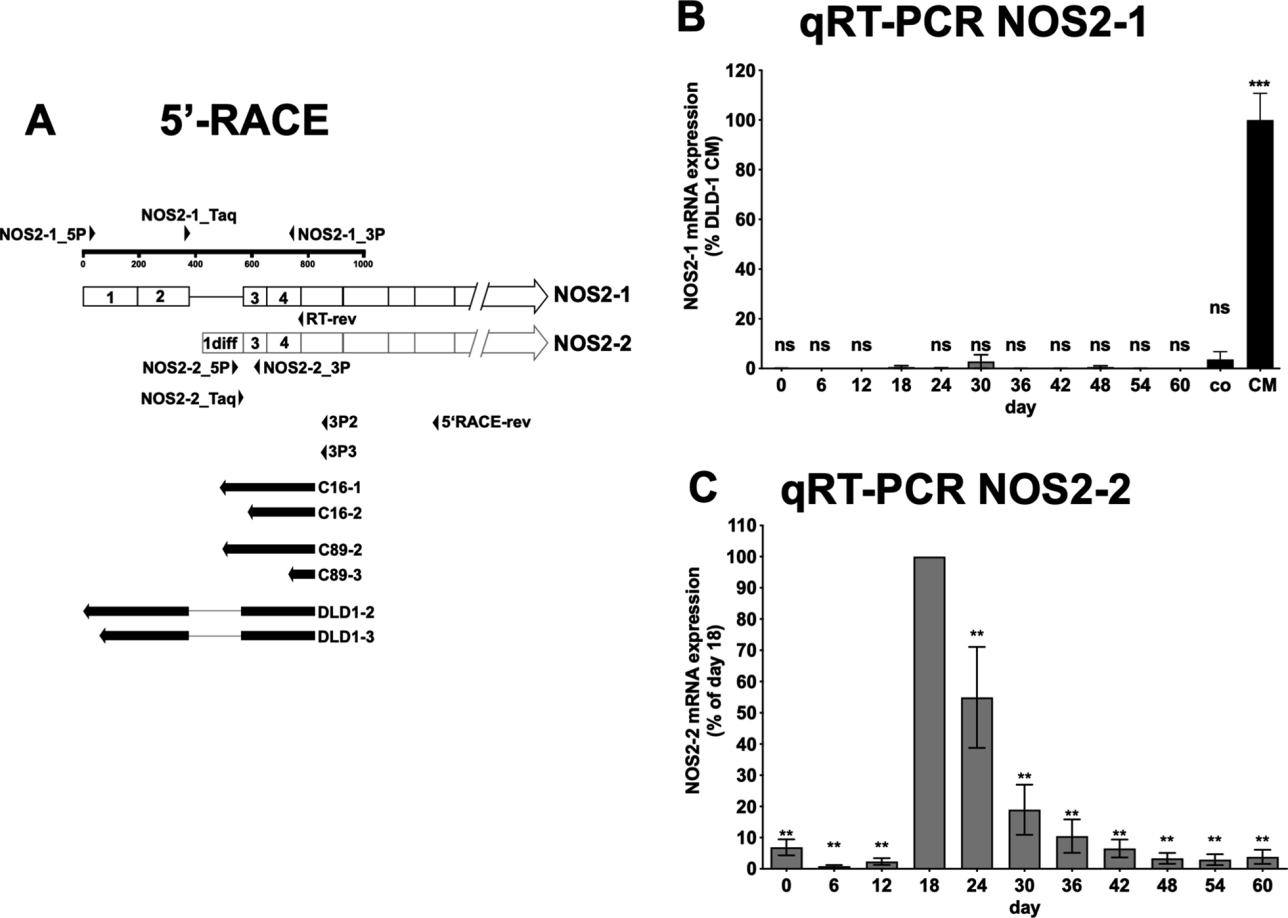
NOS2 mRNA isoform analysis using 5′RACE and isoform specific qRT-PCR methods. **A** Schematic summary of the results of 5′-RACE analyses using RNA isolated at day 18 from human iPSC induced to differentiate to neurons. RNA was isolated from 4 different human iPSC lines (samples C16-1, C16-2, C89-2, C89-3) induced to differentiate to neurons [53] for 18 days. As control RNA was isolated from human DLD1 cells (DLD1-2 and DLD1-2) treated with a cytokine mixture for 6 h to induce NOS2 mRNA expression. Then 5′-RACE experiments were performed (as described in “Methods” section) using the indicated oligonucleotides as 3′-primer (5′RACE-rev = primer used for the RT-reaction; 3P2/3P3 primers used for the PCR-reaction). The final PCR products were isolated, cloned into pCR-Script and sequenced. The alignment of these sequences to the human NOS2-1 and -2 mRNA are indicated as filled arrows. The sequences of the fragments are shown in Additional file 1: Fig. S10. **B/C** NOS2-mRNA-isoform expression analysis using isoform specific qRT-PCR experiments. RNA was isolated at different time points from 2 different human iPSC lines (iLB-C16bm s6 iPS andiLB-C89bf s4 iPCs) induced to differentiate to neurons [53] for 60 days. The RT reaction was performed with the RT-rev primer (see **A**). Taqman qPCR reactions were performed with mRNA isoform specific primer pairs and taqman probes (NOS2-1: NOS2-1_P, NOS2-1_3P and NOS2-1_Taq; NOS2-2: NOS2-2_5P, NOS2-2_3P and NOS2-2_Taq—see **A**). For normalization also the GAPDH mRNA expression was measured. **B** The normalized NOS2-1 mRNA expression values were related to the CM induced NOS2-1 mRNA expression in DLD-1 cells (CM = 100%). Also, the expression level of the NOS2-1 mRNA in untreated (co) DLD-1 cells were determined. (***p < 0.001, ns not significant vs. iPSC treated for 18 days; 1-way Anova with Dunnett’s multiple comparisons test). **C** The normalized NOS2-2 mRNA expression values were related to the NOS2-2 mRNA expression in iPSC treated for 18 days (day 18 = 100%). (**p < 0.05, vs. iPSC treated for 18 days; 1-way Anova with Dunnett’s multiple comparisons test)

To support the bioinformatic data, we differentiated four different hiPSC lines (iLB-C16bm, iLB-C16bm-2, iLB-C89bf and iLB-C133bm) towards glutaminergic neurons using an established protocol [53]. After different time periods (0 to 60 days) we isolated RNA or protein from the cells and measured NOS2 mRNA and 18S rRNA expression by qRT-PCR and protein expression by western blotting using monoclonal anti-NOS2- or GAPDH-antibodies. As shown in Fig. 5A (summary of all four iPSC lines) and Additional file 1: Fig. S15 (individual data of each cell line) we observed a transient induction of the NOS2 mRNA expression (maximal at day 18 and 24). Using protein extracts form iLB-C89bf cells induced for 18 days to differentiate to neurons we were also able to detect an NOS2 protein expression in these cells (Fig. 5B). As the primers used in the qRT-PCR and the monoclonal anti-NOS2 antibody used in the western blot analyses do not discriminate between the different NOS2 mRNA isoforms, we performed 5′-RACE experiments with the RNA isolated from the iPSCs at day 18 and cytokine induced DLD-1 cells. Compared to DLD-1 cells, which expressed the NOS2-1 isoform containing exon 1 and 2, the iPSC expressed the NOS2-2 mRNA with replacement of exon 1 and 2 by exon-1-diff (Fig. 6A). In addition, we analyzed the NOS2 mRNA isoform expression by performing NOS2-1/2 isoform specific qRT-PCR experiments. As shown in Fig. 6B, C, also in these analyses only the expression of the NOS2-2 mRNA was seen in the hiPSC lines induced to differentiate into neurons.

### The NOS2-2 protein is functional

**Fig. 7:**
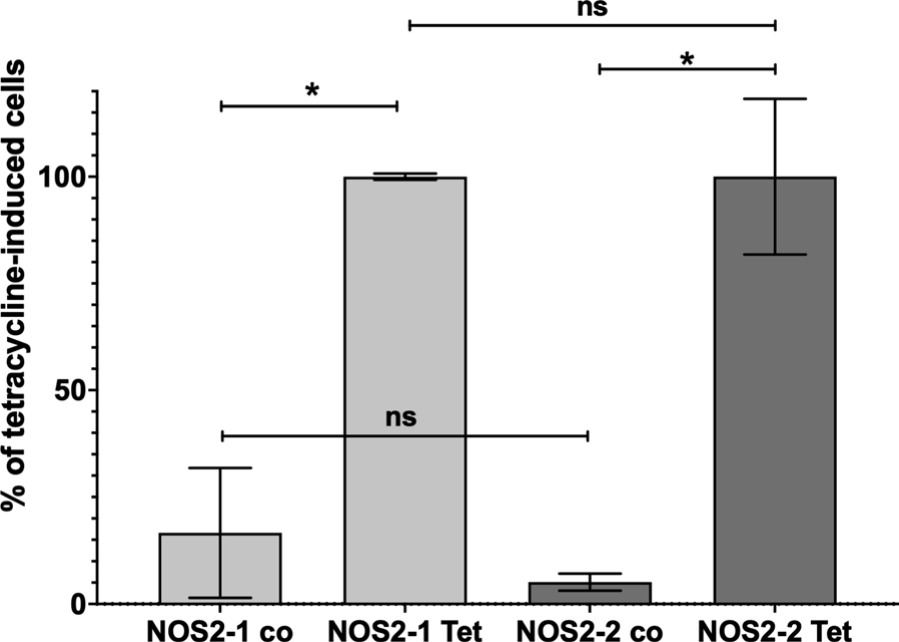
The NOS2-2 protein is functional. DLD-1_TR7 cells stably expressing a tetracycline repressor were transiently transfected with pcDNA4/TO_NOS2-1_cds_3UTR or pcDNA4/TO_NOS2-2_cds_3UTR encoding the NOS2-1 or -2 protein. To normalize the transfection efficiency, pRL-EF1α (encoding for a renilla luciferase) was cotransfected as well. After transfection, the cells were incubated with (Tet) or without (co) 500 ng/ml tetracycline for 24 h. Then the supernatants of the cells were used for nitrate concentration determination by the Griess assay. The cells were lyzed and renilla luciferase activity was measured. The nitrate concentrations determined were normalized to the renilla luciferase data. Shown is the summary of the analysis of the four different transfection experiments. The values represent the mean ± SEM of n = 8 different wells, (***p < 0.001; *p < 0.05, ns not significant;1-way Anova with Dunnett's multiple comparisons test)

Finally, to prove the functionality of the NOS2-2 protein, we performed transient transfection experiments using DLD-1_TR7 cells. As constitutively expression of NOS2 proteins is cytotoxic (Pautz et al., unpublished), we used the Tet-ON system. The DLD-1_TR7 cell express a tetracycline repressor (TR). pcDNA4/TO contains a CMV promoter driving the expression of inserted fragments containing binding sites for TR (TetO). In absence of tetracycline the promoter activity of the CMV promoter is blocked in DLD-1_TR7 cells. DLD-1_TR7 cells were transiently transfected with the constructs pcDNA4/TO_NOS2-1_cds_3UTR or pcDNA4/TO_NOS2-2_cds_3UTR encoding the NOS2-1 or -2 protein. To normalize the transfection efficiency, pRL-EF1α (encoding for a renilla luciferase) was cotransfected as well. After transfection, the cells were incubated with 500 ng/ml tetracycline for 24 h. Then the supernatants of the cells were used for nitrate concentration determination by the Griess assay. The cells were lyzed and renilla luciferase activity was measured. The nitrate concentrations determined were normalized to the renilla luciferase data and the data of tetracycline-induced cells were set to 100%. As shown in Fig. 7, both in the supernatant of NOS2-1- or NOS2-2-cds-3′-UTR transfected cells a similar enhancement of nitrate production was seen after tetracycline induction.

## Discussion

In human regenerative medicine SC therapy can entitled as the ultimate treatment of diseases or injury. hiPSC generated from easily obtained cells like fibroblasts or PBMC of the patient are an excellent tool for SC based therapies [70]. To fulfill these promises given by the SC-therapy the intracellular pathways important for the generation of hiPSC and the differentiation into the target cells/organs must be elucidated in detail. In the last years research in this field showed that NO displays an important role in the modulation of SC behavior. This includes regulation of cell survival, migration, differentiation, and paracrine secretion of proregenerative factors [5]. Low concentrations of NO seem to favor SC renewal [22] whereas higher concentrations induce differentiation by repression of the transcription factor Nanog [23]. About the nature of the NO producing enzyme in SC only limited information exist, but there is evidence that NOS2 plays an important role. As mentioned above, NOS2 is described as high NO output enzyme, whose expression is usually induced by inflammatory stimuli.

The description of the human NOS2 gene in the ENSEMBL database indicates the expression of three different NOS2 mRNA isoforms (NOS2-1 to -3; see Additional file 1: Figs. S3 and S4). The isoform NOS2-1 encodes for the “classical” cytokine-induced NOS2 enzyme. Based on the data of the human protein atlas NOS2 is significantly expressed in cells of different part of the colon (Additional file 1: Fig. S1). Analyzing public available RNA-seq data, we were able to reproduces these findings (Fig. 1). In addition, our analyses showed, that the NOS2 mRNA isoform NOS2-1 is the only isoform significantly expressed in these cells. As NOS2 is mainly believed to be expressed only after (cytokine)-induction of cells, this “constitutive” expression of NOS2-1 in the colon specimen is likely “induced” by the microbiota in this colon parts [71]. Data from cytokine induced isolated human islets also showed cytokine-induced expression of NOS2-1 mRNA (Fig. 2).

In addition to NOS2 expression in the different colon parts, the human protein atlas describes NOS2 expression in human placental cells. Analyzing RNA-Seq data from Mischler et al. [62] (comparing the transcriptomes of TSC isolated from the human placenta with TSC differentiated from hESC) and Yabe et al. [63] (transcriptomes from syncytiotrophoblast generated from H1-ESC) showed that in contrast to colon and islets, the primary isoform of the NOS2 mRNA expressed was the isoform NOS2-2 (Fig. 4 and Additional file 1: Fig. S5). These analyses also showed that the NOS2-2 mRNA expression was only transiently induced during the differentiation of the SC to the TSC. This finding was confirmed by further transcriptome analyses using different ESC or iPSC (see Additional file 1: Figs. S6–S9; S11–S14). Further, we have evidence that, the specific epigenetic background of the hiPSC line analyzed seems to determine whether an induction of the NOS2-2 mRNA occur (see Additional file 1: Figs. S6 and S8). In addition, the analyses of several other RNA-Seq data always resulted in similar results as presented above. In each case a significant transient induction only of the NOS2-2 mRNA at different time points in the differentiation process was seen. Also, Meng et al. described that in the (trans)-differentiation of human BJ- and murine primary fibroblasts to endothelial cells enhanced NOS2 expression and NOS2-related NO production is essentially involved [72]. However, the NOS2 mRNA isoform involved was not analyzed. Finally, we detected transient NOS2-2 mRNA and protein expression in four different hiPSC lines during their differentiation into glutamatergic neurons (see Figs. 5, 6). In summary, these data present evidence that NOS2-2 mRNA is the main isoform expressed during stem cell differentiation and that other isoforms as NOS2-1 or NOS2-3 mRNA are of minor importance in these processes. In contrast to NOS2-1 mRNA, where a lot of information about expressional regulation have been published, regulation of NOS2-2 mRNA expression is not known. Our data and sequence comparisons indicate that huge differences might exist. Compared to NOS2-1 mRNA, the NOS2-2 mRNA lacks exon 1 and 2 and contains an alternative exon1-diff. The RNA-Seq data in the study by Hon et al. [69] were generated with a CAGE method. This enables us, to use them to determine the TSS of the NOS2-2 transcripts in these cells. The blast analyses (against the whole human NOS2 gene) showed the highest number of 100% homologies hits at the begin of exon 1-diff (see Additional file 1: Fig. S10). So, it seems very likely, that the NOS2-2 transcript is generated by using a different promoter and not by alternative splicing. Also for the human NOS1 gene alternative promoter usage to generate cell specific NOS1 mRNA isoforms has been described [50, 73–75]. It is reasonable to speculate that the genomic region upstream of exon 1-diff contains the promoter sequences driving the expression of the NOS2-2 mRNA in differentiating cells. A bioinformatic analysis of all TF able to bind to the human NOS2 gene sequence (identified by ChIP analyses [76]) between exon 2 and exon 1-diff is shown in Additional file 1: Fig. S17 (description of the TF found in Additional file 1: Table S1). We also compared all RNA-seq data showing a significant enhanced NOS2-2 mRNA expression (PRJNA565303, PRJNA565303, CNP0000771, PRJDB1099_Down, PRJDB1099_WT, PRJNA244622, PRJNA338181_C15, PRJNA338181_H9, PRJNA674506, PRJNA59633) for the significant upregulation or downregulation (in the same direction in all data sets) of other transcripts. As shown in Additional file 1: Table S2, we detected 102 additional transcripts. TF which are shown to bind to the putative NOS2-2 promoter sequence and showed significant up- or downregulation in Additional file 1: Table S2 were highlighted in Additional file 1: Fig. S17. Future experiments must determine which of the TF(s) described are involved in the alternative promoter usage in differentiating hESC or hiPSC. One striking difference between NOS2-1 mRNA and NOS2-2 mRNA is the inducibility of mRNA expression mediated by cytokines/pro-inflammatory stimuli. Whereas NOS-2-1 mRNA expression largely depend on cytokine stimulation, NOS2-2 mRNA expression in differentiation processes seems to be inflammation-independent. One reason for that might be the different promotor structure and 5′-UTR sequence present in NOS2-2 mRNA. Despite differences in the N-terminal protein sequence between NOS2-1 and NOS2-2, the NOS2-2 mRNA derived NOS enzyme seems to produce similar amounts of NO as NOS2-1 protein (see Fig. 7).

In contrast to NOS2-1 protein, NOS2-2 enzyme lacks the DINNN-motif, which is important for the proteasomal degradation of NOS2-1 protein. In which way this difference is important for differential expression of NOS2-2 protein in SC remains to be elucidated. In addition, the signaling mechanism responsible for the transient NOS2-2 expression during SC differentiation processes must be investigated in future experiments to understand the importance of NOS2-2 in this field.

## Conclusions

In summary our bioinformatic analyses revealed transient NOS2-2 mRNA expression in hESC and hiPSC induced to differentiate into cardiomyocytes, chondrocytes, MSC, neurons, and trophoblast cells. In several analyses opposing results regarding the NOS-2–2 mRNA expression were obtained in hESC or hiPSC treated in parallel. This highlights the mention of Scesa et al. [77] that the different epigenetic background of the different ESC and iPSC seems to be important for the behavior of the differentiated cells obtained. In future, it would be interesting to know which epigenetic modifications are responsible for the observed phenomenon. It seems very likely, that the different epigenetic background of the hESC or hiPSC used determine if the NOS2-2 mRNA is expressed or not. Additional experiment in future must determine whether the NOS2-2 expression is important for differentiation and the functionality of the target cell type generated.

This study also demonstrates that in depth analyses of public available databases has a great potential to identify new signaling molecules important for biologicals processes where the availability of material, as often happens in the field of SC research, is a limiting factor.

## Supplementary Information

**Additional file 1.** Supplementary Data.

## Data Availability

All RNA-seq data used are publicly available (see “Methods” section). All data generated in this study are presented in the manuscript or in the Additional data.

## Abbreviations

5′-RACE: Rapid amplification of 5′mRNA ends
BM: Bone marrow
c-MYC: Cellular myelocytomatosis oncogene
CAGE: Cap analysis gene expression
cds: Coding sequence
CMV: Cytomegalo virus
CSC: Cardiac stem/progenitor cells
CYGB: Cytoglobin
DAPT: Tert-butyl (2S)-2-[[(2S)-2-[[2-(3,5-difluorophenyl)-acetyl]amino]propanoyl]amino]-2-phenylacetate
DCX: Doublecortin
dNTP: Deoxyribonucleotide triphosphate
EC: Endothelial cell
eNOS = NOS3: Endothelial nitric oxide synthase
EPC: Endothelial progenitor cell
FCS: Fetal calf serum
FGF-2: Fibroblast growth-factor 2
hESC: Human embryonic stem cell
hiPSC: Human induced pluripotent stem cell
hPSC: Human pluripotent stem cell
IL-1β: Interleukin-1β
INF-γ: Interferon-γ
iNOS = NOS2: Inducible nitric oxide synthase
KLF4: Kruppel Like Factor 4—KLF4
LIF: Leukemia inhibitory factor
LPS: Lipopolysaccharide
mRNA: Messenger RNA
MCC: Mesenchymal stem cell
Nanog: NANOG derives from Tír na nÓg (Irish for "Land of the Young")
ncRNA: Non-coding RNA
NF-κB: Nuclear factor κB
NMR: Nonsense-mediated mRNA decay
nNOS = NOS1: Neuronal nitric oxide synthase
µORF: Upstream open reading frame
NO: Nitric oxide
NOS2-1: NOS2 isoform induced by cytokines
NOS2-2: NOS2 isoform detected in differentiating hPSCs
NPC: Neuronal progenitor cells
NSC: Neuronal stem cells
OCT4: Octamer binding transcription factor 4
PCR: Polymerase chain reaction
qRT-PCR: Quantitative reverse transcriptase polymerase chain reaction
RNA-seq: RNA sequencing
SC: Stem cell
SDF-1: Stromal cell-derived factor-1 alpha
SEM: Standard error of mean
sGC: Soluble guanylyl cyclase
SOX2: Sex-determining region Y-box 2
STAT-1α: Signal transducer and activator of transcription-1α
Taq: Thermus aquaticus
TetO: Tetracycline operator—binding site for TR
TF: Transcription factor
TNF-α: Tumor necrosis factor-α
TR: Tetracycline repressor
TSC: Trophoblast stem cell
UTR: Untranslated region
WT: Wildtype
