# Supplementary material for: A specific, non-immune system-related isoform of the human inducible nitric oxide synthase is expressed during differentiation of human stem cells into various cell types
Source: Cell Commun Signal. 2022 Apr 7;20:47. doi: 10.1186/s12964-022-00855-x (PMC8991583; doi:10.1186/s12964-022-00855-x)
Supplement: Supplementary file 2 — Additional file 1. Supplementary Data. [file 12964_2022_855_MOESM2_ESM.pdf]

Supplemental tables

TF title	START (Ch17)	END (Ch17)	Start (G)	End (G)	Gene stable ID	Gene description
SMARCC1	27798640	27798684	1845	1889	ENSG00000173473	SWI/SNF related, matrix associated, actin dependent regulator of chromatin subfamily c member 1 [Source:HGNC Symbol;Acc:HGNC:11104]
ZBTB17	27798586	27798645	1884	1943	ENSG00000116809	zinc finger and BTB domain containing 17 [Source:HGNC Symbol;Acc:HGNC:12936]
KMT2B	27798586	27798693	1836	1943	ENSG00000272333	lysine methyltransferase 2B [Source:HGNC Symbol;Acc:HGNC:15840]
JUN	27798582	27798659	1870	1947	ENSG00000177606	Jun proto-oncogene, AP-1 transcription factor subunit [Source:HGNC Symbol;Acc:HGNC:6204]
NCOA1	27798580	27798665	1864	1949	ENSG00000084676	nuclear receptor coactivator 1 [Source:HGNC Symbol;Acc:HGNC:7668]
TEAD1	27798556	27798608	1921	1973	ENSG00000187079	TEA domain transcription factor 1 [Source:HGNC Symbol;Acc:HGNC:11714]
TP53	27798538	27798629	1900	1991	ENSG00000141510	tumor protein p53 [Source:HGNC Symbol;Acc:HGNC:11998]

CTCF	27798513	27798616	1913	2016	ENSG00000102974	CCCTC-binding factor [Source:HGNC Symbol;Acc:HGNC:13723]
TEAD4	27798513	27798588	1941	2016	ENSG00000197905	TEA domain transcription factor 4 [Source:HGNC Symbol;Acc:HGNC:11717]
BRD4	27798460	27798567	1962	2069	ENSG00000141867	bromodomain containing 4 [Source:HGNC Symbol;Acc:HGNC:13575]
SP140	27798367	27798460	2069	2162	ENSG00000079263	SP140 nuclear body protein [Source:HGNC Symbol;Acc:HGNC:17133]
ESR1	27798354	27798439	2090	2175	ENSG00000091831	estrogen receptor 1 [Source:HGNC Symbol;Acc:HGNC:3467]
TP63	27798278	27798371	2158	2251	ENSG00000073282	tumor protein p63 [Source:HGNC Symbol;Acc:HGNC:15979]
USF2	27798252	27798321	2208	2277	ENSG00000105698	upstream transcription factor 2, c-fos interacting [Source:HGNC Symbol;Acc:HGNC:12594]
BHLHE40	27798221	27798276	2253	2308	ENSG00000134107	basic helix-loop-helix family member e40 [Source:HGNC Symbol;Acc:HGNC:1046]
BRD4	27798204	27798311	2218	2325	ENSG00000141867	bromodomain containing 4 [Source:HGNC Symbol;Acc:HGNC:13575]
MYC	27798192	27798281	2248	2337	ENSG00000136997	MYC proto-oncogene, bHLH transcription factor

						[Source:HGNC Symbol;Acc:HGNC:7553]
STAT1	27798168	27798225	2304	2361	ENSG00000115415	signal transducer and activator of transcription 1 [Source:HGNC Symbol;Acc:HGNC:11362]
TFAP4	27798143	27798222	2307	2386	ENSG00000090447	transcription factor AP-4 [Source:HGNC Symbol;Acc:HGNC:11745]
ETS1	27798139	27798154	2375	2390	ENSG00000134954	ETS proto-oncogene 1, transcription factor [Source:HGNC Symbol;Acc:HGNC:3488]
TP53	27798136	27798247	2282	2393	ENSG00000141510	tumor protein p53 [Source:HGNC Symbol;Acc:HGNC:11998]
AR	27798115	27798220	2309	2414	ENSG00000169083	androgen receptor [Source:HGNC Symbol;Acc:HGNC:644]
FOXA2	27798079	27798172	2357	2450	ENSG00000125798	forkhead box A2 [Source:HGNC Symbol;Acc:HGNC:5022]
ZNF83	27798074	27798173	2356	2455	ENSG00000167766	zinc finger protein 83 [Source:HGNC Symbol;Acc:HGNC:13158]
STAT1	27798066	27798123	2406	2463	ENSG00000115415	signal transducer and activator of transcription 1 [Source:HGNC Symbol;Acc:HGNC:11362]

AR	27798048	27798091	2438	2481	ENSG00000169083	androgen receptor [Source:HGNC Symbol;Acc:HGNC:644]
ESR1	27798042	27798084	2445	2487	ENSG00000091831	estrogen receptor 1 [Source:HGNC Symbol;Acc:HGNC:3467]
NFE2	27798042	27798057	2472	2487	ENSG00000123405	nuclear factor, erythroid 2 [Source:HGNC Symbol;Acc:HGNC:7780]
PPARG	27798042	27798141	2388	2487	ENSG00000132170	peroxisome proliferator activated receptor gamma [Source:HGNC Symbol;Acc:HGNC:9236]
STAT5A	27798036	27798115	2414	2493	ENSG00000126561	signal transducer and activator of transcription 5A [Source:HGNC Symbol;Acc:HGNC:11366]
FOXP1	27798020	27798101	2428	2509	ENSG00000114861	forkhead box P1 [Source:HGNC Symbol;Acc:HGNC:3823]
FOXA1	27797997	27798100	2429	2532	ENSG00000129514	forkhead box A1 [Source:HGNC Symbol;Acc:HGNC:5021]
CRY1	27797990	27798091	2438	2539	ENSG00000008405	cryptochrome circadian regulator 1 [Source:HGNC Symbol;Acc:HGNC:2384]
BRD4	27797988	27798095	2434	2541	ENSG00000141867	bromodomain containing 4 [Source:HGNC Symbol;Acc:HGNC:13575]

PGR	27797973	27798070	2459	2556	ENSG00000082175	progesterone receptor [Source:HGNC Symbol;Acc:HGNC:8910]
FXR2	27797959	27797984	2545	2570	ENSG00000129245	FMR1 autosomal homolog 2 [Source:HGNC Symbol;Acc:HGNC:4024]
INTS11	27797957	27798044	2485	2572	ENSG00000127054	integrator complex subunit 11 [Source:HGNC Symbol;Acc:HGNC:26052]
HNF4A	27797951	27798033	2496	2578	ENSG00000101076	hepatocyte nuclear factor 4 alpha [Source:HGNC Symbol;Acc:HGNC:5024]
PDX1	27797949	27798012	2517	2580	ENSG00000139515	pancreatic and duodenal homeobox 1 [Source:HGNC Symbol;Acc:HGNC:6107]
TCF7L2/LEF1	27797947	27797985	2544	2582	ENSG00000148737	transcription factor 7 like 2 [Source:HGNC Symbol;Acc:HGNC:11641]
GATA4	27797942	27797975	2554	2587	ENSG00000285109	GATA binding protein 4 [Source:HGNC Symbol;Acc:HGNC:4173]
TCF7/LEF1	27797937	27797992	2537	2592	ENSG00000081059	transcription factor 7 [Source:HGNC Symbol;Acc:HGNC:11639]
TRPS1	27797934	27798025	2504	2595	ENSG00000104447	transcriptional repressor GATA binding 1 [Source:HGNC Symbol;Acc:HGNC:12340]

MEIS2	27797931	27797952	2577	2598	ENSG00000134138	Meis homeobox 2 [Source:HGNC Symbol;Acc:HGNC:7001]
ESR1	27797929	27798034	2495	2600	ENSG00000091831	estrogen receptor 1 [Source:HGNC Symbol;Acc:HGNC:3467]
GATA6	27797926	27797964	2565	2603	ENSG00000141448	GATA binding protein 6 [Source:HGNC Symbol;Acc:HGNC:4174]
NCOR2	27797925	27797992	2537	2604	ENSG00000196498	nuclear receptor corepressor 2 [Source:HGNC Symbol;Acc:HGNC:7673]
LHX9	27797923	27797970	2559	2606	ENSG00000143355	LIM homeobox 9 [Source:HGNC Symbol;Acc:HGNC:14222]
GATA2	27797922	27797999	2530	2607	ENSG00000179348	GATA binding protein 2 [Source:HGNC Symbol;Acc:HGNC:4171]
CEBPB	27797920	27797953	2576	2609	ENSG00000172216	CCAAT enhancer binding protein beta [Source:HGNC Symbol;Acc:HGNC:1834]
FOSL1	27797919	27797974	2555	2610	ENSG00000175592	FOS like 1, AP-1 transcription factor subunit [Source:HGNC Symbol;Acc:HGNC:13718]
NEUROD1	27797918	27797945	2584	2611	ENSG00000162992	neuronal differentiation 1 [Source:HGNC Symbol;Acc:HGNC:7762]

SMAD2	27797915	27797999	2530	2614	ENSG00000175387	SMAD family member 2 [Source:HGNC Symbol;Acc:HGNC:6768]
CTCF	27797912	27797961	2568	2617	ENSG00000102974	CCCTC-binding factor [Source:HGNC Symbol;Acc:HGNC:13723]
HOXB13	27797909	27797988	2541	2620	ENSG00000159184	homeobox B13 [Source:HGNC Symbol;Acc:HGNC:5112]
OTX2	27797908	27797940	2589	2621	ENSG00000165588	orthodenticle homeobox 2 [Source:HGNC Symbol;Acc:HGNC:8522]
ZBTB33	27797905	27797990	2539	2624	ENSG00000177485	zinc finger and BTB domain containing 33 [Source:HGNC Symbol;Acc:HGNC:16682]
TFAP4	27797904	27797969	2560	2625	ENSG00000090447	transcription factor AP-4 [Source:HGNC Symbol;Acc:HGNC:11745]
GATA3	27797901	27797976	2553	2628	ENSG00000107485	GATA binding protein 3 [Source:HGNC Symbol;Acc:HGNC:4172]
RCOR2	27797899	27797972	2557	2630	ENSG00000167771	REST corepressor 2 [Source:HGNC Symbol;Acc:HGNC:27455]
SP7	27797895	27797926	2603	2634	ENSG00000170374	Sp7 transcription factor [Source:HGNC Symbol;Acc:HGNC:17321]
AR	27797893	27797947	2582	2636	ENSG00000169083	androgen receptor [Source:HGNC Symbol;Acc:HGNC:644]

EP300	27797891	27797990	2539	2638	ENSG00000100393	E1A binding protein p300 [Source:HGNC Symbol;Acc:HGNC:3373]
PKNOX1	27797891	27797969	2560	2638	ENSG00000160199	PBX/knotted 1 homeobox 1 [Source:HGNC Symbol;Acc:HGNC:9022]
CDX2	27797890	27797913	2616	2639	ENSG00000165556	caudal type homeobox 2 [Source:HGNC Symbol;Acc:HGNC:1806]
GATAD2A	27797888	27797981	2548	2641	ENSG00000167491	GATA zinc finger domain containing 2A [Source:HGNC Symbol;Acc:HGNC:29989]
PBX4	27797885	27797910	2619	2644	ENSG00000105717	PBX homeobox 4 [Source:HGNC Symbol;Acc:HGNC:13403]
MIXL1	27797884	27797943	2586	2645	ENSG00000185155	Mix paired-like homeobox [Source:HGNC Symbol;Acc:HGNC:13363]
JUN	27797883	27797928	2601	2646	ENSG00000177606	Jun proto-oncogene, AP-1 transcription factor subunit [Source:HGNC Symbol;Acc:HGNC:6204]
BRD4	27797882	27797991	2538	2647	ENSG00000141867	bromodomain containing 4 [Source:HGNC Symbol;Acc:HGNC:13575]
BATF	27797880	27797931	2598	2649	ENSG00000156127	basic leucine zipper ATF-like transcription factor [Source:HGNC Symbol;Acc:HGNC:958]

TP53	27797875	27797944	2585	2654	ENSG00000141510	tumor protein p53 [Source:HGNC Symbol;Acc:HGNC:11998]
RELA	27797875	27797956	2573	2654	ENSG00000173039	RELA proto-oncogene, NF-kB subunit [Source:HGNC Symbol;Acc:HGNC:9955]
GRHL2	27797867	27797932	2597	2662	ENSG00000083307	grainyhead like transcription factor 2 [Source:HGNC Symbol;Acc:HGNC:2799]
SLC30A9	27797862	27797963	2566	2667	ENSG00000014824	solute carrier family 30 member 9 [Source:HGNC Symbol;Acc:HGNC:1329]
SAP130	27797859	27797960	2569	2670	ENSG00000136715	Sin3A associated protein 130 [Source:HGNC Symbol;Acc:HGNC:29813]
NCOA1	27797858	27797923	2606	2671	ENSG00000084676	nuclear receptor coactivator 1 [Source:HGNC Symbol;Acc:HGNC:7668]
ZBTB17	27797856	27797937	2592	2673	ENSG00000116809	zinc finger and BTB domain containing 17 [Source:HGNC Symbol;Acc:HGNC:12936]
SS18	27797854	27797947	2582	2675	ENSG00000141380	SS18 subunit of BAF chromatin remodeling complex [Source:HGNC Symbol;Acc:HGNC:11340]

ELF3	27797854	27797939	2590	2675	ENSG00000163435	E74 like ETS transcription factor 3 [Source:HGNC Symbol;Acc:HGNC:3318]
IRF1	27797852	27797931	2598	2677	ENSG00000125347	interferon regulatory factor 1 [Source:HGNC Symbol;Acc:HGNC:6116]
FOXA1	27797849	27797932	2597	2680	ENSG00000129514	forkhead box A1 [Source:HGNC Symbol;Acc:HGNC:5021]
SMAD3	27797846	27797933	2596	2683	ENSG00000166949	SMAD family member 3 [Source:HGNC Symbol;Acc:HGNC:6769]
FOXA2	27797831	27797918	2611	2698	ENSG00000125798	forkhead box A2 [Source:HGNC Symbol;Acc:HGNC:5022]
JUND	27797830	27797891	2638	2699	ENSG00000130522	JunD proto-oncogene, AP-1 transcription factor subunit [Source:HGNC Symbol;Acc:HGNC:6206]
KMT2B	27797823	27797922	2607	2706	ENSG00000272333	lysine methyltransferase 2B [Source:HGNC Symbol;Acc:HGNC:15840]
HNF4A	27797821	27797908	2621	2708	ENSG00000101076	hepatocyte nuclear factor 4 alpha [Source:HGNC Symbol;Acc:HGNC:5024]
JUNB	27797819	27797902	2627	2710	ENSG00000171223	JunB proto-oncogene, AP-1 transcription factor subunit [Source:HGNC Symbol;Acc:HGNC:6205]

SPI1	27797817	27797896	2633	2712	ENSG00000066336	Spi-1 proto-oncogene [Source:HGNC Symbol;Acc:HGNC:11241]
ONECUT2	27797815	27797890	2639	2714	ENSG00000119547	one cut homeobox 2 [Source:HGNC Symbol;Acc:HGNC:8139]
ZNF263	27797797	27797824	2705	2732	ENSG00000006194	zinc finger protein 263 [Source:HGNC Symbol;Acc:HGNC:13056]
REST	27797780	27797859	2670	2749	ENSG00000084093	RE1 silencing transcription factor [Source:HGNC Symbol;Acc:HGNC:9966]
TP63	27797759	27797852	2677	2770	ENSG00000073282	tumor protein p63 [Source:HGNC Symbol;Acc:HGNC:15979]
PARP1	27797756	27797849	2680	2773	ENSG00000143799	poly(ADP-ribose) polymerase 1 [Source:HGNC Symbol;Acc:HGNC:270]
SMARCA4	27797699	27797804	2725	2830	ENSG00000127616	SWI/SNF related, matrix associated, actin dependent regulator of chromatin, subfamily a, member 4 [Source:HGNC Symbol;Acc:HGNC:11100]
FOXA1	27797683	27797786	2743	2846	ENSG00000129514	forkhead box A1 [Source:HGNC Symbol;Acc:HGNC:5021]
CEBPB	27797680	27797716	2813	2849	ENSG00000172216	CCAAT enhancer binding protein beta

						[Source:HGNC Symbol;Acc:HGNC:1834]
ESR1	27797644	27797749	2780	2885	ENSG00000091831	estrogen receptor 1 [Source:HGNC Symbol;Acc:HGNC:3467]
STAG1	27797627	27797724	2805	2902	ENSG00000118007	stromal antigen 1 [Source:HGNC Symbol;Acc:HGNC:11354]
SRF	27797588	27797610	2919	2941	ENSG00000112658	serum response factor [Source:HGNC Symbol;Acc:HGNC:11291]
CTCF	27797580	27797605	2924	2949	ENSG00000102974	CCCTC-binding factor [Source:HGNC Symbol;Acc:HGNC:13723]
CUL4A	27797578	27797657	2872	2951	ENSG00000139842	cullin 4A [Source:HGNC Symbol;Acc:HGNC:2554]
RAD21	27797578	27797623	2906	2951	ENSG00000164754	RAD21 cohesin complex component [Source:HGNC Symbol;Acc:HGNC:9811]
SOX2	27797574	27797663	2866	2955	ENSG00000181449	SRY-box transcription factor 2 [Source:HGNC Symbol;Acc:HGNC:11195]
CTCFL	27797570	27797609	2920	2959	ENSG00000124092	CCCTC-binding factor like [Source:HGNC Symbol;Acc:HGNC:16234]
E2F1	27797567	27797662	2867	2962	ENSG00000101412	E2F transcription factor 1 [Source:HGNC Symbol;Acc:HGNC:3113]

KLF4	27797557	27797646	2883	2972	ENSG00000136826	Kruppel like factor 4 [Source:HGNC Symbol;Acc:HGNC:6348]
ESR1	27797555	27797660	2869	2974	ENSG00000091831	estrogen receptor 1 [Source:HGNC Symbol;Acc:HGNC:3467]
SMARCA4	27797535	27797640	2889	2994	ENSG00000127616	SWI/SNF related, matrix associated, actin dependent regulator of chromatin, subfamily a, member 4 [Source:HGNC Symbol;Acc:HGNC:11100]
SUZ12	27797510	27797611	2918	3019	ENSG00000178691	SUZ12 polycomb repressive complex 2 subunit [Source:HGNC Symbol;Acc:HGNC:17101]
FOXA1	27797506	27797609	2920	3023	ENSG00000129514	forkhead box A1 [Source:HGNC Symbol;Acc:HGNC:5021]
E2F6	27797498	27797574	2955	3031	ENSG00000169016	E2F transcription factor 6 [Source:HGNC Symbol;Acc:HGNC:3120]
ESR1	27797397	27797502	3027	3132	ENSG00000091831	estrogen receptor 1 [Source:HGNC Symbol;Acc:HGNC:3467]
SOX8	27797396	27797485	3044	3133	ENSG00000005513	SRY-box transcription factor 8 [Source:HGNC Symbol;Acc:HGNC:11203]
ESR1	27797271	27797376	3153	3258	ENSG00000091831	estrogen receptor 1 [Source:HGNC Symbol;Acc:HGNC:3467]

PARP1	27797247	27797340	3189	3282	ENSG00000143799	poly(ADP-ribose) polymerase 1 [Source:HGNC Symbol;Acc:HGNC:270]
CRY1	27797242	27797343	3186	3287	ENSG00000008405	cryptochrome circadian regulator 1 [Source:HGNC Symbol;Acc:HGNC:2384]
UBP1	27797215	27797316	3213	3314	ENSG00000153560	upstream binding protein 1 [Source:HGNC Symbol;Acc:HGNC:12507]
DTL	27797124	27797225	3304	3405	ENSG00000143476	denticleless E3 ubiquitin protein ligase homolog [Source:HGNC Symbol;Acc:HGNC:30288]
CTCF	27797082	27797185	3344	3447	ENSG00000102974	CCCTC-binding factor [Source:HGNC Symbol;Acc:HGNC:13723]
ESR1	27797023	27797128	3401	3506	ENSG00000091831	estrogen receptor 1 [Source:HGNC Symbol;Acc:HGNC:3467]
SUZ12	27797005	27797100	3429	3524	ENSG00000178691	SUZ12 polycomb repressive complex 2 subunit [Source:HGNC Symbol;Acc:HGNC:17101]
REST	27796992	27797089	3440	3537	ENSG00000084093	RE1 silencing transcription factor [Source:HGNC Symbol;Acc:HGNC:9966]
TP53	27796984	27797095	3434	3545	ENSG00000141510	tumor protein p53 [Source:HGNC Symbol;Acc:HGNC:11998]

FOXA1	27796979	27797082	3447	3550	ENSG00000129514	forkhead box A1 [Source:HGNC Symbol;Acc:HGNC:5021]
GLI1	27796956	27797015	3514	3573	ENSG00000111087	GLI family zinc finger 1 [Source:HGNC Symbol;Acc:HGNC:4317]
ESR1	27796943	27797048	3481	3586	ENSG00000091831	estrogen receptor 1 [Source:HGNC Symbol;Acc:HGNC:3467]
ZFP36	27796924	27796991	3538	3605	ENSG00000128016	ZFP36 ring finger protein [Source:HGNC Symbol;Acc:HGNC:12862]
BRD4	27796894	27796999	3530	3635	ENSG00000141867	bromodomain containing 4 [Source:HGNC Symbol;Acc:HGNC:13575]
ZNF16	27796893	27796994	3535	3636	ENSG00000170631	zinc finger protein 16 [Source:HGNC Symbol;Acc:HGNC:12947]
RUNX1	27796885	27796980	3549	3644	ENSG00000159216	RUNX family transcription factor 1 [Source:HGNC Symbol;Acc:HGNC:10471]
DTL	27796848	27796949	3580	3681	ENSG00000143476	denticleless E3 ubiquitin protein ligase homolog [Source:HGNC Symbol;Acc:HGNC:30288]
SIRT1	27796801	27796880	3649	3728	ENSG00000096717	sirtuin 1 [Source:HGNC Symbol;Acc:HGNC:14929]
AR	27796755	27796802	3727	3774	ENSG00000169083	androgen receptor [Source:HGNC Symbol;Acc:HGNC:644]

SUZ12	27796753	27796852	3677	3776	ENSG00000178691	SUZ12 polycomb repressive complex 2 subunit [Source:HGNC Symbol;Acc:HGNC:17101]
ESR1	27796720	27796779	3750	3809	ENSG00000091831	estrogen receptor 1 [Source:HGNC Symbol;Acc:HGNC:3467]
HDAC2	27796673	27796768	3761	3856	ENSG00000196591	histone deacetylase 2 [Source:HGNC Symbol;Acc:HGNC:4853]
BRD4	27796669	27796774	3755	3860	ENSG00000141867	bromodomain containing 4 [Source:HGNC Symbol;Acc:HGNC:13575]
SMARCA4	27796619	27796724	3805	3910	ENSG00000127616	SWI/SNF related, matrix associated, actin dependent regulator of chromatin, subfamily a, member 4 [Source:HGNC Symbol;Acc:HGNC:11100]
FLI1	27796583	27796684	3845	3946	ENSG00000151702	Fli-1 proto-oncogene, ETS transcription factor [Source:HGNC Symbol;Acc:HGNC:3749]
DTL	27796577	27796678	3851	3952	ENSG00000143476	denticleless E3 ubiquitin protein ligase homolog [Source:HGNC Symbol;Acc:HGNC:30288]
ESR1	27796576	27796681	3848	3953	ENSG00000091831	estrogen receptor 1 [Source:HGNC Symbol;Acc:HGNC:3467]

SUZ12	27796559	27796654	3875	3970	ENSG00000178691	SUZ12 polycomb repressive complex 2 subunit [Source:HGNC Symbol;Acc:HGNC:17101]
SOX8	27796410	27796499	4030	4119	ENSG00000005513	SRY-box transcription factor 8 [Source:HGNC Symbol;Acc:HGNC:11203]
CTCF	27796361	27796464	4065	4168	ENSG00000102974	CCCTC-binding factor [Source:HGNC Symbol;Acc:HGNC:13723]
NR3C1	27796315	27796414	4115	4214	ENSG00000113580	nuclear receptor subfamily 3 group C member 1 [Source:HGNC Symbol;Acc:HGNC:7978]
SUZ12	27796182	27796277	4252	4347	ENSG00000178691	SUZ12 polycomb repressive complex 2 subunit [Source:HGNC Symbol;Acc:HGNC:17101]
SFPQ	27796103	27796162	4367	4426	ENSG00000116560	splicing factor proline and glutamine rich [Source:HGNC Symbol;Acc:HGNC:10774]
ZNF121	27796023	27796098	4431	4506	ENSG00000197961	zinc finger protein 121 [Source:HGNC Symbol;Acc:HGNC:12904]
SPI1	27796006	27796119	4410	4523	ENSG00000066336	Spi-1 proto-oncogene [Source:HGNC Symbol;Acc:HGNC:11241]
TWIST1	27795976	27796057	4472	4553	ENSG00000122691	twist family bHLH transcription factor 1

						[Source:HGNC Symbol;Acc:HGNC:12428]
ELF1	27795928	27796017	4512	4601	ENSG00000120690	E74 like ETS transcription factor 1 [Source:HGNC Symbol;Acc:HGNC:3316]
PARP1	27795887	27795980	4549	4642	ENSG00000143799	poly(ADP-ribose) polymerase 1 [Source:HGNC Symbol;Acc:HGNC:270]
ARNT	27795770	27795853	4676	4759	ENSG00000143437	aryl hydrocarbon receptor nuclear translocator [Source:HGNC Symbol;Acc:HGNC:700]
CDK2	27795625	27795718	4811	4904	ENSG00000123374	cyclin dependent kinase 2 [Source:HGNC Symbol;Acc:HGNC:1771]
STAT1	27795539	27795622	4907	4990	ENSG00000115415	signal transducer and activator of transcription 1 [Source:HGNC Symbol;Acc:HGNC:11362]
FOXA2	27795501	27795594	4935	5028	ENSG00000125798	forkhead box A2 [Source:HGNC Symbol;Acc:HGNC:5022]
NR3C1	27795400	27795494	5035	5129	ENSG00000113580	nuclear receptor subfamily 3 group C member 1 [Source:HGNC Symbol;Acc:HGNC:7978]
GATA2	27795393	27795429	5100	5136	ENSG00000179348	GATA binding protein 2 [Source:HGNC Symbol;Acc:HGNC:4171]

ERF	27795376	27795412	5117	5153	ENSG00000105722	ETS2 repressor factor [Source:HGNC Symbol;Acc:HGNC:3444]
SMARCA4	27795376	27795481	5048	5153	ENSG00000127616	SWI/SNF related, matrix associated, actin dependent regulator of chromatin, subfamily a, member 4 [Source:HGNC Symbol;Acc:HGNC:11100]
FOXA1	27795375	27795394	5135	5154	ENSG00000129514	forkhead box A1 [Source:HGNC Symbol;Acc:HGNC:5021]
FOXA2	27795371	27795408	5121	5158	ENSG00000125798	forkhead box A2 [Source:HGNC Symbol;Acc:HGNC:5022]
SUMO2	27795370	27795412	5117	5159	ENSG00000188612	small ubiquitin like modifier 2 [Source:HGNC Symbol;Acc:HGNC:11125]
TLE3	27795369	27795393	5136	5160	ENSG00000140332	TLE family member 3, transcriptional corepressor [Source:HGNC Symbol;Acc:HGNC:11839]
BDP1	27795369	27795462	5067	5160	ENSG00000273873	B double prime 1, subunit of RNA polymerase III transcription initiation factor IIIB [Source:HGNC Symbol;Acc:HGNC:13652]
AR	27795367	27795397	5132	5162	ENSG00000169083	androgen receptor [Source:HGNC Symbol;Acc:HGNC:644]

NKX3-1	27795362	27795418	5111	5167	ENSG00000167034	NK3 homeobox 1 [Source:HGNC Symbol;Acc:HGNC:7838]
HOXB13	27795361	27795396	5133	5168	ENSG00000159184	homeobox B13 [Source:HGNC Symbol;Acc:HGNC:5112]
ONECUT2	27795353	27795390	5139	5176	ENSG00000119547	one cut homeobox 2 [Source:HGNC Symbol;Acc:HGNC:8139]
SPI1	27795343	27795456	5073	5186	ENSG00000066336	Spi-1 proto-oncogene [Source:HGNC Symbol;Acc:HGNC:11241]
MBL2	27795343	27795386	5143	5186	ENSG00000165471	mannose binding lectin 2 [Source:HGNC Symbol;Acc:HGNC:6922]
ARID1A	27795340	27795433	5096	5189	ENSG00000117713	AT-rich interaction domain 1A [Source:HGNC Symbol;Acc:HGNC:11110]
PIAS1	27795337	27795414	5115	5192	ENSG00000033800	protein inhibitor of activated STAT 1 [Source:HGNC Symbol;Acc:HGNC:2752]
ERG	27795315	27795393	5136	5214	ENSG00000157554	ETS transcription factor ERG [Source:HGNC Symbol;Acc:HGNC:3446]
ASCL2	27795250	27795331	5198	5279	ENSG00000183734	achaete-scute family bHLH transcription factor 2 [Source:HGNC Symbol;Acc:HGNC:739]

FOXA2	27795210	27795303	5226	5319	ENSG00000125798	forkhead box A2 [Source:HGNC Symbol;Acc:HGNC:5022]
PARP1	27795204	27795297	5232	5325	ENSG00000143799	poly(ADP-ribose) polymerase 1 [Source:HGNC Symbol;Acc:HGNC:270]
ZKSCAN1	27795185	27795299	5230	5344	ENSG00000106261	zinc finger with KRAB and SCAN domains 1 [Source:HGNC Symbol;Acc:HGNC:13101]
AHR	27795185	27795262	5267	5344	ENSG00000106546	aryl hydrocarbon receptor [Source:HGNC Symbol;Acc:HGNC:348]
ESR1	27795183	27795288	5241	5346	ENSG00000091831	estrogen receptor 1 [Source:HGNC Symbol;Acc:HGNC:3467]
NFKB1	27795172	27795253	5276	5357	ENSG00000109320	nuclear factor kappa B subunit 1 [Source:HGNC Symbol;Acc:HGNC:7794]
ZNF490	27795165	27795292	5237	5364	ENSG00000188033	zinc finger protein 490 [Source:HGNC Symbol;Acc:HGNC:23705]
CTCF	27795158	27795243	5286	5371	ENSG00000102974	CCCTC-binding factor [Source:HGNC Symbol;Acc:HGNC:13723]
ME1	27795143	27795215	5314	5386	ENSG00000065833	malic enzyme 1 [Source:HGNC Symbol;Acc:HGNC:6983]
ARNTL	27795135	27795218	5311	5394	ENSG00000133794	aryl hydrocarbon receptor nuclear translocator like

						[Source:HGNC Symbol;Acc:HGNC:701]
ZKSCAN1	27795108	27795163	5366	5421	ENSG00000106261	zinc finger with KRAB and SCAN domains 1 [Source:HGNC Symbol;Acc:HGNC:13101]
SPI1	27795104	27795165	5364	5425	ENSG00000066336	Spi-1 proto-oncogene [Source:HGNC Symbol;Acc:HGNC:11241]
BPTF	27795077	27795170	5359	5452	ENSG00000171634	bromodomain PHD finger transcription factor [Source:HGNC Symbol;Acc:HGNC:3581]
ESR1	27795067	27795146	5383	5462	ENSG00000091831	estrogen receptor 1 [Source:HGNC Symbol;Acc:HGNC:3467]
AR	27795030	27795135	5394	5499	ENSG00000169083	androgen receptor [Source:HGNC Symbol;Acc:HGNC:644]
ZFHX2	27795025	27795158	5371	5504	ENSG00000136367	zinc finger homeobox 2 [Source:HGNC Symbol;Acc:HGNC:20152]
CTCF	27795024	27795057	5472	5505	ENSG00000102974	CCCTC-binding factor [Source:HGNC Symbol;Acc:HGNC:13723]
GATA2	27795022	27795111	5418	5507	ENSG00000179348	GATA binding protein 2 [Source:HGNC Symbol;Acc:HGNC:4171]
RAD21	27795017	27795079	5450	5512	ENSG00000164754	RAD21 cohesin complex component

						[Source:HGNC Symbol;Acc:HGNC:9811]
EGR2	27795011	27795097	5432	5518	ENSG00000122877	early growth response 2 [Source:HGNC Symbol;Acc:HGNC:3239]
SP5	27794995	27795020	5509	5534	ENSG00000204335	Sp5 transcription factor [Source:HGNC Symbol;Acc:HGNC:14529]
ORC1	27794988	27795123	5406	5541	ENSG00000085840	origin recognition complex subunit 1 [Source:HGNC Symbol;Acc:HGNC:8487]
SUZ12	27794987	27795082	5447	5542	ENSG00000178691	SUZ12 polycomb repressive complex 2 subunit [Source:HGNC Symbol;Acc:HGNC:17101]
MAZ	27794974	27795065	5464	5555	ENSG00000103495	MYC associated zinc finger protein [Source:HGNC Symbol;Acc:HGNC:6914]
BMI1	27794974	27795059	5470	5555	ENSG00000168283	BMI1 proto-oncogene, polycomb ring finger [Source:HGNC Symbol;Acc:HGNC:1066]
KLF15	27794969	27795012	5517	5560	ENSG00000163884	Kruppel like factor 15 [Source:HGNC Symbol;Acc:HGNC:14536]
ZNF692	27794968	27794993	5536	5561	ENSG00000171163	zinc finger protein 692 [Source:HGNC Symbol;Acc:HGNC:26049]

HNF4A	27794963	27795040	5489	5566	ENSG00000101076	hepatocyte nuclear factor 4 alpha [Source:HGNC Symbol;Acc:HGNC:5024]
EGR3	27794960	27795087	5442	5569	ENSG00000179388	early growth response 3 [Source:HGNC Symbol;Acc:HGNC:3240]
ZNF143	27794929	27795024	5505	5600	ENSG00000166478	zinc finger protein 143 [Source:HGNC Symbol;Acc:HGNC:12928]
ZFP69B	27794921	27795022	5507	5608	ENSG00000187801	ZFP69 zinc finger protein B [Source:HGNC Symbol;Acc:HGNC:28053]
FOXA1	27794916	27795019	5510	5613	ENSG00000129514	forkhead box A1 [Source:HGNC Symbol;Acc:HGNC:5021]
ZNF467	27794891	27795042	5487	5638	ENSG00000181444	zinc finger protein 467 [Source:HGNC Symbol;Acc:HGNC:23154]
AR	27794793	27794898	5631	5736	ENSG00000169083	androgen receptor [Source:HGNC Symbol;Acc:HGNC:644]
ESR1	27794753	27794858	5671	5776	ENSG00000091831	estrogen receptor 1 [Source:HGNC Symbol;Acc:HGNC:3467]
ERG	27794704	27794799	5730	5825	ENSG00000157554	ETS transcription factor ERG [Source:HGNC Symbol;Acc:HGNC:3446]
FOXA1	27794699	27794728	5801	5830	ENSG00000129514	forkhead box A1 [Source:HGNC Symbol;Acc:HGNC:5021]

FOXA2	27794683	27794776	5753	5846	ENSG00000125798	forkhead box A2 [Source:HGNC Symbol;Acc:HGNC:5022]
ZSCAN4	27794660	27794783	5746	5869	ENSG00000180532	zinc finger and SCAN domain containing 4 [Source:HGNC Symbol;Acc:HGNC:23709]
ZNF264	27794611	27794716	5813	5918	ENSG00000083844	zinc finger protein 264 [Source:HGNC Symbol;Acc:HGNC:13057]
KDM1A	27794572	27794667	5862	5957	ENSG00000004487	lysine demethylase 1A [Source:HGNC Symbol;Acc:HGNC:29079]
ZFP64	27794529	27794622	5907	6000	ENSG00000020256	ZFP64 Zinc Finger Protein
GATA1	27794507	27794528	6001	6022	ENSG00000102145	GATA binding protein 1 [Source:HGNC Symbol;Acc:HGNC:4170]
AR	27794475	27794592	5937	6054	ENSG00000169083	androgen receptor [Source:HGNC Symbol;Acc:HGNC:644]
EZH2	27794462	27794561	5968	6067	ENSG00000106462	enhancer of zeste 2 polycomb repressive complex 2 subunit [Source:HGNC Symbol;Acc:HGNC:3527]
SMARCA4	27794460	27794565	5964	6069	ENSG00000127616	SWI/SNF related, matrix associated, actin dependent regulator of chromatin, subfamily a, member 4 [Source:HGNC Symbol;Acc:HGNC:11100]

SPI1	27794437	27794550	5979	6092	ENSG00000066336	Spi-1 proto-oncogene [Source:HGNC Symbol;Acc:HGNC:11241]
GATA4	27794421	27794468	6061	6108	ENSG00000285109	GATA binding protein 4 [Source:HGNC Symbol;Acc:HGNC:4173]
ZNF263	27794417	27794444	6085	6112	ENSG00000006194	zinc finger protein 263 [Source:HGNC Symbol;Acc:HGNC:13056]
CHD1	27794414	27794503	6026	6115	ENSG00000153922	chromodomain helicase DNA binding protein 1 [Source:HGNC Symbol;Acc:HGNC:1915]
GRHL3	27794397	27794470	6059	6132	ENSG00000158055	grainyhead like transcription factor 3 [Source:HGNC Symbol;Acc:HGNC:25839]
RUNX1	27794397	27794492	6037	6132	ENSG00000159216	RUNX family transcription factor 1 [Source:HGNC Symbol;Acc:HGNC:10471]
ZBTB48	27794391	27794478	6051	6138	ENSG00000204859	zinc finger and BTB domain containing 48 [Source:HGNC Symbol;Acc:HGNC:4930]
ASCL1	27794390	27794457	6072	6139	ENSG00000139352	achaete-scute family bHLH transcription factor 1 [Source:HGNC Symbol;Acc:HGNC:738]
GATA6	27794390	27794478	6051	6139	ENSG00000141448	GATA binding protein 6 [Source:HGNC Symbol;Acc:HGNC:4174]

AGO1	27794365	27794412	6117	6164	ENSG00000092847	argonaute RISC component 1 [Source:HGNC Symbol;Acc:HGNC:3262]
BMI1	27794365	27794450	6079	6164	ENSG00000168283	BMI1 proto-oncogene, polycomb ring finger [Source:HGNC Symbol;Acc:HGNC:1066]
ZBTB7A	27794365	27794462	6067	6164	ENSG00000178951	zinc finger and BTB domain containing 7A [Source:HGNC Symbol;Acc:HGNC:18078]
CRY1	27794358	27794459	6070	6171	ENSG00000008405	cryptochrome circadian regulator 1 [Source:HGNC Symbol;Acc:HGNC:2384]
PGR	27794358	27794467	6062	6171	ENSG00000082175	progesterone receptor [Source:HGNC Symbol;Acc:HGNC:8910]
ZEB1	27794328	27794413	6116	6201	ENSG00000148516	zinc finger E-box binding homeobox 1 [Source:HGNC Symbol;Acc:HGNC:11642]
ZBTB48	27794298	27794383	6146	6231	ENSG00000204859	zinc finger and BTB domain containing 48 [Source:HGNC Symbol;Acc:HGNC:4930]
BRD4	27794295	27794396	6133	6234	ENSG00000141867	bromodomain containing 4 [Source:HGNC Symbol;Acc:HGNC:13575]

AR	27794252	27794283	6246	6277	ENSG00000169083	androgen receptor [Source:HGNC Symbol;Acc:HGNC:644]
PPARG	27794235	27794318	6211	6294	ENSG00000132170	peroxisome proliferator activated receptor gamma [Source:HGNC Symbol;Acc:HGNC:9236]
SUZ12	27794234	27794333	6196	6295	ENSG00000178691	SUZ12 polycomb repressive complex 2 subunit [Source:HGNC Symbol;Acc:HGNC:17101]
PADI2	27794223	27794294	6235	6306	ENSG00000117115	peptidyl arginine deiminase 2 [Source:HGNC Symbol;Acc:HGNC:18341]
NR3C1	27794217	27794312	6217	6312	ENSG00000113580	nuclear receptor subfamily 3 group C member 1 [Source:HGNC Symbol;Acc:HGNC:7978]
FOXA1	27794214	27794282	6247	6315	ENSG00000129514	forkhead box A1 [Source:HGNC Symbol;Acc:HGNC:5021]
PGR	27794198	27794295	6234	6331	ENSG00000082175	progesterone receptor [Source:HGNC Symbol;Acc:HGNC:8910]
ME1	27794187	27794272	6257	6342	ENSG00000065833	malic enzyme 1 [Source:HGNC Symbol;Acc:HGNC:6983]
ESR1	27794187	27794293	6236	6342	ENSG00000091831	estrogen receptor 1 [Source:HGNC Symbol;Acc:HGNC:3467]

FOXA1	27794136	27794239	6290	6393	ENSG00000129514	forkhead box A1 [Source:HGNC Symbol;Acc:HGNC:5021]
RBM25	27794133	27794162	6367	6396	ENSG00000119707	RNA binding motif protein 25 [Source:HGNC Symbol;Acc:HGNC:23244]
NFE2	27794131	27794146	6383	6398	ENSG00000123405	nuclear factor, erythroid 2 [Source:HGNC Symbol;Acc:HGNC:7780]
MBD4	27794126	27794225	6304	6403	ENSG00000129071	methyl-CpG binding domain 4, DNA glycosylase [Source:HGNC Symbol;Acc:HGNC:6919]
STAT1	27794121	27794183	6346	6408	ENSG00000115415	signal transducer and activator of transcription 1 [Source:HGNC Symbol;Acc:HGNC:11362]
EP300	27794120	27794220	6309	6409	ENSG00000100393	E1A binding protein p300 [Source:HGNC Symbol;Acc:HGNC:3373]
EZH2	27794113	27794212	6317	6416	ENSG00000106462	enhancer of zeste 2 polycomb repressive complex 2 subunit [Source:HGNC Symbol;Acc:HGNC:3527]
NCOR1	27794103	27794192	6337	6426	ENSG00000141027	nuclear receptor corepressor 1 [Source:HGNC Symbol;Acc:HGNC:7672]

SMARCA4	27794079	27794184	6345	6450	ENSG00000127616	SWI/SNF related, matrix associated, actin dependent regulator of chromatin, subfamily a, member 4 [Source:HGNC Symbol;Acc:HGNC:11100]
OTX2	27794076	27794139	6390	6453	ENSG00000165588	orthodenticle homeobox 2 [Source:HGNC Symbol;Acc:HGNC:8522]
BRD4	27794063	27794108	6421	6466	ENSG00000141867	bromodomain containing 4 [Source:HGNC Symbol;Acc:HGNC:13575]
TBP	27794056	27794141	6388	6473	ENSG00000112592	TATA-box binding protein [Source:HGNC Symbol;Acc:HGNC:11588]
CREB5	27794020	27794065	6464	6509	ENSG00000146592	cAMP responsive element binding protein 5 [Source:HGNC Symbol;Acc:HGNC:16844]
ERF	27794015	27794043	6486	6514	ENSG00000105722	ETS2 repressor factor [Source:HGNC Symbol;Acc:HGNC:3444]
ERG	27794014	27794052	6477	6515	ENSG00000157554	ETS transcription factor ERG [Source:HGNC Symbol;Acc:HGNC:3446]
SOX8	27794012	27794101	6428	6517	ENSG00000005513	SRY-box transcription factor 8 [Source:HGNC Symbol;Acc:HGNC:11203]
E2F1	27794008	27794041	6488	6521	ENSG00000101412	E2F transcription factor 1 [Source:HGNC Symbol;Acc:HGNC:3113]

MYCN	27794008	27794103	6426	6521	ENSG00000134323	MYCN proto-oncogene, bHLH transcription factor [Source:HGNC Symbol;Acc:HGNC:7559]
GATA2	27794008	27794067	6462	6521	ENSG00000179348	GATA binding protein 2 [Source:HGNC Symbol;Acc:HGNC:4171]
NKX3-1	27794007	27794050	6479	6522	ENSG00000167034	NK3 homeobox 1 [Source:HGNC Symbol;Acc:HGNC:7838]
HOXB13	27794005	27794026	6503	6524	ENSG00000159184	homeobox B13 [Source:HGNC Symbol;Acc:HGNC:5112]
AR	27794003	27794032	6497	6526	ENSG00000169083	androgen receptor [Source:HGNC Symbol;Acc:HGNC:644]
ARID1A	27794002	27794050	6479	6527	ENSG00000117713	AT-rich interaction domain 1A [Source:HGNC Symbol;Acc:HGNC:11110]
TLE3	27793999	27794028	6501	6530	ENSG00000140332	TLE family member 3, transcriptional corepressor [Source:HGNC Symbol;Acc:HGNC:11839]
CDX2	27793998	27794038	6491	6531	ENSG00000165556	caudal type homeobox 2 [Source:HGNC Symbol;Acc:HGNC:1806]
FOXA1	27793995	27794023	6506	6534	ENSG00000129514	forkhead box A1 [Source:HGNC Symbol;Acc:HGNC:5021]
PIAS1	27793994	27794027	6502	6535	ENSG00000033800	protein inhibitor of activated STAT 1

						[Source:HGNC Symbol;Acc:HGNC:2752]
TCF7L2/LEF1	27793993	27794078	6451	6536	ENSG00000148737	transcription factor 7 like 2 [Source:HGNC Symbol;Acc:HGNC:11641]
NCOR1	27793991	27794076	6453	6538	ENSG00000141027	nuclear receptor corepressor 1 [Source:HGNC Symbol;Acc:HGNC:7672]
TRIM28	27793990	27794083	6446	6539	ENSG00000130726	tripartite motif containing 28 [Source:HGNC Symbol;Acc:HGNC:16384]
MBL2	27793990	27794030	6499	6539	ENSG00000165471	mannose binding lectin 2 [Source:HGNC Symbol;Acc:HGNC:6922]
NR3C1	27793988	27794083	6446	6541	ENSG00000113580	nuclear receptor subfamily 3 group C member 1 [Source:HGNC Symbol;Acc:HGNC:7978]
STAT1	27793984	27794061	6468	6545	ENSG00000115415	signal transducer and activator of transcription 1 [Source:HGNC Symbol;Acc:HGNC:11362]
SUMO2	27793983	27794026	6503	6546	ENSG00000188612	small ubiquitin like modifier 2 [Source:HGNC Symbol;Acc:HGNC:11125]
KDM1A	27793981	27794064	6465	6548	ENSG00000004487	lysine demethylase 1A [Source:HGNC Symbol;Acc:HGNC:29079]

NANOG	27793976	27794071	6458	6553	ENSG00000111704	Nanog homeobox [Source:HGNC Symbol;Acc:HGNC:20857]
PRDM6	27793969	27794032	6497	6560	ENSG00000061455	PR/SET domain 6 [Source:HGNC Symbol;Acc:HGNC:9350]
EP300	27793968	27794084	6445	6561	ENSG00000100393	E1A binding protein p300 [Source:HGNC Symbol;Acc:HGNC:3373]
CREB1	27793968	27794039	6490	6561	ENSG00000118260	cAMP responsive element binding protein 1 [Source:HGNC Symbol;Acc:HGNC:2345]
ASCL2	27793965	27794046	6483	6564	ENSG00000183734	achaete-scute family bHLH transcription factor 2 [Source:HGNC Symbol;Acc:HGNC:739]
MYC	27793963	27794036	6493	6566	ENSG00000136997	MYC proto-oncogene, bHLH transcription factor [Source:HGNC Symbol;Acc:HGNC:7553]
FIP1L1	27793962	27794051	6478	6567	ENSG00000145216	factor interacting with PAPOLA and CPSF1 [Source:HGNC Symbol;Acc:HGNC:19124]
ESR1	27793947	27793989	6540	6582	ENSG00000091831	estrogen receptor 1 [Source:HGNC Symbol;Acc:HGNC:3467]
MYCN	27793946	27794041	6488	6583	ENSG00000134323	MYCN proto-oncogene, bHLH transcription factor

						[Source:HGNC Symbol;Acc:HGNC:7559]
HNF4G	27793939	27793986	6543	6590	ENSG00000164749	hepatocyte nuclear factor 4 gamma [Source:HGNC Symbol;Acc:HGNC:5026]
NANOG	27793923	27794018	6511	6606	ENSG00000111704	Nanog homeobox [Source:HGNC Symbol;Acc:HGNC:20857]
EZH2	27793916	27793980	6549	6613	ENSG00000106462	enhancer of zeste 2 polycomb repressive complex 2 subunit [Source:HGNC Symbol;Acc:HGNC:3527]
ZBTB11	27793907	27793982	6547	6622	ENSG00000066422	zinc finger and BTB domain containing 11 [Source:HGNC Symbol;Acc:HGNC:16740]
BRD4	27793905	27793963	6566	6624	ENSG00000141867	bromodomain containing 4 [Source:HGNC Symbol;Acc:HGNC:13575]
ZEB2	27793893	27793962	6567	6636	ENSG00000169554	zinc finger E-box binding homeobox 2 [Source:HGNC Symbol;Acc:HGNC:14881]
EP300	27793892	27793929	6600	6637	ENSG00000100393	E1A binding protein p300 [Source:HGNC Symbol;Acc:HGNC:3373]
OGG1	27793882	27793969	6560	6647	ENSG00000114026	8-oxoguanine DNA glycosylase [Source:HGNC Symbol;Acc:HGNC:8125]

CDK9	27793877	27793978	6551	6652	ENSG00000136807	cyclin dependent kinase 9 [Source:HGNC Symbol;Acc:HGNC:1780]
CREB1	27793872	27793963	6566	6657	ENSG00000118260	cAMP responsive element binding protein 1 [Source:HGNC Symbol;Acc:HGNC:2345]
INTS11	27793872	27793959	6570	6657	ENSG00000127054	integrator complex subunit 11 [Source:HGNC Symbol;Acc:HGNC:26052]
TRPS1	27793863	27793964	6565	6666	ENSG00000104447	transcriptional repressor GATA binding 1 [Source:HGNC Symbol;Acc:HGNC:12340]
FOXA2	27793858	27793961	6568	6671	ENSG00000125798	forkhead box A2 [Source:HGNC Symbol;Acc:HGNC:5022]
ZNF423	27793857	27793958	6571	6672	ENSG00000102935	zinc finger protein 423 [Source:HGNC Symbol;Acc:HGNC:16762]
TRIM28	27793845	27793938	6591	6684	ENSG00000130726	tripartite motif containing 28 [Source:HGNC Symbol;Acc:HGNC:16384]
KDM2B	27793840	27793931	6598	6689	ENSG00000089094	lysine demethylase 2B [Source:HGNC Symbol;Acc:HGNC:13610]
BRD4	27793840	27793893	6636	6689	ENSG00000141867	bromodomain containing 4 [Source:HGNC Symbol;Acc:HGNC:13575]

WT1	27793834	27793915	6614	6695	ENSG00000184937	WT1 transcription factor [Source:HGNC Symbol;Acc:HGNC:12796]
TAF15	27793834	27793885	6644	6695	ENSG00000276833	TATA-box binding protein associated factor 15 [Source:HGNC Symbol;Acc:HGNC:11547]
SP3	27793832	27793857	6672	6697	ENSG00000172845	Sp3 transcription factor [Source:HGNC Symbol;Acc:HGNC:11208]
FOXA1	27793827	27793930	6599	6702	ENSG00000129514	forkhead box A1 [Source:HGNC Symbol;Acc:HGNC:5021]
GLIS2	27793826	27793851	6678	6703	ENSG00000274636	GLIS family zinc finger 2 [Source:HGNC Symbol;Acc:HGNC:29450]
VEZF1	27793818	27793865	6664	6711	ENSG00000136451	vascular endothelial zinc finger 1 [Source:HGNC Symbol;Acc:HGNC:12949]
L3MBTL2	27793815	27793894	6635	6714	ENSG00000100395	L3MBTL histone methyl-lysine binding protein 2 [Source:HGNC Symbol;Acc:HGNC:18594]
EGR2	27793815	27793880	6649	6714	ENSG00000122877	early growth response 2 [Source:HGNC Symbol;Acc:HGNC:3239]
ZNF76	27793812	27793885	6644	6717	ENSG00000065029	zinc finger protein 76 [Source:HGNC Symbol;Acc:HGNC:13149]
NFYA	27793807	27793886	6643	6722	ENSG00000001167	nuclear transcription factor Y subunit alpha

						[Source:HGNC Symbol;Acc:HGNC:7804]
ZFP64	27793801	27793870	6659	6728	ENSG00000020256	ZFP64 Zinc Finger Protein
EGR1	27793801	27793896	6633	6728	ENSG00000120738	early growth response 1 [Source:HGNC Symbol;Acc:HGNC:3238]
ZBTB48	27793798	27793843	6686	6731	ENSG00000204859	zinc finger and BTB domain containing 48 [Source:HGNC Symbol;Acc:HGNC:4930]
SUZ12	27793794	27793869	6660	6735	ENSG00000178691	SUZ12 polycomb repressive complex 2 subunit [Source:HGNC Symbol;Acc:HGNC:17101]
PGR	27793792	27793889	6640	6737	ENSG00000082175	progesterone receptor [Source:HGNC Symbol;Acc:HGNC:8910]
TRIM24	27793792	27793888	6641	6737	ENSG00000122779	tripartite motif containing 24 [Source:HGNC Symbol;Acc:HGNC:11812]
NR2F2	27793790	27793869	6660	6739	ENSG00000185551	nuclear receptor subfamily 2 group F member 2 [Source:HGNC Symbol;Acc:HGNC:7976]
ARNTL	27793787	27793862	6667	6742	ENSG00000133794	aryl hydrocarbon receptor nuclear translocator like [Source:HGNC Symbol;Acc:HGNC:701]
POU5F1	27793783	27793874	6655	6746	ENSG00000206454	POU class 5 homeobox 1 [Source:HGNC Symbol;Acc:HGNC:9221]

EP300	27793780	27793879	6650	6749	ENSG00000100393	E1A binding protein p300 [Source:HGNC Symbol;Acc:HGNC:3373]
XRCC5	27793776	27793797	6732	6753	ENSG00000079246	X-ray repair cross complementing 5 [Source:HGNC Symbol;Acc:HGNC:12833]
SS18	27793775	27793868	6661	6754	ENSG00000141380	SS18 subunit of BAF chromatin remodeling complex [Source:HGNC Symbol;Acc:HGNC:11340]
FEZF1	27793774	27793837	6692	6755	ENSG00000128610	FEZ family zinc finger 1 [Source:HGNC Symbol;Acc:HGNC:22788]
RBBP5	27793769	27793862	6667	6760	ENSG00000117222	RB binding protein 5, histone lysine methyltransferase complex subunit [Source:HGNC Symbol;Acc:HGNC:9888]
ELF1	27793767	27793844	6685	6762	ENSG00000120690	E74 like ETS transcription factor 1 [Source:HGNC Symbol;Acc:HGNC:3316]
FGFR1	27793763	27793838	6691	6766	ENSG00000077782	fibroblast growth factor receptor 1 [Source:HGNC Symbol;Acc:HGNC:3688]
KDM2B	27793762	27793847	6682	6767	ENSG00000089094	lysine demethylase 2B [Source:HGNC Symbol;Acc:HGNC:13610]

GLIS1	27793762	27793855	6674	6767	ENSG00000174332	GLIS family zinc finger 1 [Source:HGNC Symbol;Acc:HGNC:29525]
RARA	27793761	27793856	6673	6768	ENSG00000131759	retinoic acid receptor alpha [Source:HGNC Symbol;Acc:HGNC:9864]
EGR2	27793758	27793823	6706	6771	ENSG00000122877	early growth response 2 [Source:HGNC Symbol;Acc:HGNC:3239]
AGO1	27793755	27793776	6753	6774	ENSG00000092847	argonaute RISC component 1 [Source:HGNC Symbol;Acc:HGNC:3262]
PDX1	27793755	27793826	6703	6774	ENSG00000139515	pancreatic and duodenal homeobox 1 [Source:HGNC Symbol;Acc:HGNC:6107]
ERG	27793754	27793834	6695	6775	ENSG00000157554	ETS transcription factor ERG [Source:HGNC Symbol;Acc:HGNC:3446]
MAZ	27793750	27793837	6692	6779	ENSG00000103495	MYC associated zinc finger protein [Source:HGNC Symbol;Acc:HGNC:6914]
KLF9	27793749	27793820	6709	6780	ENSG00000119138	Kruppel like factor 9 [Source:HGNC Symbol;Acc:HGNC:1123]
ZFHX2	27793749	27793882	6647	6780	ENSG00000136367	zinc finger homeobox 2 [Source:HGNC Symbol;Acc:HGNC:20152]

HNRNPLL	27793749	27793814	6715	6780	ENSG00000143889	heterogeneous nuclear ribonucleoprotein L like [Source:HGNC Symbol;Acc:HGNC:25127]
TAL1	27793749	27793816	6713	6780	ENSG00000162367	TAL bHLH transcription factor 1, erythroid differentiation factor [Source:HGNC Symbol;Acc:HGNC:11556]
TCF7L1/LEF1	27793746	27793823	6706	6783	ENSG00000152284	transcription factor 7 like 1 [Source:HGNC Symbol;Acc:HGNC:11640]
FLI1	27793734	27793825	6704	6795	ENSG00000151702	Fli-1 proto-oncogene, ETS transcription factor [Source:HGNC Symbol;Acc:HGNC:3749]
SP4	27793732	27793811	6718	6797	ENSG00000105866	Sp4 transcription factor [Source:HGNC Symbol;Acc:HGNC:11209]
SP2	27793732	27793809	6720	6797	ENSG00000167182	Sp2 transcription factor [Source:HGNC Symbol;Acc:HGNC:11207]
SP1	27793732	27793819	6710	6797	ENSG00000185591	Sp1 transcription factor [Source:HGNC Symbol;Acc:HGNC:11205]
TFAP4	27793731	27793796	6733	6798	ENSG00000090447	transcription factor AP-4 [Source:HGNC Symbol;Acc:HGNC:11745]
PGR	27793730	27793821	6708	6799	ENSG00000082175	progesterone receptor [Source:HGNC Symbol;Acc:HGNC:8910]

HIF1A	27793729	27793812	6717	6800	ENSG00000100644	hypoxia inducible factor 1 subunit alpha [Source:HGNC Symbol;Acc:HGNC:4910]
CCNT2	27793728	27793881	6648	6801	ENSG00000082258	cyclin T2 [Source:HGNC Symbol;Acc:HGNC:1600]
SMC1A	27793727	27793820	6709	6802	ENSG00000072501	structural maintenance of chromosomes 1A [Source:HGNC Symbol;Acc:HGNC:11111]
ZNF554	27793726	27793751	6778	6803	ENSG00000172006	zinc finger protein 554 [Source:HGNC Symbol;Acc:HGNC:26629]
HEXIM1	27793726	27793747	6782	6803	ENSG00000186834	HEXIM P-TEFb complex subunit 1 [Source:HGNC Symbol;Acc:HGNC:24953]
RUNX1	27793725	27793785	6744	6804	ENSG00000159216	RUNX family transcription factor 1 [Source:HGNC Symbol;Acc:HGNC:10471]
ZBTB14	27793725	27793738	6791	6804	ENSG00000198081	zinc finger and BTB domain containing 14 [Source:HGNC Symbol;Acc:HGNC:12860]
OTX2	27793722	27793823	6706	6807	ENSG00000165588	orthodenticle homeobox 2 [Source:HGNC Symbol;Acc:HGNC:8522]
AR	27793722	27793784	6745	6807	ENSG00000169083	androgen receptor [Source:HGNC Symbol;Acc:HGNC:644]

EGR3	27793722	27793849	6680	6807	ENSG00000179388	early growth response 3 [Source:HGNC Symbol;Acc:HGNC:3240]
E2F1	27793721	27793760	6769	6808	ENSG00000101412	E2F transcription factor 1 [Source:HGNC Symbol;Acc:HGNC:3113]
SMARCA4	27793721	27793830	6699	6808	ENSG00000127616	SWI/SNF related, matrix associated, actin dependent regulator of chromatin, subfamily a, member 4 [Source:HGNC Symbol;Acc:HGNC:11100]
UPF1	27793719	27793820	6709	6810	ENSG00000005007	UPF1 RNA helicase and ATPase [Source:HGNC Symbol;Acc:HGNC:9962]
PCBP1	27793719	27793786	6743	6810	ENSG00000169564	poly(rC) binding protein 1 [Source:HGNC Symbol;Acc:HGNC:8647]
ZBTB26	27793719	27793806	6723	6810	ENSG00000171448	zinc finger and BTB domain containing 26 [Source:HGNC Symbol;Acc:HGNC:23383]
FOXA1	27793718	27793801	6728	6811	ENSG00000129514	forkhead box A1 [Source:HGNC Symbol;Acc:HGNC:5021]
CDX2	27793718	27793803	6726	6811	ENSG00000165556	caudal type homeobox 2 [Source:HGNC Symbol;Acc:HGNC:1806]
MYCN	27793714	27793749	6780	6815	ENSG00000134323	MYCN proto-oncogene, bHLH transcription factor

						[Source:HGNC Symbol;Acc:HGNC:7559]
CTCF	27793712	27793759	6770	6817	ENSG00000102974	CCCTC-binding factor [Source:HGNC Symbol;Acc:HGNC:13723]
USP7	27793708	27793783	6746	6821	ENSG00000187555	ubiquitin specific peptidase 7 [Source:HGNC Symbol;Acc:HGNC:12630]
CNOT3	27793706	27793787	6742	6823	ENSG00000276082	CCR4-NOT transcription complex subunit 3 [Source:HGNC Symbol;Acc:HGNC:7879]
GATA6	27793705	27793802	6727	6824	ENSG00000141448	GATA binding protein 6 [Source:HGNC Symbol;Acc:HGNC:4174]
MYC	27793701	27793746	6783	6828	ENSG00000136997	MYC proto-oncogene, bHLH transcription factor [Source:HGNC Symbol;Acc:HGNC:7553]
CHD7	27793701	27793792	6737	6828	ENSG00000171316	chromodomain helicase DNA binding protein 7 [Source:HGNC Symbol;Acc:HGNC:20626]
EP300	27793699	27793800	6729	6830	ENSG00000100393	E1A binding protein p300 [Source:HGNC Symbol;Acc:HGNC:3373]
SMAD3	27793699	27793782	6747	6830	ENSG00000166949	SMAD family member 3 [Source:HGNC Symbol;Acc:HGNC:6769]

RUNX1	27793698	27793799	6730	6831	ENSG00000159216	RUNX Family Transcription Factor 1
TEAD4	27793695	27793806	6723	6834	ENSG00000197905	TEA domain transcription factor 4 [Source:HGNC Symbol;Acc:HGNC:11717]
SMARCC1	27793692	27793749	6780	6837	ENSG00000173473	SWI/SNF related, matrix associated, actin dependent regulator of chromatin subfamily c member 1 [Source:HGNC Symbol;Acc:HGNC:11104]
TFAP2C	27793691	27793789	6740	6838	ENSG00000087510	transcription factor AP-2 gamma [Source:HGNC Symbol;Acc:HGNC:11744]
GATA1	27793691	27793769	6760	6838	ENSG00000102145	GATA binding protein 1 [Source:HGNC Symbol;Acc:HGNC:4170]
GABPB1	27793690	27793769	6760	6839	ENSG00000104064	GA binding protein transcription factor subunit beta 1 [Source:HGNC Symbol;Acc:HGNC:4074]
FOXA2	27793690	27793786	6743	6839	ENSG00000125798	forkhead box A2 [Source:HGNC Symbol;Acc:HGNC:5022]
PTEN	27793690	27793775	6754	6839	ENSG00000171862	phosphatase and tensin homolog [Source:HGNC Symbol;Acc:HGNC:9588]
RAD21	27793688	27793760	6769	6841	ENSG00000164754	RAD21 cohesin complex component [Source:HGNC Symbol;Acc:HGNC:9811]

YY1	27793686	27793795	6734	6843	ENSG00000100811	YY1 transcription factor [Source:HGNC Symbol;Acc:HGNC:12856]
ZNF35	27793685	27793786	6743	6844	ENSG00000281306	zinc finger protein 35 [Source:HGNC Symbol;Acc:HGNC:13099]
PAX5	27793683	27793780	6749	6846	ENSG00000196092	paired box 5 [Source:HGNC Symbol;Acc:HGNC:8619]
JUND	27793682	27793743	6786	6847	ENSG00000130522	JunD proto-oncogene, AP-1 transcription factor subunit [Source:HGNC Symbol;Acc:HGNC:6206]
PDX1	27793682	27793745	6784	6847	ENSG00000139515	pancreatic and duodenal homeobox 1 [Source:HGNC Symbol;Acc:HGNC:6107]
HSF1	27793682	27793771	6758	6847	ENSG00000284774	heat shock transcription factor 1 [Source:HGNC Symbol;Acc:HGNC:5224]
NCAPH2	27793681	27793766	6763	6848	ENSG00000025770	non-SMC condensin II complex subunit H2 [Source:HGNC Symbol;Acc:HGNC:25071]
JMJD6	27793681	27793768	6761	6848	ENSG00000070495	jumonji domain containing 6, arginine demethylase and lysine hydroxylase [Source:HGNC Symbol;Acc:HGNC:19355]

NRF1	27793681	27793781	6748	6848	ENSG00000106459	nuclear respiratory factor 1 [Source:HGNC Symbol;Acc:HGNC:7996]
SRF	27793681	27793760	6769	6848	ENSG00000112658	serum response factor [Source:HGNC Symbol;Acc:HGNC:11291]
PRDM9	27793681	27793752	6777	6848	ENSG00000164256	PR/SET domain 9 [Source:HGNC Symbol;Acc:HGNC:13994]
CASZ1	27793679	27793772	6757	6850	ENSG00000130940	castor zinc finger 1 [Source:HGNC Symbol;Acc:HGNC:26002]
GATA2	27793679	27793756	6773	6850	ENSG00000179348	GATA binding protein 2 [Source:HGNC Symbol;Acc:HGNC:4171]
NCOR1	27793674	27793759	6770	6855	ENSG00000141027	nuclear receptor corepressor 1 [Source:HGNC Symbol;Acc:HGNC:7672]
PIAS1	27793669	27793744	6785	6860	ENSG00000033800	protein inhibitor of activated STAT 1 [Source:HGNC Symbol;Acc:HGNC:2752]
ARNTL	27793668	27793765	6764	6861	ENSG00000133794	aryl hydrocarbon receptor nuclear translocator like [Source:HGNC Symbol;Acc:HGNC:701]
ESR1	27793666	27793765	6764	6863	ENSG00000091831	estrogen receptor 1 [Source:HGNC Symbol;Acc:HGNC:3467]

RXRA	27793665	27793754	6775	6864	ENSG00000186350	retinoid X receptor alpha [Source:HGNC Symbol;Acc:HGNC:10477]
BRD4	27793664	27793773	6756	6865	ENSG00000141867	bromodomain containing 4 [Source:HGNC Symbol;Acc:HGNC:13575]
AFF1	27793664	27793739	6790	6865	ENSG00000172493	AF4/FMR2 family member 1 [Source:HGNC Symbol;Acc:HGNC:7135]
CXXC4	27793663	27793764	6765	6866	ENSG00000168772	CXXC Finger Protein 4
TRPS1	27793662	27793753	6776	6867	ENSG00000104447	transcriptional repressor GATA binding 1 [Source:HGNC Symbol;Acc:HGNC:12340]
GABPA	27793660	27793759	6770	6869	ENSG00000154727	GA binding protein transcription factor subunit alpha [Source:HGNC Symbol;Acc:HGNC:4071]
ZBTB7A	27793660	27793747	6782	6869	ENSG00000178951	zinc finger and BTB domain containing 7A [Source:HGNC Symbol;Acc:HGNC:18078]
EZH2	27793659	27793758	6771	6870	ENSG00000106462	enhancer of zeste 2 polycomb repressive complex 2 subunit [Source:HGNC Symbol;Acc:HGNC:3527]
POU5F1	27793657	27793748	6781	6872	ENSG00000206454	POU class 5 homeobox 1 [Source:HGNC Symbol;Acc:HGNC:9221]

SP1	27793656	27793763	6766	6873	ENSG00000185591	Sp1 transcription factor [Source:HGNC Symbol;Acc:HGNC:11205]
HDAC2	27793656	27793761	6768	6873	ENSG00000196591	histone deacetylase 2 [Source:HGNC Symbol;Acc:HGNC:4853]
ELF1	27793654	27793743	6786	6875	ENSG00000120690	E74 like ETS transcription factor 1 [Source:HGNC Symbol;Acc:HGNC:3316]
SIRT6	27793651	27793744	6785	6878	ENSG00000077463	sirtuin 6 [Source:HGNC Symbol;Acc:HGNC:14934]
LARP7	27793651	27793752	6777	6878	ENSG00000174720	La ribonucleoprotein 7, transcriptional regulator [Source:HGNC Symbol;Acc:HGNC:24912]
KLF5	27793647	27793740	6789	6882	ENSG00000102554	Kruppel like factor 5 [Source:HGNC Symbol;Acc:HGNC:6349]
AFF4	27793646	27793739	6790	6883	ENSG00000072364	AF4/FMR2 family member 4 [Source:HGNC Symbol;Acc:HGNC:17869]
SUZ12	27793643	27793748	6781	6886	ENSG00000178691	SUZ12 polycomb repressive complex 2 subunit [Source:HGNC Symbol;Acc:HGNC:17101]

Table S1: Transcription factor identified for binding to the putative human NOS2-2 promoter

Listed the names of the transcription factors (TF), which have been identified by ChIP-assays (analysis using BioUML [1]) to interact with the putative NOS2-2 promoter sequence

(between exon 2 and exon 1-diff in the human NOS2 gene). Shown are the transcription factor title, the start and end of the ChIP Fragment on chromosome 17 (**Ch17**) and relative to the start of the NOS2 gene (**G**), the ENSEMBL gene ID and a short description of the TF.

**Name Transcript**	**Gene stable ID**	**Gene description**	**comment**
AASS_2	ENSG00000008311	aminoadipate-semialdehyde synthase [Source:HGNC Symbol;Acc:HGNC:17366]	
ACAP1_1	ENSG00000072818	ArfGAP with coiled-coil, ankyrin repeat and PH domains 1 [Source:HGNC Symbol;Acc:HGNC:16467]	
AKAP1_2	ENSG00000121057	A-kinase anchoring protein 1 [Source:HGNC Symbol;Acc:HGNC:367]	RNABP
ARL4C_1	ENSG00000188042	ADP ribosylation factor like GTPase 4C [Source:HGNC Symbol;Acc:HGNC:698]	
B3GNT7_1	ENSG00000156966	UDP-GlcNAc:betaGal beta-1,3-N-acetylglucosaminyltransferase 7 [Source:HGNC Symbol;Acc:HGNC:18811]	
BMI1_1	ENSG00000168283	BMI1 proto-oncogene, polycomb ring finger [Source:HGNC Symbol;Acc:HGNC:1066]	TF
BMP5_1	ENSG00000112175	bone morphogenetic protein 5 [Source:HGNC Symbol;Acc:HGNC:1072]	

CARNMT1_1	ENSG00000156017	carnosine N-methyltransferase 1 [Source:HGNC Symbol;Acc:HGNC:23435]	
CCDC92_5	ENSG00000119242	coiled-coil domain containing 92 [Source:HGNC Symbol;Acc:HGNC:29563]	
CEBPZ_1	ENSG00000115816	CCAAT enhancer binding protein zeta [Source:HGNC Symbol;Acc:HGNC:24218]	RNABP
CHCHD3_1	ENSG00000106554	coiled-coil-helix-coiled-coil-helix domain containing 3 [Source:HGNC Symbol;Acc:HGNC:21906]	
CHST4_2	ENSG00000140835	carbohydrate sulfotransferase 4 [Source:HGNC Symbol;Acc:HGNC:1972]	
CLDN10_2	ENSG00000134873	claudin 10 [Source:HGNC Symbol;Acc:HGNC:2033]	
CNMD_2	ENSG00000136110	chondromodulin [Source:HGNC Symbol;Acc:HGNC:17005]	
CYP2S1_2	ENSG00000167600	cytochrome P450 family 2 subfamily S member 1 [Source:HGNC Symbol;Acc:HGNC:15654]	
DNMT3B_1	ENSG00000088305	DNA methyltransferase 3 beta [Source:HGNC Symbol;Acc:HGNC:2979]	RNABP
DSCC1_1	ENSG00000136982	DNA replication and sister chromatid cohesion 1	

		[Source:HGNC Symbol;Acc:HGNC:24453]	
EFNB1_1	ENSG00000090776	ephrin B1 [Source:HGNC Symbol;Acc:HGNC:3226]	
EIF2AK4_1	ENSG00000128829	eukaryotic translation initiation factor 2 alpha kinase 4 [Source:HGNC Symbol;Acc:HGNC:19687]	RNABP
EIF5A_8	ENSG00000132507	eukaryotic translation initiation factor 5A [Source:HGNC Symbol;Acc:HGNC:3300]	RNABP
ENOPH1_1	ENSG00000145293	enolase-phosphatase 1 [Source:HGNC Symbol;Acc:HGNC:24599]	
FAM160A1_1	ENSG00000164142	family with sequence similarity 160 member A1 [Source:HGNC Symbol;Acc:HGNC:34237]	
FBN2_1	ENSG00000138829	fibrillin 2 [Source:HGNC Symbol;Acc:HGNC:3604]	
FKBP4_1	ENSG00000004478	FKBP prolyl isomerase 4 [Source:HGNC Symbol;Acc:HGNC:3720]	
GABRB3_7	ENSG00000166206	gamma-aminobutyric acid type A receptor subunit beta3 [Source:HGNC Symbol;Acc:HGNC:4083]	
GADD45GIP1_1	ENSG00000179271	GADD45G interacting protein 1 [Source:HGNC Symbol;Acc:HGNC:29996]	

GLB1L3_1	ENSG00000166105	galactosidase beta 1 like 3 [Source:HGNC Symbol;Acc:HGNC:25147]	
GNL2_2	ENSG00000134697	G protein nucleolar 2 [Source:HGNC Symbol;Acc:HGNC:29925]	RNABP
GPR176_1	ENSG00000166073	G protein-coupled receptor 176 [Source:HGNC Symbol;Acc:HGNC:32370]	
HGSNAT_1	ENSG00000165102	heparan-alpha-glucosaminide N-acetyltransferase [Source:HGNC Symbol;Acc:HGNC:26527]	
HSPE1_4	ENSG00000115541	heat shock protein family E (Hsp10) member 1 [Source:HGNC Symbol;Acc:HGNC:5269]	
IER5L_1	ENSG00000188483	immediate early response 5 like [Source:HGNC Symbol;Acc:HGNC:23679]	
INTS13_2	ENSG00000064102	integrator complex subunit 13 [Source:HGNC Symbol;Acc:HGNC:20174]	
JARID2_1	ENSG00000008083	jumonji and AT-rich interaction domain containing 2 [Source:HGNC Symbol;Acc:HGNC:6196]	TF
KIF1A_1	ENSG00000130294	kinesin family member 1A [Source:HGNC Symbol;Acc:HGNC:888]	

L1TD1_1	ENSG00000240563	LINE1 type transposase domain containing 1 [Source:HGNC Symbol;Acc:HGNC:25595]	RNABP
LEF1_1	ENSG00000138795	lymphoid enhancer binding factor 1 [Source:HGNC Symbol;Acc:HGNC:6551]	TF (Syn: TCF1/7/10)
LEFTY1_1	ENSG00000243709	left-right determination factor 1 [Source:HGNC Symbol;Acc:HGNC:6552]	
LIX1_1	ENSG00000145721	limb and CNS expressed 1 [Source:HGNC Symbol;Acc:HGNC:18581]	
LRP2_1	ENSG00000081479	LDL receptor related protein 2 [Source:HGNC Symbol;Acc:HGNC:6694]	
LRP2_6	ENSG00000081479	LDL receptor related protein 2 [Source:HGNC Symbol;Acc:HGNC:6694]	
MAL2_3	ENSG00000147676	mal, T cell differentiation protein 2 [Source:HGNC Symbol;Acc:HGNC:13634]	
MAP3K21_1	ENSG00000143674	mitogen-activated protein kinase kinase kinase 21 [Source:HGNC Symbol;Acc:HGNC:29798]	
MEIS1_2	ENSG00000143995	Meis homeobox 1 [Source:HGNC Symbol;Acc:HGNC:7000]	TF

MSH2_7	ENSG00000095002	mutS homolog 2 [Source:HGNC Symbol;Acc:HGNC:7325]	
NANOG_2	ENSG00000111704	Nanog homeobox [Source:HGNC Symbol;Acc:HGNC:20857]	TF
NBPF1_1	ENSG00000219481	NBPF member 1 [Source:HGNC Symbol;Acc:HGNC:26088]	
NCOA6_6	ENSG00000198646	nuclear receptor coactivator 6 [Source:HGNC Symbol;Acc:HGNC:15936]	
NDUFAB1_2	ENSG00000004779	NADH:ubiquinone oxidoreductase subunit AB1 [Source:HGNC Symbol;Acc:HGNC:7694]	
NLGN4X_3	ENSG00000146938	neuroligin 4 X-linked [Source:HGNC Symbol;Acc:HGNC:14287]	
NLN_1	ENSG00000123213	neurolysin [Source:HGNC Symbol;Acc:HGNC:16058]	
NOCT_1	ENSG00000151014	nocturnin [Source:HGNC Symbol;Acc:HGNC:14254]	RNABP
NOS2_2	ENSG00000007171	nitric oxide synthase 2 [Source:HGNC Symbol;Acc:HGNC:7873]	
P2RY1_1	ENSG00000169860	purinergic receptor P2Y1 [Source:HGNC Symbol;Acc:HGNC:8539]	
PCNX2_1	ENSG00000135749	pecanex 2 [Source:HGNC Symbol;Acc:HGNC:8736]	

PDZD4_3	ENSG00000067840	PDZ domain containing 4 [Source:HGNC Symbol;Acc:HGNC:21167]	
PHC2_3	ENSG00000134686	polyhomeotic homolog 2 [Source:HGNC Symbol;Acc:HGNC:3183]	
PIM2_1	ENSG00000102096	Pim-2 proto-oncogene, serine/threonine kinase [Source:HGNC Symbol;Acc:HGNC:8987]	
PIPOX_6	ENSG00000179761	pipecolic acid and sarcosine oxidase [Source:HGNC Symbol;Acc:HGNC:17804]	
PLAGL1_4	ENSG00000118495	PLAG1 like zinc finger 1 [Source:HGNC Symbol;Acc:HGNC:9046]	TF
PLPP2_3	ENSG00000141934	phospholipid phosphatase 2 [Source:HGNC Symbol;Acc:HGNC:9230]	
PMAIP1_1	ENSG00000141682	phorbol-12-myristate-13-acetate-induced protein 1 [Source:HGNC Symbol;Acc:HGNC:9108]	
PODXL_2	ENSG00000128567	podocalyxin like [Source:HGNC Symbol;Acc:HGNC:9171]	
POLR1F_1	ENSG00000105849	RNA polymerase I subunit F [Source:HGNC Symbol;Acc:HGNC:18027]	

POU5F1_1	ENSG00000204531	POU class 5 homeobox 1 [Source:HGNC Symbol;Acc:HGNC:9221]	TF (Syn:OCT3/4)
PPAT_1	ENSG00000128059	phosphoribosyl pyrophosphate amidotransferase [Source:HGNC Symbol;Acc:HGNC:9238]	
PPP1R16B_1	ENSG00000101445	protein phosphatase 1 regulatory subunit 16B [Source:HGNC Symbol;Acc:HGNC:15850]	
PRDM14_1	ENSG00000147596	PR/SET domain 14 [Source:HGNC Symbol;Acc:HGNC:14001]	TF
PRIM2_5	ENSG00000146143	DNA primase subunit 2 [Source:HGNC Symbol;Acc:HGNC:9370]	
PRKCI_1	ENSG00000163558	protein kinase C iota [Source:HGNC Symbol;Acc:HGNC:9404]	
PRTG_1	ENSG00000166450	protogenin [Source:HGNC Symbol;Acc:HGNC:26373]	
PTMA_5	ENSG00000187514	prothymosin alpha [Source:HGNC Symbol;Acc:HGNC:9623]	
RAI1_1	ENSG00000108557	retinoic acid induced 1 [Source:HGNC Symbol;Acc:HGNC:9834]	
RCC2_1	ENSG00000179051	regulator of chromosome condensation 2	

		[Source:HGNC Symbol;Acc:HGNC:30297]	
RGL1_2	ENSG00000143344	ral guanine nucleotide dissociation stimulator like 1 [Source:HGNC Symbol;Acc:HGNC:30281]	
RHOU_1	ENSG00000116574	ras homolog family member U [Source:HGNC Symbol;Acc:HGNC:17794]	
RNF168_1	ENSG00000163961	ring finger protein 168 [Source:HGNC Symbol;Acc:HGNC:26661]	
RRAS2_1	ENSG00000133818	RAS related 2 [Source:HGNC Symbol;Acc:HGNC:17271]	
SCUBE3_1	ENSG00000146197	signal peptide, CUB domain and EGF like domain containing 3 [Source:HGNC Symbol;Acc:HGNC:13655]	
SET_2	ENSG00000119335	SET nuclear proto-oncogene [Source:HGNC Symbol;Acc:HGNC:10760]	
SLC29A1_9	ENSG00000112759	solute carrier family 29 member 1 (Augustine blood group) [Source:HGNC Symbol;Acc:HGNC:11003]	
SNRPN_10	ENSG00000128739	small nuclear ribonucleoprotein polypeptide N [Source:HGNC Symbol;Acc:HGNC:11164]	RNABP

THY1_1	ENSG00000154096	Thy-1 cell surface antigen [Source:HGNC Symbol;Acc:HGNC:11801]	
TRNP1_1	ENSG00000253368	TMF1 regulated nuclear protein 1 [Source:HGNC Symbol;Acc:HGNC:34348]	
TSC22D1_1	ENSG00000102804	TSC22 domain family member 1 [Source:HGNC Symbol;Acc:HGNC:16826]	TF
TTC9_1	ENSG00000133985	tetratricopeptide repeat domain 9 [Source:HGNC Symbol;Acc:HGNC:20267]	
TTLL12_1	ENSG00000100304	tubulin tyrosine ligase like 12 [Source:HGNC Symbol;Acc:HGNC:28974]	
TUBA4A_1	ENSG00000127824	tubulin alpha 4a [Source:HGNC Symbol;Acc:HGNC:12407]	
UCK2_1	ENSG00000143179	uridine-cytidine kinase 2 [Source:HGNC Symbol;Acc:HGNC:12562]	
UGP2_25	ENSG00000169764	UDP-glucose pyrophosphorylase 2 [Source:HGNC Symbol;Acc:HGNC:12527]	
USO1_1	ENSG00000138768	USO1 vesicle transport factor [Source:HGNC Symbol;Acc:HGNC:30904]	
USP44_1	ENSG00000136014	ubiquitin specific peptidase 44 [Source:HGNC Symbol;Acc:HGNC:20064]	

USP9X_1	ENSG00000124486	ubiquitin specific peptidase 9 X-linked [Source:HGNC Symbol;Acc:HGNC:12632]	
VASH2_2	ENSG00000143494	vasohibin 2 [Source:HGNC Symbol;Acc:HGNC:25723]	
VSIG10_1	ENSG00000176834	V-set and immunoglobulin domain containing 10 [Source:HGNC Symbol;Acc:HGNC:26078]	
WDR12_1	ENSG00000138442	WD repeat domain 12 [Source:HGNC Symbol;Acc:HGNC:14098]	RNABP
WLS_1	ENSG00000116729	Wnt ligand secretion mediator [Source:HGNC Symbol;Acc:HGNC:30238]	
ZDHHC22_1	ENSG00000177108	zinc finger DHHC-type palmitoyltransferase 22 [Source:HGNC Symbol;Acc:HGNC:20106]	
ZFP42_1	ENSG00000179059	ZFP42 zinc finger protein [Source:HGNC Symbol;Acc:HGNC:30949]	TF
ZNF436_2	ENSG00000125945	zinc finger protein 436 [Source:HGNC Symbol;Acc:HGNC:20814]	TF
ZNF503_1	ENSG00000165655	zinc finger protein 503 [Source:HGNC Symbol;Acc:HGNC:23589]	TF
ZNF703_1	ENSG00000183779	zinc finger protein 703 [Source:HGNC Symbol;Acc:HGNC:25883]	TF

ZNF770_1	ENSG00000198146	zinc finger protein 770 [Source:HGNC Symbol;Acc:HGNC:26061]	TF

Table S2: Transcripts up- or downregulated in all SRA-Data analyses

Listed the names of the transcripts which are up- or down-regulated in the same manner as the NOS2-2 transcript in all SRA data analyzed (PRJNA565303, PRJNA565303, CNP0000771, PRJDB1099_Down, PRJDB1099_WT, PRJNA244622, PRJNA338181_C15, PRJNA338181_H9, PRJNA674506, PRJNA59633). Shown are the names of the transcripts, the ENSEMBL-ID of the genes, a short description, and a comment indicating if the gene is known to be a transcription factor (**TF**; [2]) or RNA binding protein (**RNABP**; [3]).

Supplemental Figures

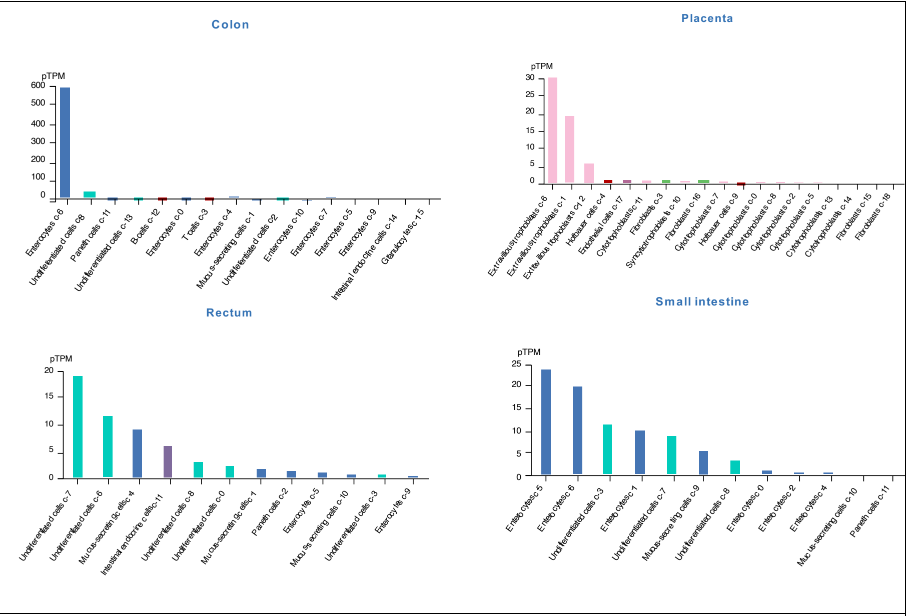

Figure S1: NOS2 mRNA isoform expression in different human tissues and cell types (copied from https://www.proteinatlas.org/ENSG00000007171-NOS2/celltype)

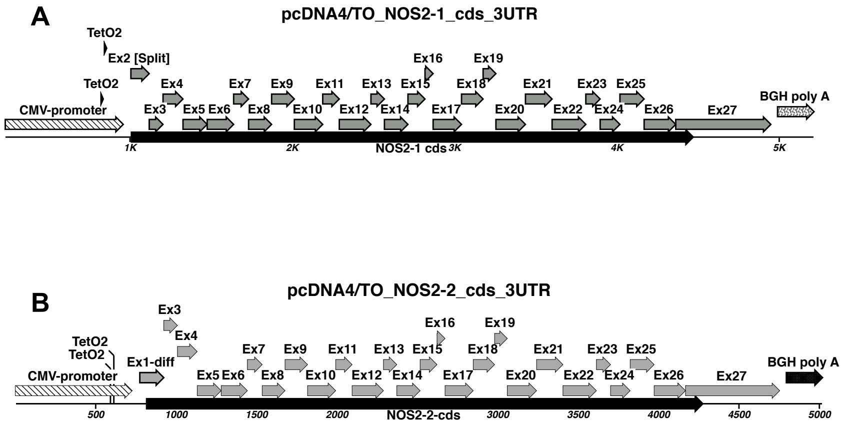

Figure S2: Scheme of the pcDNA4/TO constructs containing the NOS2-1_cds_3UTR or the NOS2-2_cds_3UTR used in transient transfection analyses.

A: pcDNA4/TO_NOS2-1_cds_3UTR: The exon sequences used, and the coding sequence (**cds**) is shown. Also, the cytomegalo-virus (**CMV**)-promoter and the bovine growth hormone (**BGH**) polyA signal important for expression are indicated. In addition, the binding sites of the tetracycline repressor (**TetO**) are shown. This TetO sites are important for the inhibition of the CMV-promoter in cells expressing the tetracycline repressor.

B: pcDNA4/TO_NOS2-2_cds_3UTR: Same as A, but the replacement of exon 2 by exon1-diff is indicated.

UCSC Genome Browser on Human Dec. 2013 (GRCh38/hg38) Assembly

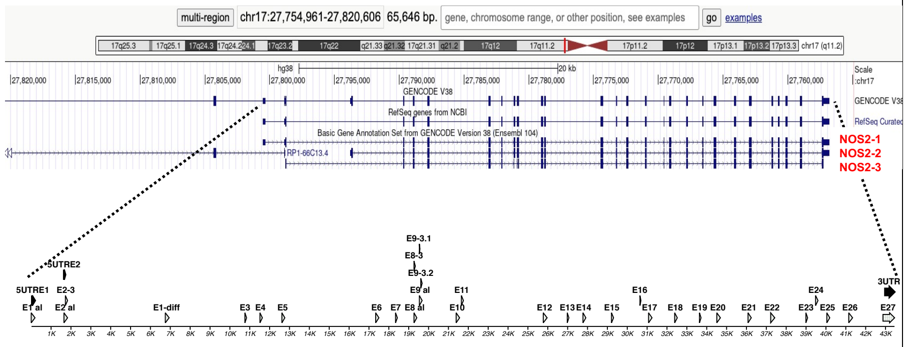

Figure S3: Scheme of the human NOS2 gene as presented in the UCSC genome browser. In the upper part a copy of the scheme of the human NOS2 gene as presented by the UCSC genome browser (on human December 2013 – GRCh38/hg38 – assembly) is shown (GENCODE V38 and RefSeq genes from NCBI). In the 5'-upstream sequence of the NOS2 gene a part of the RPI-66C13.4 gene is shown. Also, the three NOS2 mRNA isoforms (NOS2-1, -2, -3) encoded by the NOS2 gene are presented. The human NOS 2 gene is located on the human chromosome 17 (27756766-27800529 on the opposite strand).

In the lower part a scheme of the human NOS2 gene with important sequence features is presented. Shown are the exons (E1 to E27). Exon differently used in the three transcripts are signed by **al**. The additional exon **E1-diff** used in the NOS2-2 mRNA is indicated. The 5'-UTR (**5UTR**) of the NOS2-1 mRNA is encoded be E1 and partly by E2. The 3'-UTR (3UTR) is encoded by Ex27. In the NOS2-3 mRNA a shortened Ex27 (**Ex27-3)** sequence is found. In the NOS2-3 mRNA exons 8 and 9 are replaced by the exons E8-2, E9-3.1 and E9-3.2.

hat gelöscht: seuqnce

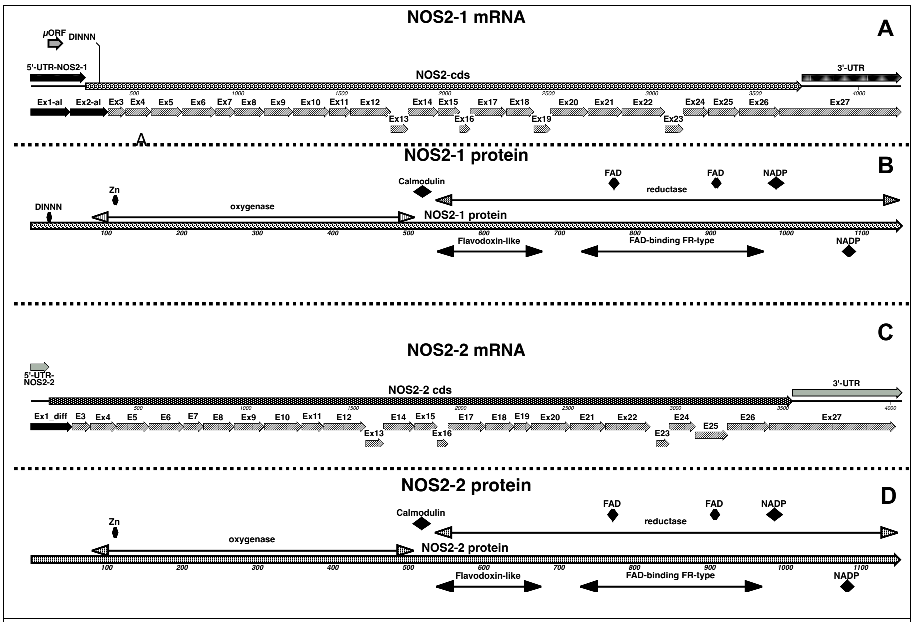

Figure S4: Scheme of the human NOS2-1 and NOS2-2 mRNA and protein. Gene as presented in the UCSC genome browser.

A: Structure of the human NOS2-1 mRNA (NM_000625.4; ENST00000313735.11). The 5' and 3'-UTR sequences, the µORF in the 5'-UTR, the exon sequences used, and the coding sequence (**cds**) is shown. Also, the **DINNN** motif important for proteasomal degradation [4] is indicated.

B: Structure of the human NOS2-1 protein (ENSP00000327251.6). The **oxygenase**-domain, and the **reductase**-domain are indicated. In addition, the binding (**bdg**) sites for **Zinc**, **Calmodulin, FMN**, **FAD**, and **NADP** are shown. Also, the amino terminal **DINNN** site is displayed.

C: Structure of the human NOS2-2 mRNA (ENST00000646938.1). The 5' and 3'-UTR sequences, the exon sequences used, and the coding sequence (**cds**) are shown. In this isoform exons 1 and 2 are replaced by exon 1-diff (**E1-diff**) encoding for a NOS2-2 mRNA specific 5'-UTR sequence.

hat gelöscht: gene

D: Structure of the human NOS2-2 protein (ENSP00000494870.1). The NOS2-2 protein has the nearly the same structure as the NOS2-1 protein. However, as the amino terminal end of the protein is different the DINNN-motif is missing.

hat gelöscht: aminoterminal

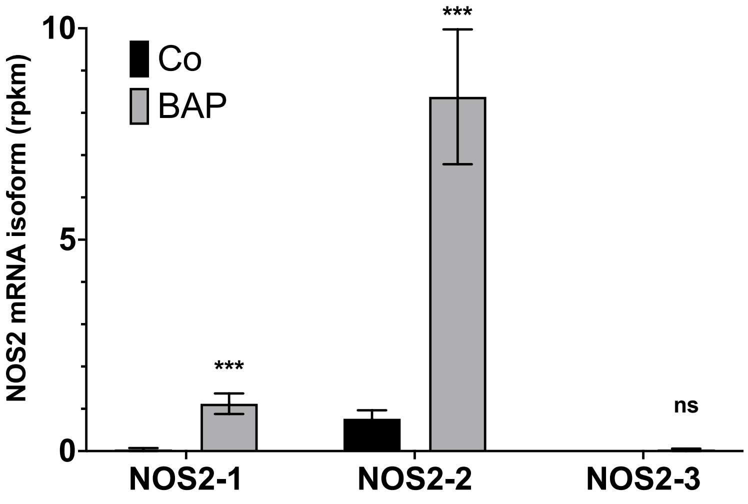

Figure S5: NOS2 mRNA isoform expression in human H1-ESC induced to differentiate to synctiotrophoblast cells.

RNA-Seq data (Bioproject PRJNA294733, [5, 6]) were analyzed for human NOS2 mRNA isoform expression. Shown are the mean ± sem of the rpkm values of NOS2 mRNA isoforms treated with **(BAP**) or without (**co**) treatment of the cells with bone morphogenetic protein-4 (BMP-4), BMP4/A83-01 (activin A signaling inhibitor) and PD173074 (FGF2 signaling inhibitor; BAP treatment). *** FDR p-value < 0.001 vs untreated ESC.

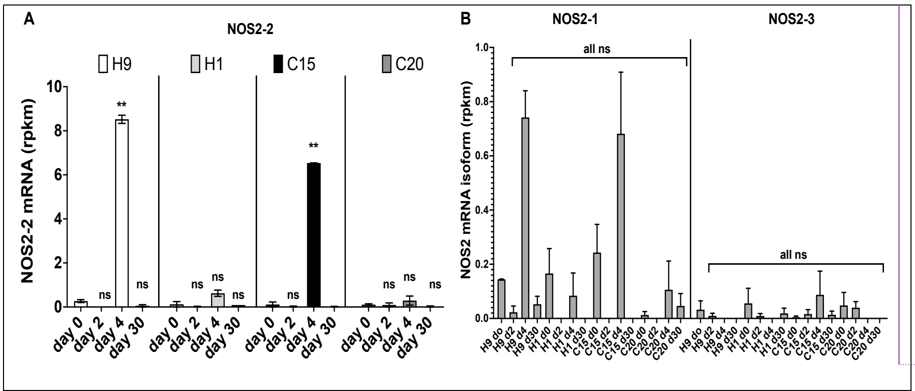

Figure S6: NOS2-2 mRNA expression in two different human ESC lines (H1, H9) and two different iPSC (C15, C20) lines induced to differentiate to cardiomyocytes.

RNA-Seq data (Bioproject PRJNA338181 [7]) were analyzed for human NOS2 mRNA isoform expression (**A**: NOS2-2; **B**: NOS2-1 and NOS2-3). Shown are the mean ± SEM of the rpkm values of NOS2 mRNA isoforms at different time points (day 0 to day 30). ** FDR p-value < 0.01; ns FDR-value > 0,05 vs day 0.

Kommentiert [PA1]: x-Achsenbeschriftung ist schlecht zu lesen

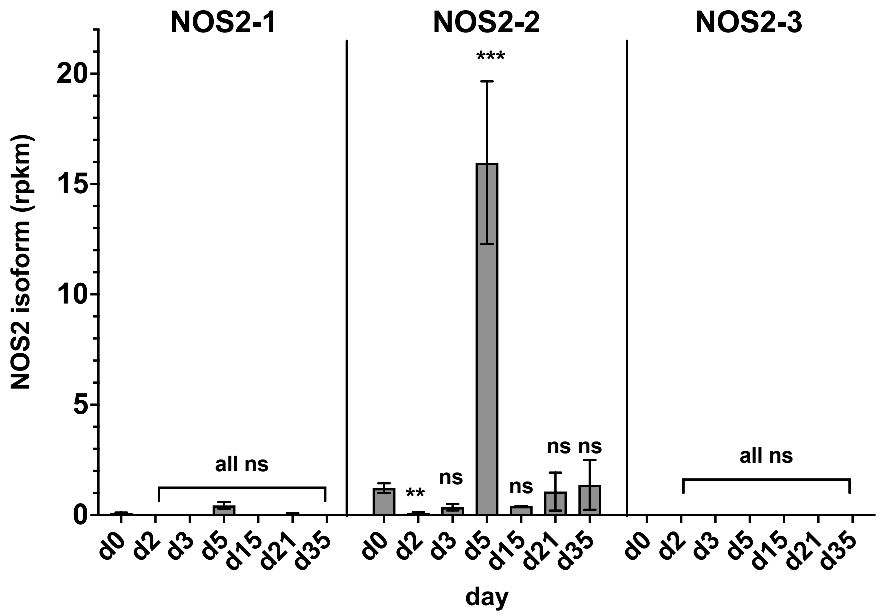

Figure S7: NOS2 mRNA isoform expression in human CD34-iPSC induced to differentiate to cardiomyocytes.

RNA-Seq data (Bioproject PRJNA674506 [8]) were analyzed for human NOS2 mRNA isoform expression. Shown are the mean ± sem of the rpkm values of NOSS2 mRNA isoforms at different time points (day 0 to day 35). *** FDR-value < 0.001, ** FDR-value < 0.01, ns FDR-value > 0.05 vs day 0.

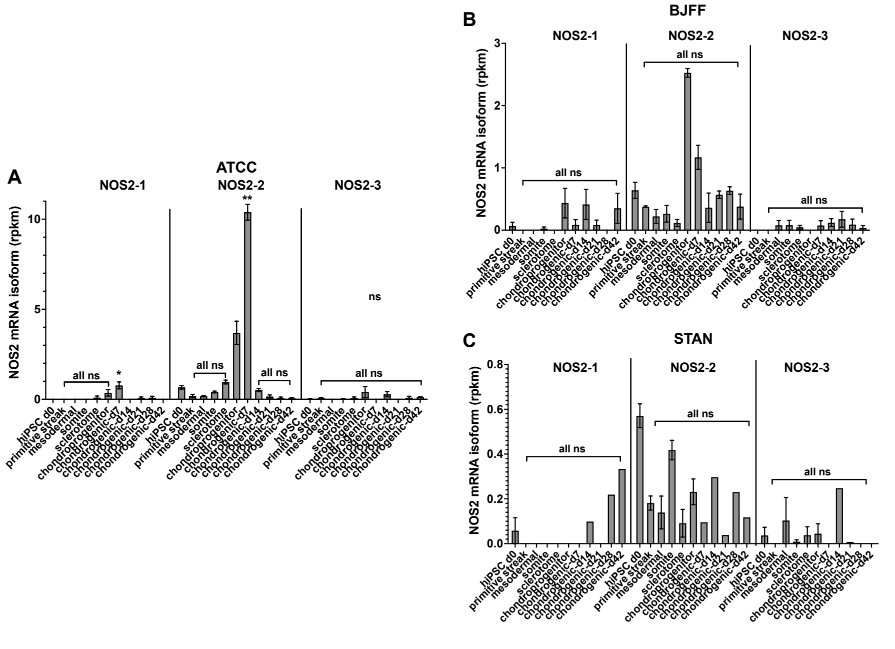

Figure S8: NOS2 mRNA isoform expression in different human iPSC lines (ATCC, BJFF, STAN) induced to differentiate to chondrocytes.

RNA-Seq data (Bioproject PRJNA674506 [9]) were analyzed for human NOS2 mRNA isoform expression. Shown are the mean ± SEM of the rpkm values of the NOS2 mRNA isoforms at different differentiation stages (untreated **hiPSC d0**, primitive **streak, meso**derm**, som**ite**, sclero**tome**,** chondroprogenitor – **CP -,** and chondrogenic **– CG** - from d7 to day 42). ** FDR p-value < 0.01, ns FDR-value > 0.05 vs day 0

hat gelöscht: .

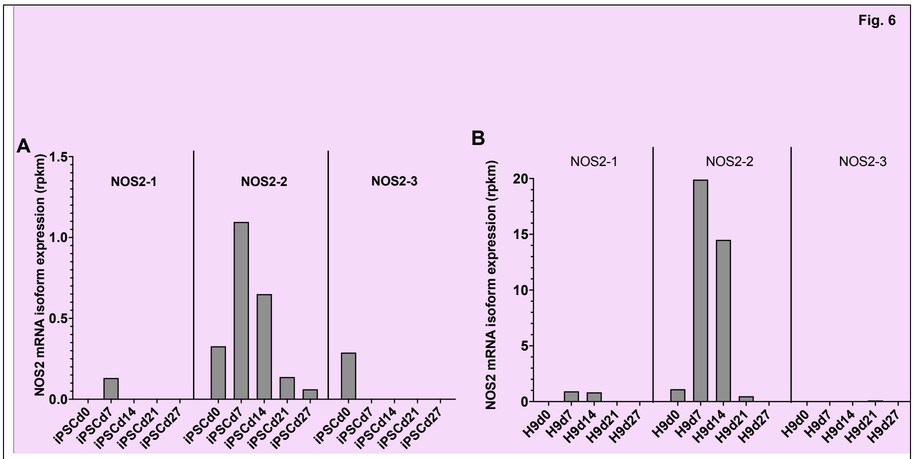

Figure S9: NOS2 mRNA isoform expression in human H9-ESC or iPSC induced to differentiate to mesenchymal stromal cells.

RNA-Seq data (CNP0000771 [10]) were analyzed for human NOS2 mRNA isoform expression. Shown are rpkm values (single experiments) of the NOS2 mRNA isoforms at different time points (day 0 to day 27).

Kommentiert [PA2]: x-Achsenbeschriftung bei B schwierig zu lesen

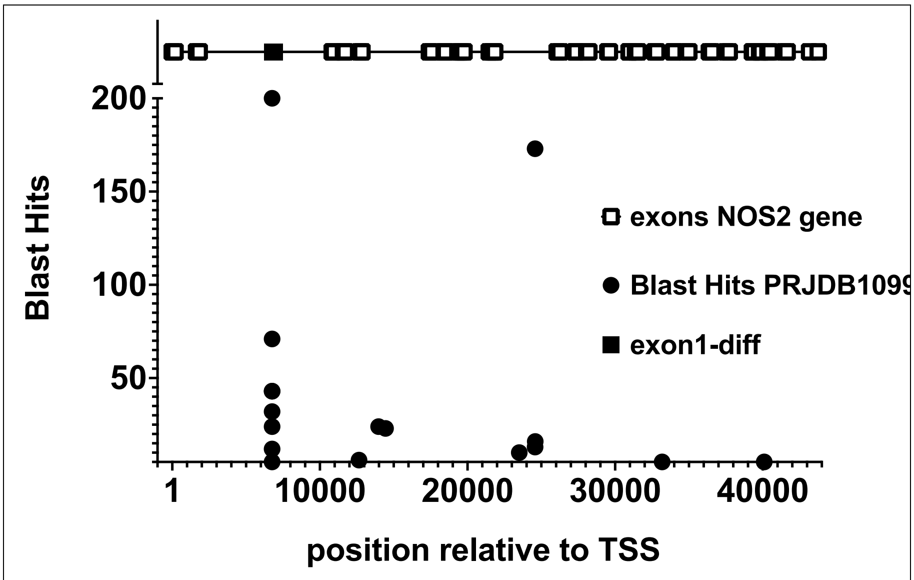

Figure S10: Blast hits of the sequence reads from PRJB1099 on the human NOS2 gene. RNA-Seq data (Bioproject PRJB1099 [11]) were aligned to the human NOS2 gene. Shown are the number of blast hits and the position of the exons of the human NOS2 gene relative to the transcription start site (TSS) of the humane NOS2 gene.

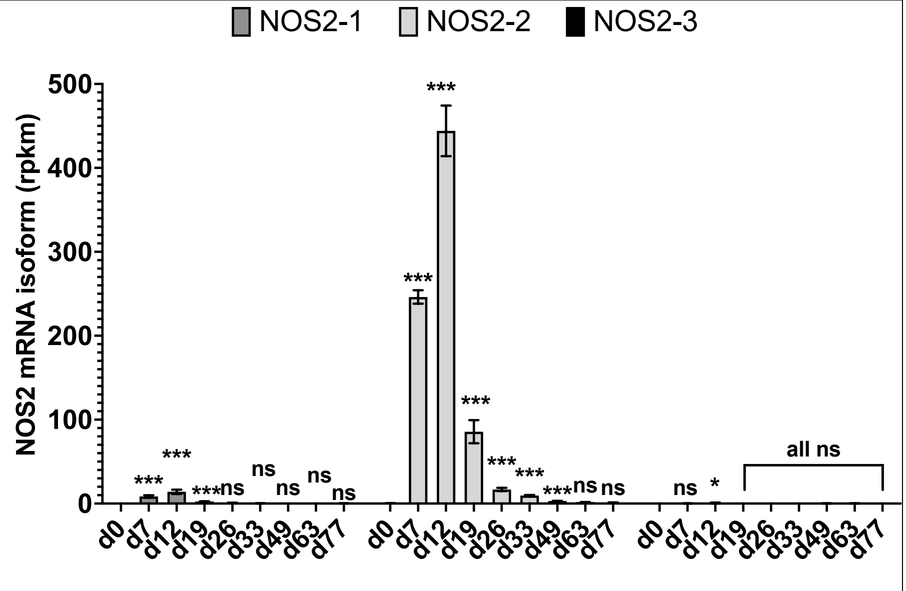

Figure S11: NOS2 mRNA isoform expression in human H9 ESC induced to differentiate to neurons.

RNA-Seq data (Bioproject PRJNA244622 [12]) were analyzed for human NOS2 mRNA isoform expression. Shown are the mean ± sem of the rpkm values of NOS2 mRNA isoform at different time points (day 0 to day 77). *** FDR p-value < 0.001, * FDR-value < 0.05, ns FDR-value > 0.05 vs day 0.

hat gelöscht: S

hat gelöscht:

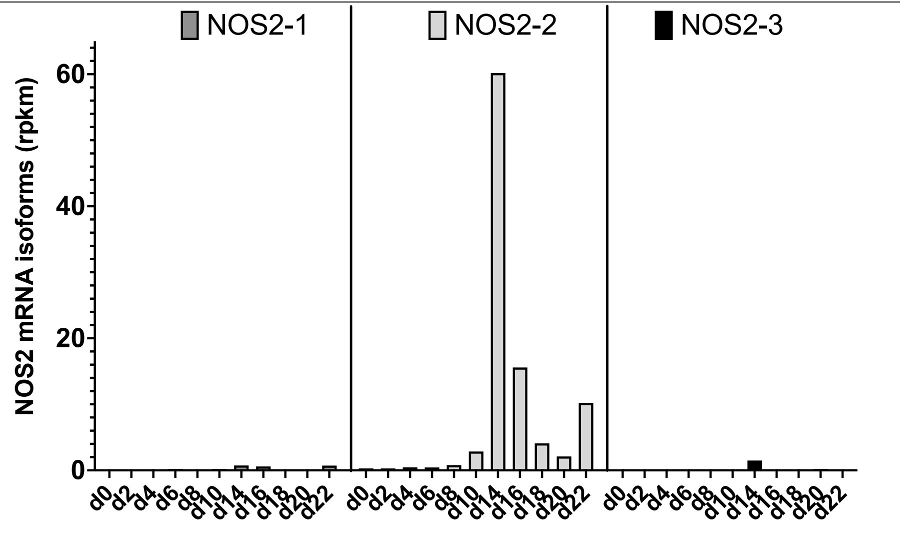

Figure S12: NOS2 mRNA isoform expression in H9-ESC induced to differentiate to neurons.

RNA-Seq data (Bioproject PRJNA404971, [7]) were analyzed for human NOS2 mRNA isoform expression. Shown are the single rpkm values of NOSS2 mRNA isoforms at different time points (day 0 to day 22).

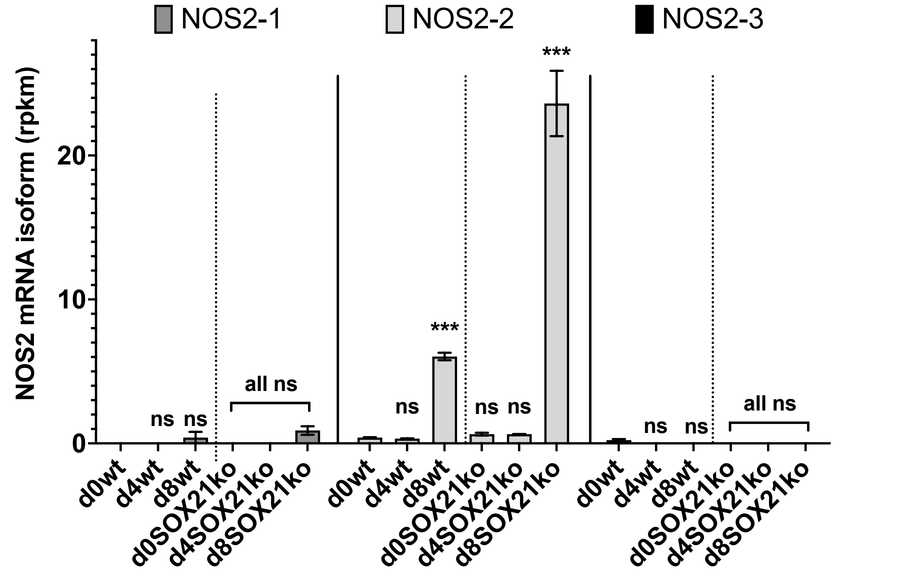

Figure S13: NOS2 mRNA isoform expression in human wildtype H9-ESC (**wt**) or H9-ESC stably transfected with an anti-SOX21 siRNA (**SOX21ko**) induced to differentiate to neurons.

RNA-Seq data (Bioproject PRJNA433877, [13]) were analyzed for human NOS2 mRNA isoform expression. Shown are the mean ± sem of the rpkm values of NOSS2 mRNA isoforms at different time points (day 0 to day 21). *** FDR p-value < 0.001, ns FDR value > 0,05 vs day 0 wt.

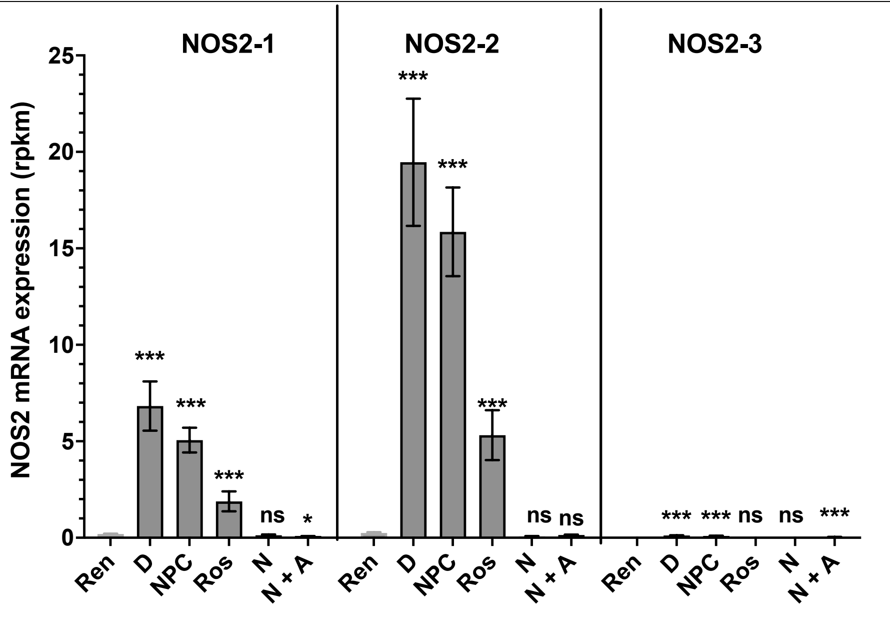

Figure S14: NOS2 mRNA isoform expression in human iPSC induced to differentiate to neurons.

RNA-Seq data (Bioproject PRJNA596331) were analyzed for human NOS2 mRNA isoform expression. Shown are the mean ± sem of the rpkm values of NOSS2 mRNA isoforms at different differentiation stages (Ren = renew, D = dorsal, NPS = neuronal progenitor cells, Ros = rosette, N = neurons, N+A = neurons plus astrocytes). *** FDR p-value < 0.001, * FDR-Value < 0.05, ns FDR value > 0,05 vs Ren.

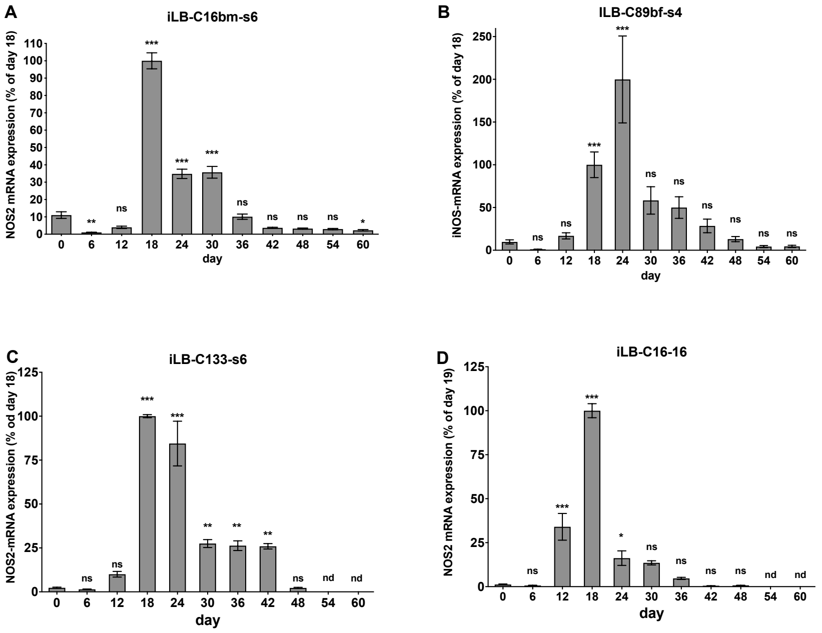

Figure S15: NOS2 mRNA expression in human iPSC induced to differentiate to neurons. Four different human iPSCs lines were generated from PBMC of three different donors [14]. RNA was isolated from these 4 different human iPSC lines induced to differentiate to neurons [15] at different time points (**day 0 to 60**). NOS2-mRNA and 18S rRNA expression was analyzed using the qRT-PCR method. NOS2 mRNA expression was normalized to the 18S rRNA expression. The relative NOS2 mRNA expression in the cells treated for 18 days was set to 100%. Shown are the individual summaries of the analysis of the four different iPSC cell lines The values represent the mean ± SEM of n=3 different isolated RNAs for each time point. (*** p < 0.001, ** p < 0,01, * p < 0.05, ns not significant vs. iPSC treated for zero days;1-way Anova with Bonferroni's multiple comparisons test).

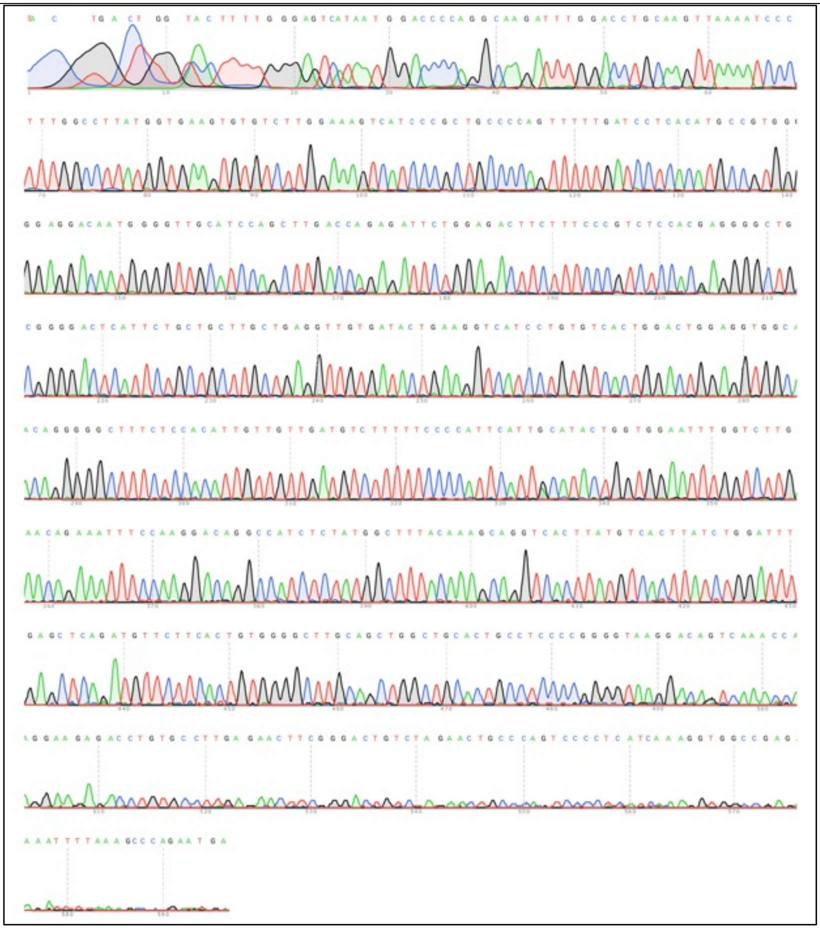

A

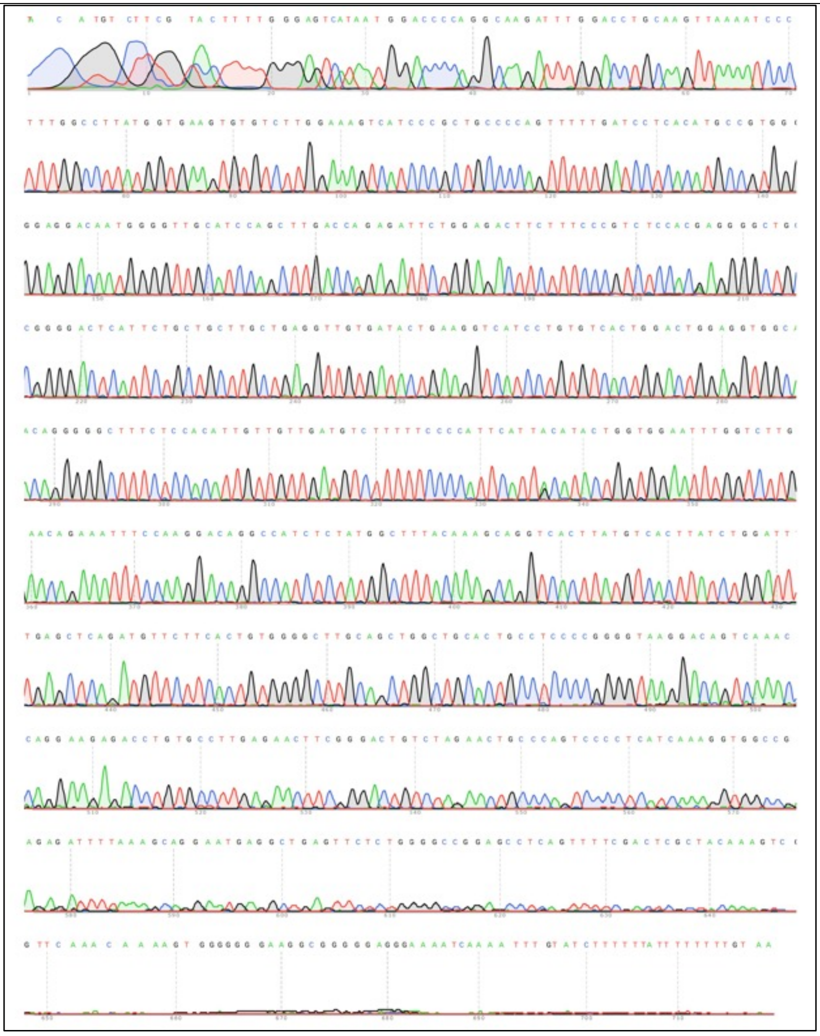

B

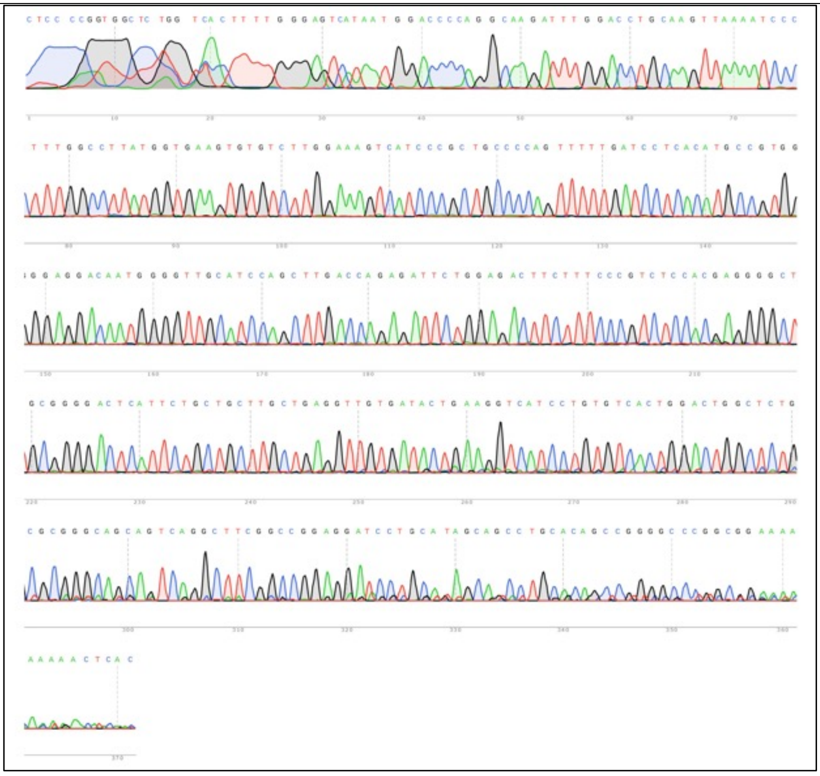

C

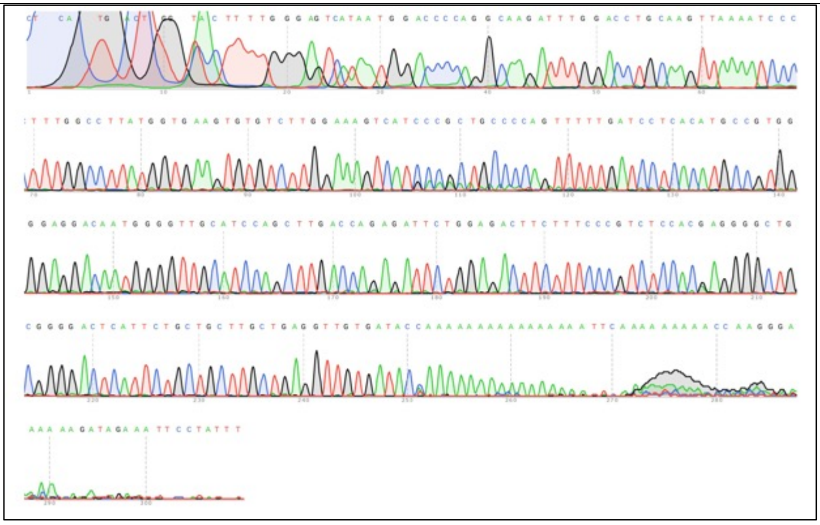

D

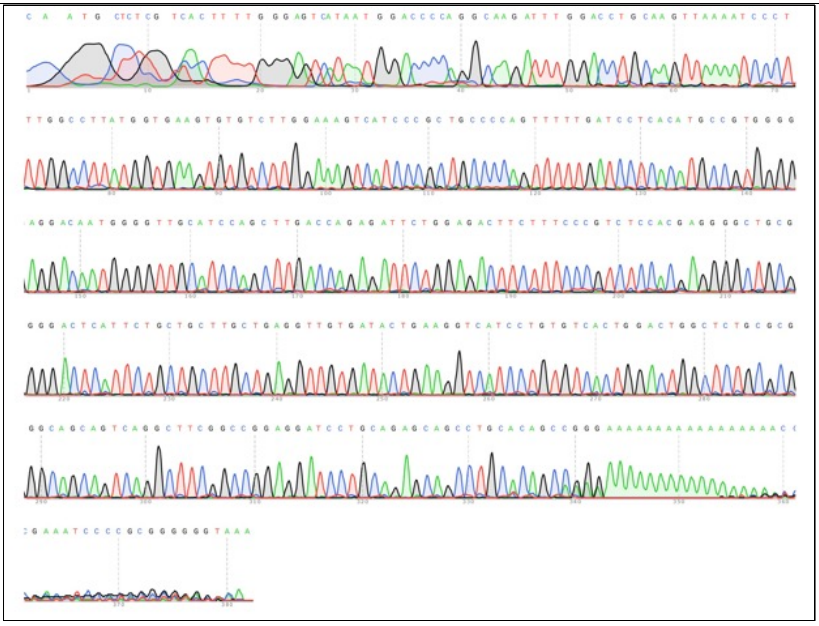

E

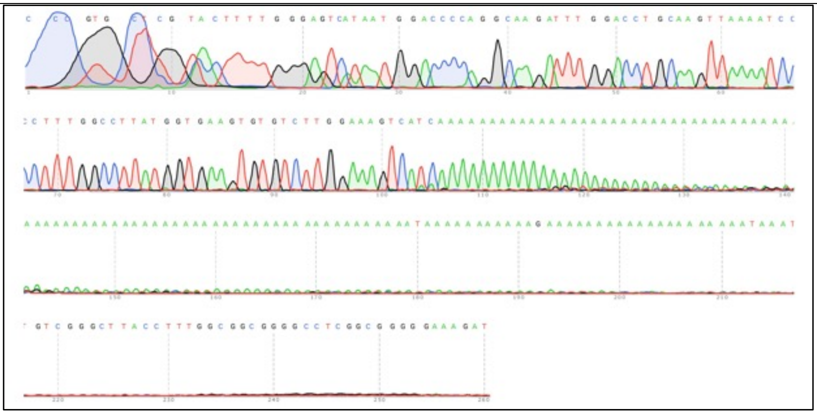

F

Figure S16: Sequencing results of the fragments obtained by the 5'-RACE PCR.

Shown are the results of the sequencing reaction of the following fragments obtained in the 5'-RACE analyses (see figure 11): **A**: fragment DLD1-2, **B**: fragment DLD1-3, **C**: fragment C16-1, **E**: fragment C16-2, **F**: fragment C89-2, **G:** fragment C89-3

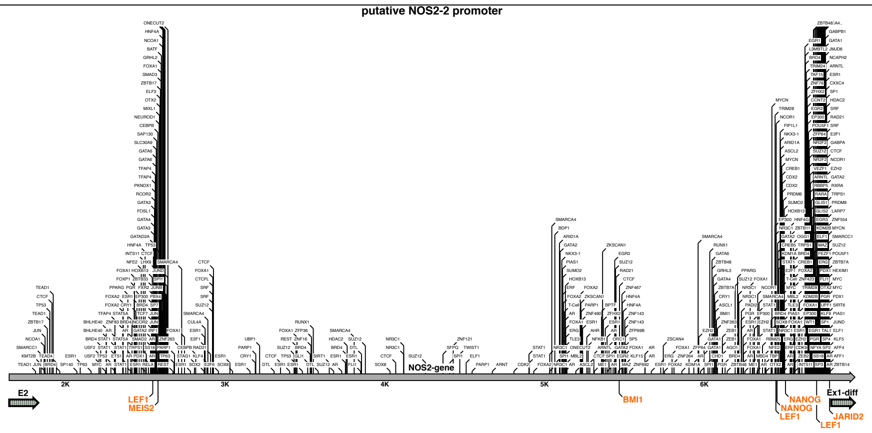

Figure S17: Scheme of the putative alternative promoter used for the expression of the NOS2-2 mRNA.

Shown are the transcription factor binding sites (TF-bdg) to the genomic DNA sequence between exon 2 and exon 1-diff of the human NOS2 gene. All TF-bdg shown (see supplementary table S1 for description) are identified by ChIP analyses [1]. Indicated are the exon 2 and exon 1-diff sequences. The highlighted TF-bdg (BMI1, JALEF1, MEIS2, NANOG) are transcription factors showing enhanced expression in all expression data analyzed (see supplemental table S2)

Supplemental References

1. Kolpakov F, Akberdin I, Kashapov T, Kiselev L, Kolmykov S, Kondrakhin Y, Kutumova E, Mandrik N, Pintus S, Ryabova A, et al: **BioUML: an integrated environment for systems biology and collaborative analysis of biomedical data.** *Nucleic Acids Res* 2019, **47:**W225-W233.
2. Vaquerizas JM, Kummerfeld SK, Teichmann SA, Luscombe NM: **A census of human transcription factors: function, expression and evolution.** *Nat Rev Genet* 2009, **10:**252-263.
3. Gerstberger S, Hafner M, Tuschl T: **A census of human RNA-binding proteins.** *Nat Rev Genet* 2014, **15:**829-845.
4. Foster MW, Thompson JW, Forrester MT, Sha Y, McMahon TJ, Bowles DE, Moseley MA, Marshall HE: **Proteomic analysis of the NOS2 interactome in human airway epithelial cells.** *Nitric Oxide* 2013, **34:**37-46.
5. Schust DJ, Bonney EA, Sugimoto J, Ezashi T, Roberts RM, Choi S, Zhou J: **The Immunology of Syncytialized Trophoblast.** *Int J Mol Sci* 2021, **22**.
6. Yabe S, Alexenko AP, Amita M, Yang Y, Schust DJ, Sadovsky Y, Ezashi T, Roberts RM: **Comparison of syncytiotrophoblast generated from human embryonic stem cells and from term placentas.** *Proc Natl Acad Sci U S A* 2016, **113:**E2598-2607.
7. Liu Q, Jiang C, Xu J, Zhao MT, Van Bortle K, Cheng X, Wang G, Chang HY, Wu JC, Snyder MP: **Genome-Wide Temporal Profiling of Transcriptome and Open Chromatin of Early Cardiomyocyte Differentiation Derived From hiPSCs and hESCs.** *Circ Res* 2017, **121:**376-391.
8. Zhang F, Qiu H, Dong X, Wang C, Na J, Zhou J, Wang C: **Transferrin improved the generation of cardiomyocyte from human pluripotent stem cells for myocardial infarction repair.** *J Mol Histol* 2021, **52:**87-99.

9. Wu CL, Dicks A, Steward N, Tang R, Katz DB, Choi YR, Guilak F: **Single cell transcriptomic analysis of human pluripotent stem cell chondrogenesis.** *Nat Commun* 2021, **12:**362.
10. Luo L, Zhou Y, Zhang C, Huang J, Du J, Liao J, Bergholt NL, Bunger C, Xu F, Lin L, et al: **Feeder-free generation and transcriptome characterization of functional mesenchymal stromal cells from human pluripotent stem cells.** *Stem Cell Res* 2020, **48:**101990.
11. Hon CC, Ramilowski JA, Harshbarger J, Bertin N, Rackham OJ, Gough J, Denisenko E, Schmeier S, Poulsen TM, Severin J, et al: **An atlas of human long non-coding RNAs with accurate 5' ends.** *Nature* 2017, **543:**199-204.
12. van de Leemput J, Boles NC, Kiehl TR, Corneo B, Lederman P, Menon V, Lee C, Martinez RA, Levi BP, Thompson CL, et al: **CORTECON: a temporal transcriptome analysis of in vitro human cerebral cortex development from human embryonic stem cells.** *Neuron* 2014, **83:**51-68.
13. Fang Z, Liu X, Wen J, Tang F, Zhou Y, Jing N, Jin Y: **SOX21 Ensures Rostral Forebrain Identity by Suppression of WNT8B during Neural Regionalization of Human Embryonic Stem Cells.** *Stem Cell Reports* 2019, **13:**1038-1052.
14. Peitz M, Bechler T, Thiele CC, Veltel M, Bloschies M, Fliessbach K, Ramirez A, Brustle O: **Blood-derived integration-free iPS cell line UKBi011-A from a diagnosed male Alzheimer's disease patient with APOE varepsilon4/varepsilon4 genotype.** *Stem Cell Res* 2018, **29:**250-253.
15. Rehbach K, Kesavan J, Hauser S, Ritzenhofen S, Jungverdorben J, Schule R, Schols L, Peitz M, Brustle O: **Multiparametric rapid screening of neuronal process pathology for drug target identification in HSP patient-specific neurons.** *Sci Rep* 2019, **9:**9615.
